# Discovering faster matrix multiplication algorithms with reinforcement learning

**DOI:** 10.1038/s41586-022-05172-4

**Published:** 2022-10-05

**Authors:** Alhussein Fawzi, Matej Balog, Aja Huang, Thomas Hubert, Bernardino Romera-Paredes, Mohammadamin Barekatain, Alexander Novikov, Francisco J. R. Ruiz, Julian Schrittwieser, Grzegorz Swirszcz, David Silver, Demis Hassabis, Pushmeet Kohli

**Affiliations:** DeepMind, London, UK

**Keywords:** Applied mathematics, Computer science

## Abstract

Improving the efficiency of algorithms for fundamental computations can have a widespread impact, as it can affect the overall speed of a large amount of computations. Matrix multiplication is one such primitive task, occurring in many systems—from neural networks to scientific computing routines. The automatic discovery of algorithms using machine learning offers the prospect of reaching beyond human intuition and outperforming the current best human-designed algorithms. However, automating the algorithm discovery procedure is intricate, as the space of possible algorithms is enormous. Here we report a deep reinforcement learning approach based on AlphaZero^1^ for discovering efficient and provably correct algorithms for the multiplication of arbitrary matrices. Our agent, AlphaTensor, is trained to play a single-player game where the objective is finding tensor decompositions within a finite factor space. AlphaTensor discovered algorithms that outperform the state-of-the-art complexity for many matrix sizes. Particularly relevant is the case of 4 × 4 matrices in a finite field, where AlphaTensor’s algorithm improves on Strassen’s two-level algorithm for the first time, to our knowledge, since its discovery 50 years ago^2^. We further showcase the flexibility of AlphaTensor through different use-cases: algorithms with state-of-the-art complexity for structured matrix multiplication and improved practical efficiency by optimizing matrix multiplication for runtime on specific hardware. Our results highlight AlphaTensor’s ability to accelerate the process of algorithmic discovery on a range of problems, and to optimize for different criteria.

## Abstract (web summary)

A reinforcement learning approach based on AlphaZero is used to discover efficient and provably correct algorithms for matrix multiplication, finding faster algorithms for a variety of matrix sizes.

## Main

We focus on the fundamental task of matrix multiplication, and use deep reinforcement learning (DRL) to search for provably correct and efficient matrix multiplication algorithms. This algorithm discovery process is particularly amenable to automation because a rich space of matrix multiplication algorithms can be formalized as low-rank decompositions of a specific three-dimensional (3D) tensor^2^, called the matrix multiplication tensor^3–7^. This space of algorithms contains the standard matrix multiplication algorithm and recursive algorithms such as Strassen’s^2^, as well as the (unknown) asymptotically optimal algorithm. Although an important body of work aims at characterizing the complexity of the asymptotically optimal algorithm^8–12^, this does not yield practical algorithms^5^. We focus here on practical matrix multiplication algorithms, which correspond to explicit low-rank decompositions of the matrix multiplication tensor. In contrast to two-dimensional matrices, for which efficient polynomial-time algorithms computing the rank have existed for over two centuries^13^, finding low-rank decompositions of 3D tensors (and beyond) is NP-hard^14^ and is also hard in practice. In fact, the search space is so large that even the optimal algorithm for multiplying two 3 × 3 matrices is still unknown. Nevertheless, in a longstanding research effort, matrix multiplication algorithms have been discovered by attacking this tensor decomposition problem using human search^2,15,16^, continuous optimization^17–19^ and combinatorial search^20^. These approaches often rely on human-designed heuristics, which are probably suboptimal. We instead use DRL to learn to recognize and generalize over patterns in tensors, and use the learned agent to predict efficient decompositions.

We formulate the matrix multiplication algorithm discovery procedure (that is, the tensor decomposition problem) as a single-player game, called TensorGame. At each step of TensorGame, the player selects how to combine different entries of the matrices to multiply. A score is assigned based on the number of selected operations required to reach the correct multiplication result. This is a challenging game with an enormous action space (more than 10^12^ actions for most interesting cases) that is much larger than that of traditional board games such as chess and Go (hundreds of actions). To solve TensorGame and find efficient matrix multiplication algorithms, we develop a DRL agent, AlphaTensor. AlphaTensor is built on AlphaZero^1,21^, where a neural network is trained to guide a planning procedure searching for efficient matrix multiplication algorithms. Our framework uses a single agent to decompose matrix multiplication tensors of various sizes, yielding transfer of learned decomposition techniques across various tensors. To address the challenging nature of the game, AlphaTensor uses a specialized neural network architecture, exploits symmetries of the problem and makes use of synthetic training games.

AlphaTensor scales to a substantially larger algorithm space than what is within reach for either human or combinatorial search. In fact, AlphaTensor discovers from scratch many provably correct matrix multiplication algorithms that improve over existing algorithms in terms of number of scalar multiplications. We also adapt the algorithm discovery procedure to finite fields, and improve over Strassen’s two-level algorithm for multiplying 4 × 4 matrices for the first time, to our knowledge, since its inception in 1969. AlphaTensor also discovers a diverse set of algorithms—up to thousands for each size—showing that the space of matrix multiplication algorithms is richer than previously thought. We also exploit the diversity of discovered factorizations to improve state-of-the-art results for large matrix multiplication sizes. Through different use-cases, we highlight AlphaTensor’s flexibility and wide applicability: AlphaTensor discovers efficient algorithms for structured matrix multiplication improving over known results, and finds efficient matrix multiplication algorithms tailored to specific hardware, by optimizing for actual runtime. These algorithms multiply large matrices faster than human-designed algorithms on the same hardware.

## Algorithms as tensor decomposition

**Fig. 1:**
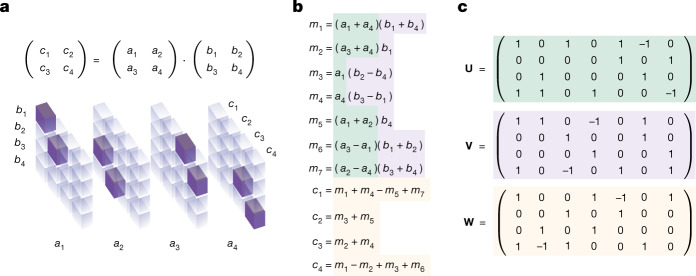
Matrix multiplication tensor and algorithms. **a**, Tensor ${{\mathscr{T}}}_{2}$ representing the multiplication of two 2 × 2 matrices. Tensor entries equal to 1 are depicted in purple, and 0 entries are semi-transparent. The tensor specifies which entries from the input matrices to read, and where to write the result. For example, as *c*_1_ = *a*_1_*b*_1_ + *a*_2_*b*_3_, tensor entries located at (*a*_1_, *b*_1_, *c*_1_) and (*a*_2_, *b*_3_, *c*_1_) are set to 1. **b**, Strassen's algorithm^2^ for multiplying 2 × 2 matrices using 7 multiplications. **c**, Strassen's algorithm in tensor factor representation. The stacked factors **U**, **V** and **W** (green, purple and yellow, respectively) provide a rank-7 decomposition of ${{\mathscr{T}}}_{2}$ (equation (1)). The correspondence between arithmetic operations (**b**) and factors (**c**) is shown by using the aforementioned colours.

As matrix multiplication (**A**, **B**) ↦ **AB** is bilinear (that is, linear in both arguments), it can be fully represented by a 3D tensor: see Fig. 1a for how to represent the 2 × 2 matrix multiplication operation as a 3D tensor of size 4 × 4 × 4, and refs. ^3,5,7^ for more details. We write ${{\mathscr{T}}}_{n}$ for the tensor describing *n* × *n* matrix multiplication. The tensor ${{\mathscr{T}}}_{n}$ is fixed (that is, it is independent of the matrices to be multiplied), has entries in {0, 1}, and is of size *n*^2^ × *n*^2^ × *n*^2^. More generally, we use ${{\mathscr{T}}}_{n,m,p}$ to describe the rectangular matrix multiplication operation of size *n* × *m* with *m* × *p* (note that ${{\mathscr{T}}}_{n}={{\mathscr{T}}}_{n,n,n}$). By a decomposition of ${{\mathscr{T}}}_{n}$ into *R* rank-one terms, we mean

1 $${{\mathscr{T}}}_{n}=\mathop{\sum }\limits_{r=1}^{R}{{\bf{u}}}^{(r)}\otimes {{\bf{v}}}^{(r)}\otimes {{\bf{w}}}^{(r)},$$

where ⊗ denotes the outer (tensor) product, and **u**^(*r*)^, **v**^(*r*)^ and **w**^(*r*)^ are all vectors. If a tensor ${\mathscr{T}}$ can be decomposed into *R* rank-one terms, we say the rank of ${\mathscr{T}}$ is at most *R*, or $\,\text{Rank}\,({\mathscr{T}}\,)\le R$. This is a natural extension from the matrix rank, where a matrix is decomposed into ${\sum }_{r=1}^{R}{{\bf{u}}}^{(r)}\otimes {{\bf{v}}}^{(r)}$.

A decomposition of ${{\mathscr{T}}}_{n}$ into *R* rank-one terms provides an algorithm for multiplying arbitrary *n* × *n* matrices using *R* scalar multiplications (see Algorithm 1). We refer to Fig. 1b,c for an example algorithm multiplying 2 × 2 matrices with *R* = 7 (Strassen’s algorithm).

Crucially, Algorithm 1 can be used to multiply block matrices. By using this algorithm recursively, one can multiply matrices of arbitrary size, with the rank *R* controlling the asymptotic complexity of the algorithm. In particular, *N* × *N* matrices can be multiplied with asymptotic complexity ${\mathcal{O}}({N}^{{\log }_{n}(R)})$; see ref. ^5^ for more details.

Algorithm 1 A meta-algorithm parameterized by ${\{{{\bf{u}}}^{(r)},{{\bf{v}}}^{(r)},{{\bf{w}}}^{(r)}\}}_{r=1}^{R}$ for computing the matrix product **C** = **AB**. It is noted that *R* controls the number of multiplications between input matrix entries.

Parameters: ${\{{{\bf{u}}}^{(r)},{{\bf{v}}}^{(r)},{{\bf{w}}}^{(r)}\}}_{r=1}^{R}$: length-*n*^2^ vectors such that ${{\mathscr{T}}}_{n}={\sum }_{r=1}^{R}{{\bf{u}}}^{(r)}\otimes {{\bf{v}}}^{(r)}\otimes {{\bf{w}}}^{(r)}$

Input: **A**, **B**: matrices of size *n* × *n*

Output: **C** = **AB**

(1) **for** *r* = 1, …, *R* **do**

(2)     $${m}_{r}\leftarrow \left({u}_{1}^{(r)}{a}_{1}+\cdots +{u}_{{n}^{2}}^{(r)}{a}_{{n}^{2}}\right)\left({v}_{1}^{(r)}{b}_{1}+\cdots +{v}_{{n}^{2}}^{(r)}{b}_{{n}^{2}}\right)$$

(3) **for** *i* = 1, …, *n*^2^ **do**

(4)     $${c}_{i}\leftarrow {w}_{i}^{(1)}{m}_{1}+\cdots +{w}_{i}^{(R)}{m}_{R}$$

return **C**

## DRL for algorithm discovery

We cast the problem of finding efficient matrix multiplication algorithms as a reinforcement learning problem, modelling the environment as a single-player game, TensorGame. The game state after step *t* is described by a tensor ${{\mathscr{S}}}_{t}$, which is initially set to the target tensor we wish to decompose: ${{\mathscr{S}}}_{0}={{\mathscr{T}}}_{n}$. In each step *t* of the game, the player selects a triplet (**u**^(*t*)^, **v**^(*t*)^, **w**^(*t*)^), and the tensor ${{\mathscr{S}}}_{t}$ is updated by subtracting the resulting rank-one tensor: ${{\mathscr{S}}}_{t}\leftarrow {{\mathscr{S}}}_{t-1}-{{\bf{u}}}^{(t)}\otimes {{\bf{v}}}^{(t)}\otimes {{\bf{w}}}^{(t)}$. The goal of the player is to reach the zero tensor ${{\mathscr{S}}}_{t}={\bf{0}}$ by applying the smallest number of moves. When the player reaches the zero tensor, the sequence of selected factors satisfies ${{\mathscr{T}}}_{n}={\sum }_{t=1}^{R}{{\bf{u}}}^{(t)}\otimes {{\bf{v}}}^{(t)}\otimes {{\bf{w}}}^{(t)}$ (where *R* denotes the number of moves), which guarantees the correctness of the resulting matrix multiplication algorithm. To avoid playing unnecessarily long games, we limit the number of steps to a maximum value, *R*_limit_.

For every step taken, we provide a reward of −1 to encourage finding the shortest path to the zero tensor. If the game terminates with a non-zero tensor (after *R*_limit_ steps), the agent receives an additional terminal reward equal to $-\gamma ({{\mathscr{S}}}_{{R}_{\text{limit}}})$, where $\gamma ({{\mathscr{S}}}_{{R}_{\text{limit}}})$ is an upper bound on the rank of the terminal tensor. Although this reward optimizes for rank (and hence for the complexity of the resulting algorithm), other reward schemes can be used to optimize other properties, such as practical runtime (see ‘Algorithm discovery results’). Besides, as our aim is to find exact matrix multiplication algorithms, we constrain {**u**^(*t*)^, **v**^(*t*)^, **w**^(*t*)^} to have entries in a user-specified discrete set of coefficients *F* (for example, *F* = {−2, −1, 0, 1, 2}). Such discretization is common practice to avoid issues with the finite precision of floating points^15,18,20^.

**Fig. 2:**
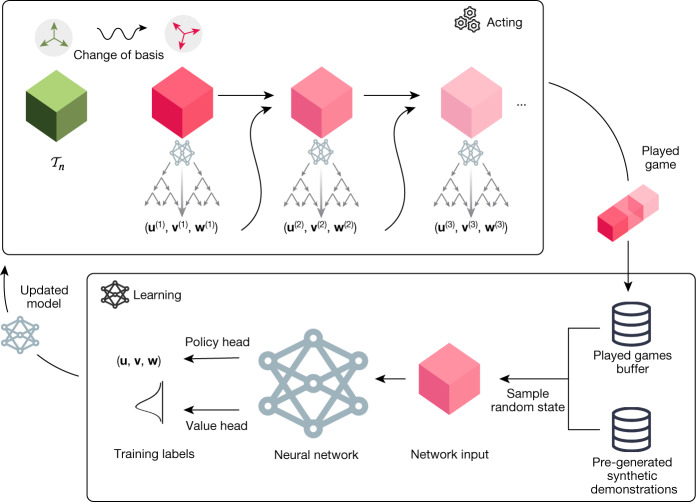
Overview of AlphaTensor. The neural network (bottom box) takes as input a tensor ${{\mathscr{S}}}_{t}$, and outputs samples (**u**, **v**, **w**) from a distribution over potential next actions to play, and an estimate of the future returns (for example, of $-{\rm{Rank}}\,({{\mathscr{S}}}_{t})$). The network is trained on two data sources: previously played games and synthetic demonstrations. The updated network is sent to the actors (top box), where it is used by the MCTS planner to generate new games.

To play TensorGame, we propose AlphaTensor (Fig. 2), an agent based on AlphaZero^1^, which achieved *tabula rasa* superhuman performance in the classical board games of Go, chess and shogi, and on its extension to handle large action spaces Sampled AlphaZero^21^. Similarly to AlphaZero, AlphaTensor uses a deep neural network to guide a Monte Carlo tree search (MCTS) planning procedure. The network takes as input a state (that is, a tensor ${{\mathscr{S}}}_{t}$ to decompose), and outputs a policy and a value. The policy provides a distribution over potential actions. As the set of potential actions (**u**^(*t*)^, **v**^(*t*)^, **w**^(*t*)^) in each step is enormous, we rely on sampling actions rather than enumerating them^21,22^. The value provides an estimate of the distribution *z* of returns (cumulative reward) starting from the current state ${{\mathscr{S}}}_{t}$. With the above reward scheme, the distribution *z* models the agent’s belief about the rank of the tensor ${{\mathscr{S}}}_{t}$. To play a game, AlphaTensor starts from the target tensor (${{\mathscr{T}}}_{n}$) and uses the MCTS planner at each step to choose the next action. Finished games are used as feedback to the network to improve the network parameters.

Overcoming the challenges posed by TensorGame—namely, an enormous action space, and game states described by large 3D tensors representing an abstract mathematical operation—requires multiple advances. All these components, described briefly below,  substantially improve the overall performance over a plain AlphaZero agent (see Methods and Supplementary Information for details).

### Neural network architecture

We propose a transformer-based^23^ architecture that incorporates inductive biases for tensor inputs. We first project the *S* × *S* × *S* input tensor into three *S* × *S* grids of feature vectors by using linear layers applied to the three cyclic transpositions of the tensor. The main part of the model comprises a sequence of attention operations, each applied to a set of features belonging to a pair of grids (Extended Data Figs. 3 and 4). This generalizes axial attention^24^ to multiple grids, and is both more efficient and yields better results than naive self-attention. The proposed architecture, which disregards the order of rows and columns in the grids, is inspired by the invariance of the tensor rank to slice reordering. The final feature representation of the three matrices is passed both to the policy head (an autoregressive model) and the value head (a multilayer perceptron).

### Synthetic demonstrations

Although tensor decomposition is NP-hard, the inverse task of constructing the tensor from its rank-one factors is elementary. Hence, we generate a large dataset of tensor-factorization pairs (synthetic demonstrations) by first sampling factors ${\{({{\bf{u}}}^{(r)},{{\bf{v}}}^{(r)},{{\bf{w}}}^{(r)})\}}_{r=1}^{R}$ at random, and then constructing the tensor ${\mathscr{D}}={\sum }_{r=1}^{R}{{\bf{u}}}^{(r)}\otimes {{\bf{v}}}^{(r)}\otimes {{\bf{w}}}^{(r)}$. We train the network on a mixture of supervised loss (that is, to imitate synthetic demonstrations) and standard reinforcement learning loss (that is, learning to decompose a target tensor ${{\mathscr{T}}}_{n}$) (Fig. 2). This mixed training strategy—training on the target tensor and random tensors— substantially outperforms each training strategy separately. This is despite randomly generated tensors having different properties from the target tensors.

### Change of basis

${{\mathscr{T}}}_{n}$ (Fig. 1a) is the tensor representing the matrix multiplication bilinear operation in the canonical basis. The same bilinear operation can be expressed in other bases, resulting in other tensors. These different tensors are equivalent: they have the same rank, and decompositions obtained in a custom basis can be mapped to the canonical basis, hence obtaining a practical algorithm of the form in Algorithm 1. We leverage this observation by sampling a random change of basis at the beginning of every game, applying it to ${{\mathscr{T}}}_{n}$, and letting AlphaTensor play the game in that basis (Fig. 2). This crucial step injects diversity into the games played by the agent.

### Data augmentation

From every played game, we can extract additional tensor-factorization pairs for training the network. Specifically, as factorizations are order invariant (owing to summation), we build an additional tensor-factorization training pair by swapping a random action with the last action from each finished game.

## Algorithm discovery results

### Discovery of matrix multiplication algorithms

We train a single AlphaTensor agent to find matrix multiplication algorithms for matrix sizes *n* × *m* with *m* × *p*, where *n*, *m*, *p* ≤ 5. At the beginning of each game, we sample uniformly a triplet (*n*, *m*, *p*) and train AlphaTensor to decompose the  tensor ${{\mathscr{T}}}_{n,m,p}$. Although we consider tensors of fixed size (${{\mathscr{T}}}_{n,m,p}$ has size *nm* × *mp* × *pn*), the discovered algorithms can be applied recursively to multiply matrices of arbitrary size. We use AlphaTensor to find matrix multiplication algorithms over different arithmetics—namely, modular arithmetic (that is, multiplying matrices in the quotient ring ${{\mathbb{Z}}}_{2}$), and standard arithmetic (that is, multiplying matrices in ${\mathbb{R}}$).

**Fig. 3:**
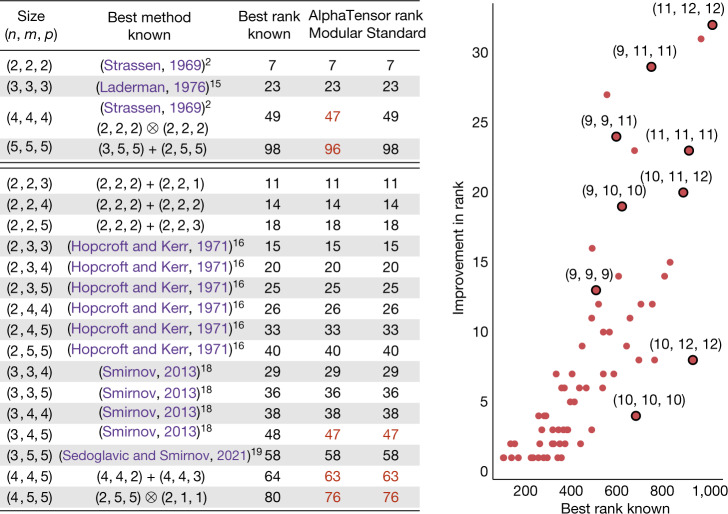
Comparison between the complexity of previously known matrix multiplication algorithms and the ones discovered by AlphaTensor. Left: column (*n*, *m*, *p*) refers to the problem of multiplying *n* × *m* with *m* × *p* matrices. The complexity is measured by the number of scalar multiplications (or equivalently, the number of terms in the decomposition of the tensor). ‘Best rank known’ refers to the best known upper bound on the tensor rank (before this paper), whereas ‘AlphaTensor rank’ reports the rank upper bounds obtained with our method, in modular arithmetic (${{\mathbb{Z}}}_{2}$) and standard arithmetic. In all cases, AlphaTensor discovers algorithms that match or improve over known state of the art (improvements are shown in red). See Extended Data Figs. 1 and 2 for examples of algorithms found with AlphaTensor. Right: results (for arithmetic in ${\mathbb{R}}$) of applying AlphaTensor-discovered algorithms on larger tensors. Each red dot represents a tensor size, with a subset of them labelled. See Extended Data Table 1 for the results in table form. State-of-the-art results are obtained from the list in ref. ^64^.

**Extended Data Fig. 1:**
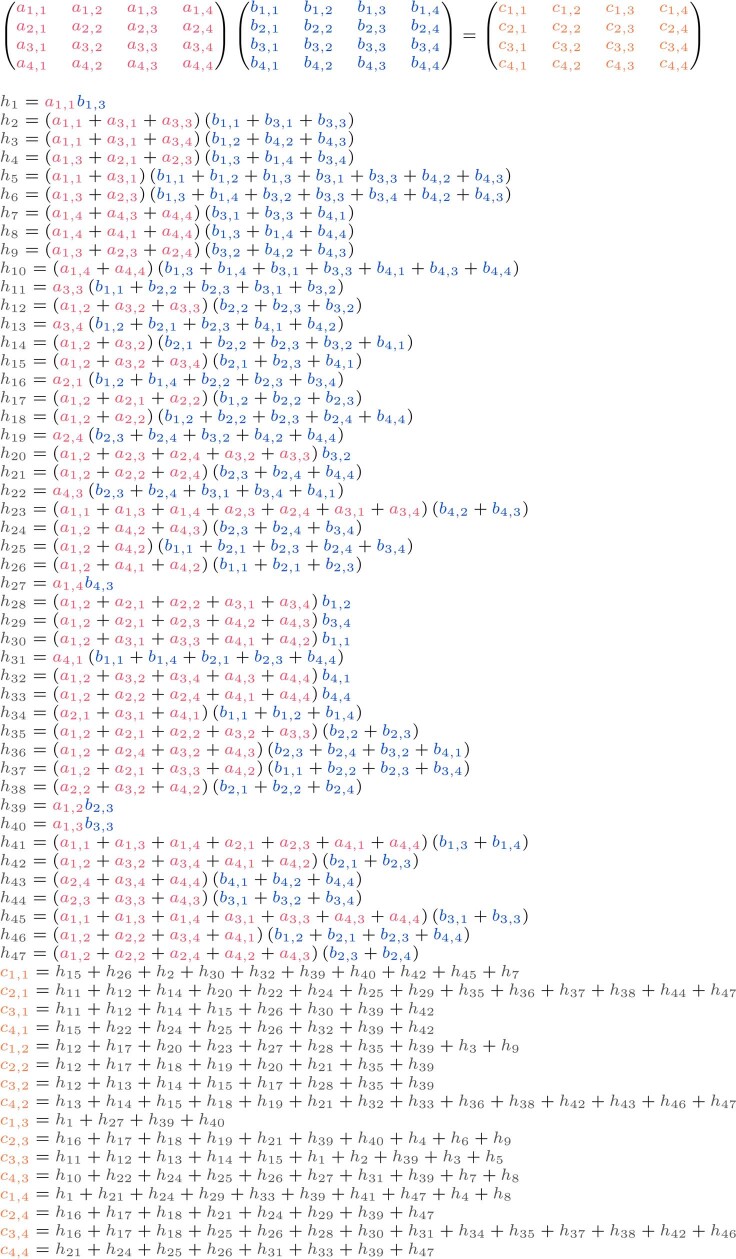
Algorithm for multiplying 4 × 4 matrices in modular arithmetic (${{\mathbb{Z}}}_{2}$) with 47 multiplications. This outperforms the two-level Strassen’s algorithm, which involves 7^2^ = 49 multiplications.

**Extended Data Fig. 2:**
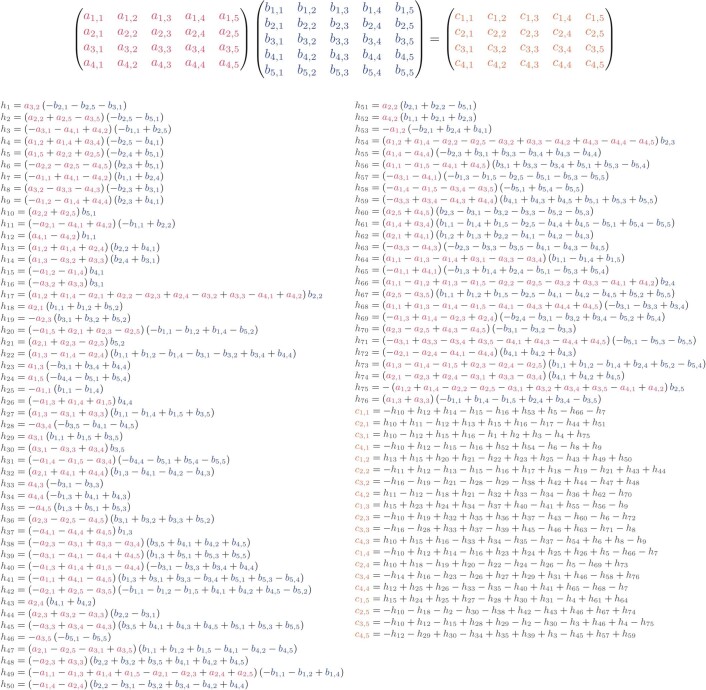
Algorithm for multiplying 4 × 5 by 5 × 5 matrices in standard arithmetic with 76 multiplications. This outperforms the previously best known algorithm, which involves 80 multiplications.

Figure 3 (left) shows the complexity (that is, rank) of the algorithms discovered by AlphaTensor. AlphaTensor re-discovers the best algorithms known for multiplying matrices (for example, Strassen’s^2^ and Laderman’s^15^ algorithms). More importantly, AlphaTensor improves over the best algorithms known for several matrix sizes. In particular, AlphaTensor finds an algorithm for multiplying 4 × 4 matrices using 47 multiplications in ${{\mathbb{Z}}}_{2}$, thereby outperforming Strassen’s two-level algorithm^2^, which involves 7^2^ = 49 multiplications. By applying this algorithm recursively, one obtains a practical matrix multiplication algorithm in ${{\mathbb{Z}}}_{2}$ with complexity ${\mathcal{O}}({N}^{2.778})$. Moreover, AlphaTensor discovers efficient algorithms for multiplying matrices in standard arithmetic; for example, AlphaTensor finds a rank-76 decomposition of ${{\mathscr{T}}}_{4,5,5}$, improving over the previous state-of-the-art complexity of 80 multiplications. See Extended Data Figs. 1 and 2 for examples.

AlphaTensor generates a large database of matrix multiplication algorithms—up to thousands of algorithms for each size. We exploit this rich space of algorithms by combining them recursively, with the aim of decomposing larger matrix multiplication tensors. We refer to refs. ^25,26^ and Appendix H in Supplementary Information for more details. Using this approach, we improve over the state-of-the-art results for more than 70 matrix multiplication tensors (with *n*, *m*, *p* ≤ 12). See Fig. 3 (right) and Extended Data Table 1 for the results.

A crucial aspect of AlphaTensor is its ability to learn to transfer knowledge between targets (despite providing no prior knowledge on their relationship). By training one agent to decompose various tensors, AlphaTensor shares learned strategies among these, thereby improving the overall performance (see Supplementary Information for analysis). Finally, it is noted that AlphaTensor scales beyond current computational approaches for decomposing tensors. For example, to our knowledge, no previous approach was able to handle ${{\mathscr{T}}}_{4}$, which has an action space 10^10^ times larger than ${{\mathscr{T}}}_{3}$. Our agent goes beyond this limit, discovering decompositions matching or surpassing state-of-the-art for large tensors such as ${{\mathscr{T}}}_{5}$.

### Analysing the symmetries of matrix multiplication algorithms

From a mathematical standpoint, the diverse algorithms discovered by AlphaTensor show that the space is richer than previously known. For example, while the only known rank-49 factorization decomposing ${{\mathscr{T}}}_{4}={{\mathscr{T}}}_{2}\otimes {{\mathscr{T}}}_{2}$ before this paper conforms to the product structure (that is, it uses the factorization of ${{\mathscr{T}}}_{2}$ twice, which we refer to as Strassen-square^2^), AlphaTensor finds more than 14,000 non-equivalent factorizations (with standard arithmetic) that depart from this scheme, and have different properties (such as matrix ranks and sparsity—see Supplementary Information). By non-equivalent, we mean that it is not possible to obtain one from another by applying a symmetry transformation (such as permuting the factors). Such properties of matrix multiplication tensors are of great interest, as these tensors represent fundamental objects in algebraic complexity theory^3,5,7^. The study of matrix multiplication symmetries can also provide insight into the asymptotic complexity of matrix multiplication^5^. By exploring this rich space of algorithms, we believe that AlphaTensor will be useful for generating results and guiding mathematical research. See Supplementary Information for proofs and details on the symmetries of factorizations.

### Beyond standard matrix multiplication

**Fig. 4:**
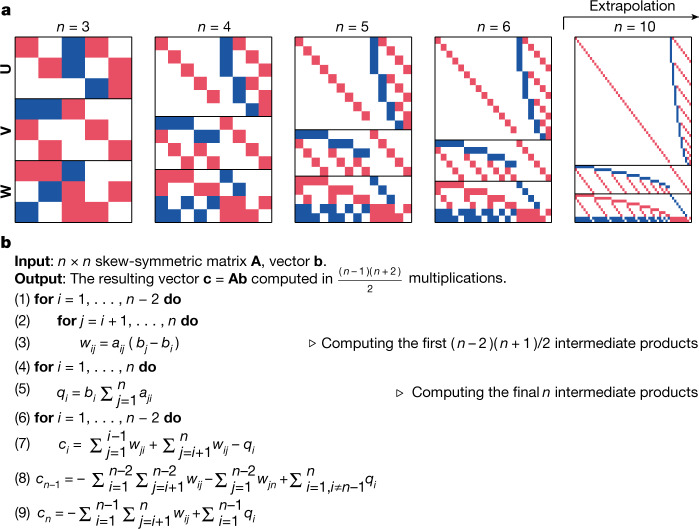
Algorithm discovery beyond standard matrix multiplication. **a**, Decompositions found by AlphaTensor for the tensors of size $\frac{n(n-1)}{2}\times n\times n$ (with *n* = 3, 4, 5, 6) representing the skew-symmetric matrix-vector multiplication. The red pixels denote 1, the blue pixels denote −1 and the white pixels denote 0. Extrapolation to *n* = 10 is shown in the rightmost figure. **b**, Skew-symmetric matrix-by-vector multiplication algorithm, obtained from the examples solved by AlphaTensor. The *w*_*ij*_ and *q*_*i*_ terms in steps 3 and 5 correspond to the *m*_*r*_ terms in Algorithm 1. It is noted that steps 6–9 do not involve any multiplications.

Tensors can represent any bilinear operation, such as structured matrix multiplication, polynomial multiplication or more custom bilinear operations used in machine learning^27,28^. We demonstrate here a use-case where AlphaTensor finds a state-of-the-art algorithm for multiplying an *n* x *n* skew-symmetric matrix  with a vector of length *n*. Figure 4a shows the obtained decompositions for small instance sizes *n*. We observe a pattern that we generalize to arbitrary *n*, and prove that this yields a general algorithm for the skew-symmetric matrix-vector product (Fig. 4b). This algorithm, which uses $(n-1)(n+2)/2 \sim \frac{1}{2}{n}^{2}$ multiplications (where ∼ indicates asymptotic similarity), outperforms the previously known algorithms using  asymptotically *n*^2^ multiplications^29^, and is asymptotically optimal. See Supplementary Information for a proof, and for another use-case showing AlphaTensor’s ability to re-discover the Fourier basis (see also Extended Data Table 2). This shows that AlphaTensor can be applied to custom bilinear operations, and yield efficient algorithms leveraging the problem structure.

### Rapid tailored algorithm discovery

We show a use-case where AlphaTensor finds practically efficient matrix multiplication algorithms, tailored to specific hardware, with zero prior hardware knowledge. To do so, we modify the reward of AlphaTensor: we provide an additional reward at the terminal state (after the agent found a correct algorithm) equal to the negative of the runtime of the algorithm when benchmarked on the target hardware. That is, we set ${r}_{t}^{{\prime} }={r}_{t}+\lambda {b}_{t}$, where *r*_*t*_ is the reward scheme described in ‘DRL for algorithm discovery’, *b*_*t*_ is the benchmarking reward (non-zero only at the terminal state) and *λ* is a user-specified coefficient. Aside from the different reward, the exact same formulation of TensorGame is used.

We train AlphaTensor to search for efficient algorithms to multiply 4 × 4 block matrices, and focus on square matrix multiplication of size 8,192 (each block is hence of size 2,048) to define the benchmarking reward. AlphaTensor searches for the optimal way of combining the 16 square blocks of the input matrices on the considered hardware. We do not apply the 4 × 4 algorithm recursively, to leverage the efficient implementation of matrix multiplication on moderate-size matrices (2,048 × 2,048 in this case). We study two hardware devices commonly used in machine learning and scientific computing: an Nvidia V100 graphics processing unit (GPU) and a Google tensor processing unit (TPU) v2. The factorization obtained by AlphaTensor is transformed into JAX^30^ code, which is compiled (just in time) before benchmarking.

**Fig. 5:**
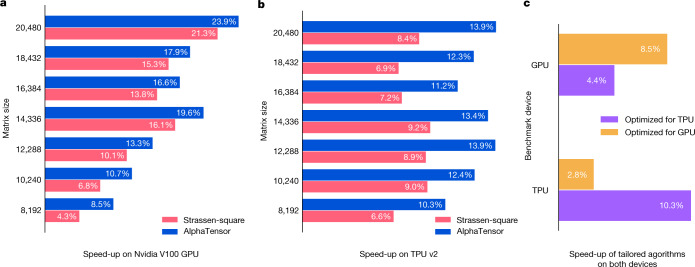
Speed-ups of the AlphaTensor-discovered algorithm. **a**,**b**, Speed-ups (%) of the AlphaTensor-discovered algorithms tailored for a GPU (**a**) and a TPU (**b**), optimized for a matrix multiplication of size 8,192 × 8,192. Speed-ups are measured relative to standard (for example, cuBLAS for the GPU) matrix multiplication on the same hardware. Speed-ups are reported for various matrix sizes (despite optimizing the algorithm only on one matrix size). We also report the speed-up of the Strassen-square  algorithm. The median speed-up is reported over 200 runs. The standard deviation over runs is <0.4 percentage points (see Supplementary Information for more details). **c**, Speed-up of both algorithms (tailored to a GPU and a TPU) benchmarked on both devices.

Figure 5a,b shows the efficiency of the AlphaTensor-discovered algorithms on the GPU and the TPU, respectively. AlphaTensor discovers algorithms that outperform the Strassen-square algorithm, which is a fast algorithm for large square matrices^31,32^. Although the discovered algorithm has the same theoretical complexity as Strassen-square, it outperforms it in practice, as it is optimized for the considered hardware. Interestingly, AlphaTensor finds algorithms with a larger number of additions compared with Strassen-square (or equivalently, denser decompositions), but the discovered algorithms generate individual operations that can be efficiently fused by the specific XLA^33^ grouping procedure and thus are more tailored towards the compiler stack we use. The algorithms found by AlphaTensor also provide gains on matrix sizes larger than what they were optimized for. Finally, Fig. 5c shows the importance of tailoring to particular hardware, as algorithms optimized for one hardware do not perform as well on other hardware.

## Discussion

Trained from scratch, AlphaTensor discovers matrix multiplication algorithms that are more efficient than existing human and computer-designed algorithms. Despite improving over known algorithms, we note that a limitation of AlphaTensor is the need to pre-define a set of potential factor entries *F*, which discretizes the search space but can possibly lead to missing out on efficient algorithms. An interesting direction for future research is to adapt AlphaTensor to search for *F*. One important strength of AlphaTensor is its flexibility to support complex stochastic and non-differentiable rewards (from the tensor rank to practical efficiency on specific hardware), in addition to finding algorithms for custom operations in a wide variety of spaces (such as finite fields). We believe this will spur applications of AlphaTensor towards designing algorithms that optimize metrics that we did not consider here, such as numerical stability or energy usage.

The discovery of matrix multiplication algorithms has far-reaching implications, as matrix multiplication sits at the core of many computational tasks, such as matrix inversion, computing the determinant and solving linear systems, to name a few^7^. We also note that our methodology can be extended to tackle related primitive mathematical problems, such as computing other notions of rank (for example, border rank—see Supplementary Information), and NP-hard matrix factorization problems (for example, non-negative factorization). By tackling a core NP-hard computational problem in mathematics using DRL—the computation of tensor ranks—AlphaTensor demonstrates the viability of DRL in addressing difficult mathematical problems, and potentially assisting mathematicians in discoveries.

## Methods

### TensorGame

TensorGame is played as follows. The start position ${{\mathscr{S}}}_{0}$ of the game corresponds to the tensor ${\mathscr{T}}$ representing the bilinear operation of interest, expressed in some basis. In each step *t* of the game, the player writes down three vectors (**u**^(*t*)^, **v**^(*t*)^, **w**^(*t*)^), which specify the rank-1 tensor **u**^(*t*)^ ⊗ **v**^(*t*)^ ⊗ **w**^(*t*)^, and the state of the game is updated by subtracting the newly written down factor:

2 $${{\mathscr{S}}}_{t}\leftarrow {{\mathscr{S}}}_{t-1}-{{\bf{u}}}^{(t)}\otimes {{\bf{v}}}^{(t)}\otimes {{\bf{w}}}^{(t)}.$$

The game ends when the state reaches the zero tensor, ${{\mathscr{S}}}_{R}={\bf{0}}$. This means that the factors written down throughout the game form a factorization of the start tensor ${{\mathscr{S}}}_{0}$, that is, ${{\mathscr{S}}}_{0}={\sum }_{t=1}^{R}{{\bf{u}}}^{(t)}\otimes {{\bf{v}}}^{(t)}\otimes {{\bf{w}}}^{(t)}$. This factorization is then scored. For example, when optimizing for asymptotic time complexity the score is −*R*, and when optimizing for practical runtime the algorithm corresponding to the factorization ${\{({{\bf{u}}}^{(t)},{{\bf{v}}}^{(t)},{{\bf{w}}}^{(t)})\}}_{t=1}^{R}$ is constructed (see Algorithm 1) and then benchmarked on the fly (see Supplementary Information).

In practice, we also impose a limit *R*_limit_ on the maximum number of moves in the game, so that a weak player is not stuck in unnecessarily (or even infinitely) long games. When a game ends  because it has run out of moves, a penalty score is given so that it is never advantageous to deliberately exhaust the move limit. For example, when optimizing for asymptotic time complexity, this penalty is derived from an upper bound on the tensor rank of the final residual tensor ${{\mathscr{S}}}_{{R}_{\text{limit}}}$. This upper bound on the tensor rank is obtained by summing the matrix ranks of the slices of the tensor.

#### TensorGame over rings

We say that the decomposition of ${{\mathscr{T}}}_{n}$ in equation (1) is in a ring ${\mathcal{E}}$ (defining the arithmetic operations) if each of the factors **u**^(*t*)^, **v**^(*t*)^ and **w**^(*t*)^ has entries belonging to the set ${\mathcal{E}}$, and additions and multiplications are interpreted according to ${\mathcal{E}}$. The tensor rank depends, in general, on the ring. At each step of TensorGame, the additions and multiplications in equation (2) are interpreted in ${\mathcal{E}}$. For example, when working in ${{\mathbb{Z}}}_{2}$, (in this case, the factors **u**^(*t*)^, **v**^(*t*)^ and **w**^(*t*)^ live in *F* = {0, 1}), a modulo 2 operation is applied after each state update (equation (2)).

We note that integer-valued decompositions **u**^(*t*)^, **v**^(*t*)^ and **w**^(*t*)^ lead to decompositions in arbitrary rings ${\mathcal{E}}$. Hence, provided *F* only contains integers, algorithms we find in standard arithmetic apply more generally to any ring.

### AlphaTensor

AlphaTensor builds on AlphaZero^1^ and its extension Sampled AlphaZero^21^, combining a deep neural network with a sample-based MCTS search algorithm.

The deep neural network, *f*_*θ*_(*s*) = (*π*, *z*) parameterized by *θ*, takes as input the current state *s* of the game and outputs a probability distribution *π*(⋅∣*s*) over actions and *z*(⋅∣*s*) over returns (sum of future rewards) *G*. The parameters *θ* of the deep neural network are trained by reinforcement learning from self-play games and synthetic demonstrations. Self-play games are played by actors, running a sample-based MCTS search at every state *s*_*t*_ encountered in the game. The MCTS search returns an improved probability distribution over moves from which an action *a*_*t*_ is selected and applied to the environment. The sub-tree under *a*_*t*_ is reused for the subsequent search at *s*_*t*+1_. At the end of the game, a return *G* is obtained and the trajectory is sent to the learner to update the neural network parameters *θ*. The distribution over returns *z*(⋅∣*s*_*t*_) is learned through distributional reinforcement learning using the quantile regression distributional loss^34^, and the network policy *π*(⋅∣*s*_*t*_) is updated using a Kullback–Leibler divergence loss, to maximize its similarity to the search policy for self-play games or to the next action for synthetic demonstrations. We use the Adam optimizer^35^ with decoupled weight decay^36^ to optimize the parameters *θ* of the neural network.

#### Sample-based MCTS search

The sample-based MCTS search is very similar to the one described in Sampled AlphaZero. Specifically, the search consists of a series of simulated trajectories of TensorGame that are aggregated in a tree. The search tree therefore consists of nodes representing states and edges representing actions. Each state-action pair (*s*, *a*) stores a set of statistics $N(s,a),Q(s,a),\hat{\pi }(s,a)$, where *N*(*s*, *a*) is the visit count, *Q*(*s*, *a*) is the action value and $\hat{\pi }(s,a)$ is the empirical policy probability. Each simulation traverses the tree from the root state *s*_0_ until a leaf state *s*_L_ is reached by recursively selecting in each state *s* an action *a* that has not been frequently explored, has high empirical policy probability and high value. Concretely, actions within the tree are selected by maximizing over the probabilistic upper confidence tree bound^21,37^

$$\mathop{{\rm{argmax}}}\limits_{a}Q(s,a)+c(s)\cdot \hat{\pi }(s,a)\frac{\sqrt{{\sum }_{b}N(s,b)}}{1+N(s,a)},$$

where *c*(*s*) is an exploration factor controlling the influence of the empirical policy $\hat{\pi }(s,a)$ relative to the values *Q*(*s*, *a*) as nodes are visited more often. In addition, a transposition table is used to recombine different action sequences if they reach the exact same tensor. This can happen particularly often in TensorGame as actions are commutative. Finally, when a leaf state *s*_L_ is reached, it is evaluated by the neural network, which returns *K* actions {*a*_*i*_} sampled from *π*(*a*∣*s*_L_), alongside the empirical distribution $\hat{\pi }(a| {s}_{{\rm{L}}})=\frac{1}{K}{\sum }_{i}{\delta }_{a,{a}_{i}}$ and a value *v*(*s*_L_) constructed from *z*(⋅∣*s*_L_). Differently from AlphaZero and Sampled AlphaZero, we chose *v* not to be the mean of the distribution of returns *z*(⋅∣*s*_*L*_) as is usual in most reinforcement learning agents, but instead to be a risk-seeking value, leveraging the facts that TensorGame is a deterministic environment and that we are primarily interested in finding the best trajectory possible. The visit counts and values on the simulated trajectory are then updated in a backward pass as in Sampled AlphaZero.

#### Policy improvement

After simulating *N*(*s*) trajectories from state *s* using MCTS, the normalized visit counts of the actions at the root of the search tree *N*(*s*, *a*)/*N*(*s*) form a sample-based improved policy. Differently from AlphaZero and Sampled AlphaZero, we use an adaptive temperature scheme to smooth the normalized visit counts distribution as some states can accumulate an order of magnitude more visits than others because of sub-tree reuse and transposition table. Concretely, we define the improved policy as ${\mathcal{I}}\hat{\pi }(s,a)={N}^{1/\tau (s)}(s,a)/{\sum }_{b}{N}^{1/\tau (s)}(s,b)$ where $\tau (s)=\log N(s)/\log \bar{N}\,{\rm{if}}\,N > \bar{N}$ and 1 otherwise, with $\bar{N}$ being a hyperparameter. For training, we use ${\mathcal{I}}\hat{\pi }$ directly as a target for the network policy *π*. For acting, we additionally discard all actions that have a value lower than the value of the most visited action, and sample proportionally to ${\mathcal{I}}\hat{\pi }$ among those remaining high-value actions.

#### Learning one agent for multiple target tensors

We train a single agent to decompose the different tensors ${{\mathscr{T}}}_{n,m,p}$ in a given arithmetic (standard or modular). As the network works with fixed-size inputs, we pad all tensors (with zeros) to the size of the largest tensor we consider (${{\mathscr{T}}}_{5}$, of size 25 × 25 × 25). At the beginning of each game, we sample uniformly at random a target ${{\mathscr{T}}}_{n,m,p}$, and play TensorGame. Training a single agent on different targets leads to better results thanks to the transfer between targets. All our results reported in Fig. 3 are obtained using multiple runs of this multi-target setting. We also train a single agent to decompose tensors in both arithmetics. Owing to learned transfer between the two arithmetics, this agent discovers a different distribution of algorithms (of the same ranks) in standard arithmetic than the agent trained on standard arithmetic only, thereby increasing the overall diversity of discovered algorithms.

#### Synthetic demonstrations

The synthetic demonstrations buffer contains tensor-factorization pairs, where the factorizations ${\{({{\bf{u}}}^{(r)},{{\bf{v}}}^{(r)},{{\bf{w}}}^{(r)})\}}_{r=1}^{R}$ are first generated at random, after which the tensor ${\mathscr{D}}={\sum }_{r=1}^{R}{{\bf{u}}}^{(r)}\otimes {{\bf{v}}}^{(r)}\otimes {{\bf{w}}}^{(r)}$ is formed. We create a dataset containing 5 million such tensor-factorization pairs. Each element in the factors is sampled independently and identically distributed (i.i.d.) from a given categorical distribution over *F* (all possible values that can be taken). We discarded instances whose decompositions were clearly suboptimal (contained a factor with **u** = **0**, **v** = **0**, or **w** = **0**).

In addition to these synthetic demonstrations, we further add to the demonstration buffer previous games that have achieved large scores to reinforce the good moves made by the agent in these games.

#### Change of basis

The rank of a bilinear operation does not depend on the basis in which the tensor representing it is expressed, and for any invertible matrices **A**, **B** and **C** we have ${\rm{Rank}}\,({\mathscr{T}})={\rm{Rank}}\,({{\mathscr{T}}}^{({\bf{A}},{\bf{B}},{\bf{C}})})$, where ${{\mathscr{T}}}^{({\bf{A}},{\bf{B}},{\bf{C}})}$ is the tensor after change of basis given by

3 $${{\mathscr{T}}}_{ijk}^{({\bf{A}},{\bf{B}},{\bf{C}})}=\mathop{\sum }\limits_{a=1}^{S}\mathop{\sum }\limits_{b=1}^{S}\mathop{\sum }\limits_{c=1}^{S}{{\bf{A}}}_{ia}{{\bf{B}}}_{jb}{{\bf{C}}}_{kc}{{\mathscr{T}}}_{abc}.$$

Hence, exhibiting a rank-*R* decomposition of the matrix multiplication tensor ${{\mathscr{T}}}_{n}$ expressed in any basis proves that the product of two *n* × *n* matrices can be computed using *R* scalar multiplications. Moreover, it is straightforward to convert such a rank-*R* decomposition into a rank-*R* decomposition in the canonical basis, thus yielding a practical algorithm of the form shown in Algorithm 1. We leverage this observation by expressing the matrix multiplication tensor ${{\mathscr{T}}}_{n}$ in a large number of randomly generated bases (typically 100,000) in addition to the canonical basis, and letting AlphaTensor play games in all bases in parallel.

This approach has three appealing properties: (1) it provides a natural exploration mechanism as playing games in different bases automatically injects diversity into the games played by the agent; (2) it exploits properties of the problem as the agent need not succeed in all bases—it is sufficient to find a low-rank decomposition in any of the bases; (3) it enlarges coverage of the algorithm space because a decomposition with entries in a finite set *F* = {−2, −1, 0, 1, 2} found in a different basis need not have entries in the same set when converted back into the canonical basis.

In full generality, a basis change for a 3D tensor of size *S* × *S* × *S* is specified by three invertible *S* × *S* matrices **A**, **B** and **C**. However, in our procedure, we sample bases at random and impose two restrictions: (1) **A** = **B** = **C**, as this performed better in early experiments, and (2) unimodularity ($\det {\bf{A}}\in \{-1,+1\}$), which ensures that after converting an integral factorization into the canonical basis it still contains integer entries only (this is for representational convenience and numerical stability of the resulting algorithm). See Supplementary Information for the exact algorithm.

#### Signed permutations

In addition to playing (and training on) games in different bases, we also utilize a data augmentation mechanism whenever the neural network is queried in a new MCTS node. At acting time, when the network is queried, we transform the input tensor by applying a change of basis—where the change of basis matrix is set to a random signed permutation. We then query the network on this transformed input tensor, and finally invert the transformation in the network’s policy predictions. Although this data augmentation procedure can be applied with any generic change of basis matrix (that is, it is not restricted to signed permutation matrices), we use signed permutations mainly for computational efficiency. At training time, whenever the neural network is trained on an (input, policy targets, value target) triplet (Fig. 2), we apply a randomly chosen signed permutation to both the input and the policy targets, and train the network on this transformed triplet. In practice, we sample 100 signed permutations at the beginning of an experiment, and use them thereafter.

#### Action canonicalization

For any *λ*_1_, *λ*_2_, *λ*_3_ ∈ {−1, +1} such that *λ*_1_*λ*_2_*λ*_3_ = 1, the actions (*λ*_1_**u**, *λ*_2_**v**, *λ*_3_**w**) and (**u**, **v**, **w**) are equivalent because they lead to the same rank-one tensor (*λ*_1_**u**) ⊗ (*λ*_2_**v**) ⊗ (*λ*_3_**w**) = **u** ⊗ **v** ⊗ **w**. To prevent the network from wasting capacity on predicting multiple equivalent actions, during training we always present targets (**u**, **v**, **w**) for the policy head in a canonical form, defined as having the first non-zero element of **u** and the first non-zero element of **v** strictly positive. This is well defined because **u** or **v** cannot be all zeros (if they are to be part of a minimal rank decomposition), and for any (**u**, **v**, **w**) there are unique *λ*_1_, *λ*_2_, *λ*_3_ ∈ {−1, +1} (with *λ*_1_*λ*_2_*λ*_3_ = 1) that transform it into canonical form. In case the network predicts multiple equivalent actions anyway, we merge them together (summing their empirical policy probabilities) before inserting them into the MCTS tree.

#### Training regime

We train AlphaTensor on a TPU v3, with a total batch size of 2,048. We use 64 TPU cores, and train for 600,000 iterations. On the actor side, the games are played on standalone TPU v4, and we use 1,600 actors. In practice, the procedure  takes a week to converge.

### Neural network

**Extended Data Fig. 3:**
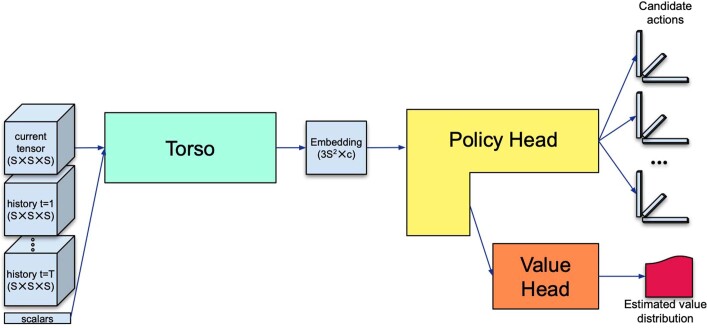
AlphaTensor’s network architecture. The network takes as input the list of tensors containing the current state and previous history of actions, and a list of scalars, such as the time index of the current action. It produces two kinds of outputs: one representing the value, and the other inducing a distribution over the action space from which we can sample from. The architecture of the network is accordingly designed to have a common torso, and two heads, the value and the policy heads. *c* is set to 512 in all experiments.

The architecture is composed of a torso, followed by a policy head that predicts a distribution over actions, and a value head that predicts a distribution of the returns from the current state (see Extended Data Fig. 3).

#### Input

The input to the network contains all the relevant information of the current state and is composed of a list of tensors and a list of scalars. The most important piece of information is the current 3D tensor ${{\mathscr{S}}}_{t}$ of size *S* × *S* × *S*. (For simplicity, in the description here we assume that all the three dimensions of the tensor are equal in size. The generalization to different sizes is straightforward.) In addition, the model is given access to the last *h* actions (*h* being a hyperparameter usually set to 7), represented as *h* rank-1 tensors that are concatenated to the input. The list of scalars includes the time index *t* of the current action (where 0 ≤ *t* < *R*_limit_).

#### Torso

The torso of the network is in charge of mapping both scalars and tensors from the input to a representation that is useful to both policy and value heads. Its architecture is based on a modification of transformers^23^, and its main signature is that it operates over three *S* × *S* grids projected from the *S* × *S* × *S* input tensors. Each grid represents two out of the three modes of the tensor. Defining the modes of the tensor as ${\mathcal{U}},{\mathcal{V}},{\mathcal{W}}$, the rows and columns of the first grid are associated to ${\mathcal{U}}$ and ${\mathcal{V}}$, respectively, the rows and columns of the second grid are associated to ${\mathcal{W}}$ and ${\mathcal{U}}$, and the rows and columns of the third grid are associated to ${\mathcal{V}}$ and ${\mathcal{W}}$. Each element of each grid is a feature vector, and its initial value is given by the elements of the input tensors along the grid’s missing mode. These feature vectors are enriched by concatenating an *S* × *S* × 1 linear projection from the scalars. This is followed by a linear layer projecting these feature vectors into a 512-dimensional space.

**Extended Data Fig. 4:**
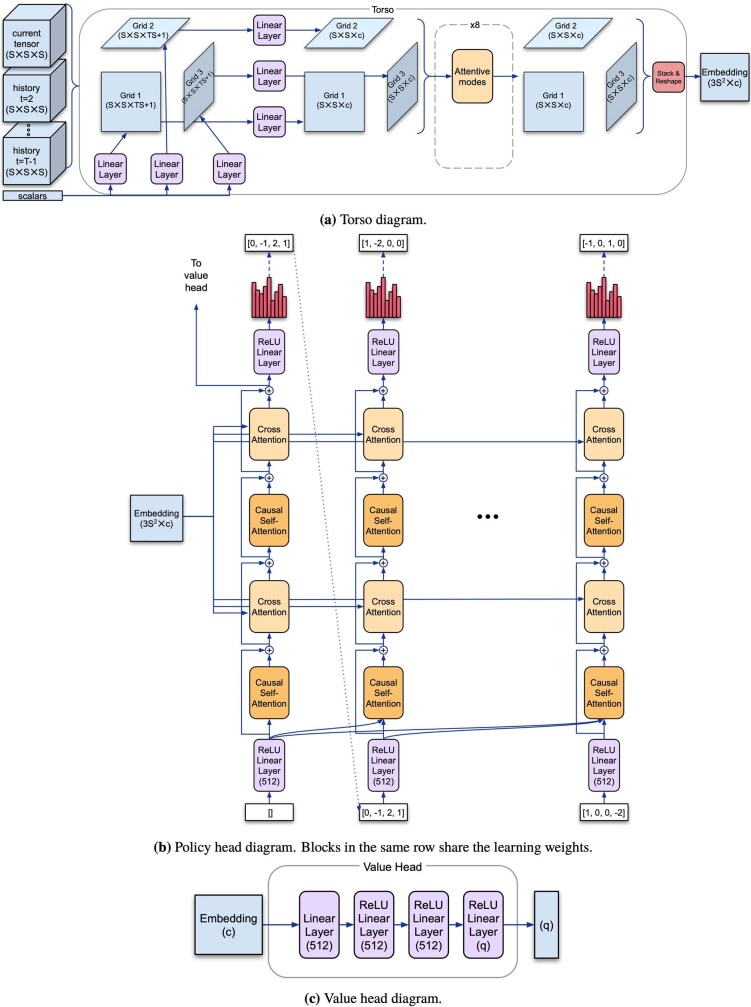
Detailed view of AlphaTensor’s architecture, included torso, policy and value head. We refer to Algorithms A.1-A.11 in Supplementary Information for the details of each component.

The rest of the torso is a sequence of attention-based blocks with the objective of propagating information between the three grids. Each of those blocks has three stages, one for every pair of grids. In each stage, the grids involved are concatenated, and axial attention^24^ is performed over the columns. It is noted that in each stage we perform in parallel *S* self-attention operations of 2*S* elements in each. The representation sent to the policy head corresponds to the 3*S*^2 ^512-dimensional feature vectors produced by the last layer of the torso. A detailed description of the structure of the torso is specified in Extended Data Fig. 4 (top) and Appendix A.1.1 in Supplementary Information.

#### Policy head

The policy head uses the transformer architecture^23^ to model an autoregressive policy. Factors are decomposed into *k* tokens of dimensionality *d* such that *k* × *d* = 3*S*. The transformer conditions on the tokens already generated and cross-attends to the features produced by the torso. At training time, we use teacher-forcing, that is, the ground truth actions are decomposed into tokens and taken as inputs into the causal transformer in such a way that the prediction of a token depends only on the previous tokens. At inference time, *K* actions are sampled from the head. The feature representation before the last linear layer of the initial step (that is, the only step that is not conditioned on the ground truth) is used as an input to the value head, described below. Details of the architecture are presented in Extended Data Fig. 4 (centre) and Appendix A.1.2 in Supplementary Information.

#### Value head

The value head is composed of a four-layer multilayer perceptron whose last layer produces *q* outputs corresponding to the $\frac{1}{2q},\frac{3}{2q},\ldots \frac{2q-1}{2q}$ quantiles. In this way, the value head predicts the distribution of returns from this state in the form of values predicted for the aforementioned quantiles^34^. At inference time, we encourage the agent to be risk-seeking by using the average of the predicted values for quantiles over 75%. A detailed description of the value head is presented in Extended Data Fig. 4 (bottom) and Appendix A.1.3 in Supplementary Information.

### Related work

The quest for efficient matrix multiplication algorithms started with Strassen’s breakthrough in ref. ^2^, which showed that one can multiply 2 × 2 matrices using 7 scalar multiplications, leading to an algorithm of complexity ${\mathcal{O}}({n}^{2.81})$. This led to the development of a very active field of mathematics attracting worldwide interest, which studies the asymptotic complexity of matrix multiplication (see refs. ^3–6^). So far, the best known complexity for matrix multiplication is ${\mathcal{O}}({n}^{2.37286})$ (ref. ^12^), which improves over ref. ^11^, and builds on top of fundamental results in the field^8–10^. However, this does not yield practical algorithms, as such approaches become advantageous only for astronomical matrix sizes. Hence, a significant body of work aims at exhibiting explicit factorizations of matrix multiplication tensors, as these factorizations provide practical algorithms. After Strassen’s breakthrough showing that $\text{rank}\,({{\mathscr{T}}}_{2})\le 7$, efficient algorithms for larger matrix sizes were found^15,16,18,26,38^. Most notably, Laderman showed in ref. ^15^ that 3 × 3 matrix multiplications can be performed with 23 scalar multiplications. In addition to providing individual low-rank factorizations, an important research direction aims at understanding the space of matrix multiplication algorithms—as opposed to exhibiting individual low-rank factorizations—by studying the symmetry groups and diversity of factorizations (see ref. ^5^ and references therein). For example, the symmetries of 2 × 2 matrix multiplication were studied in refs. ^39–42^, where Strassen’s algorithm was shown to be essentially unique. The case of 3 × 3 was studied in ref. ^43^, whereas a symmetric factorization for all *n* is provided in ref. ^44^.

On the computational front, continuous optimization has been the main workhorse for decomposing tensors^17,45,46^, and in particular matrix multiplication tensors. Such continuous optimization procedures (for example, alternating least squares), however, yield approximate solutions, which correspond to inexact matrix multiplication algorithms with floating point operations. To circumvent this issue, regularization procedures have been proposed, such as ref. ^18^, to extract exact decompositions. Unfortunately, such approaches often require  substantial human intervention and expertise to decompose large tensors. A different line of attack was explored in refs. ^47,48^, based on learning the continuous weights of a two-layer network that mimics the structure of the matrix multiplication operation. This method, which is trained through supervised learning of matrix multiplication examples, finds approximate solutions to 2 × 2 and 3 × 3 matrix multiplications. In ref. ^48^, a quantization procedure is further used to obtain an exact decomposition for 2 × 2. Unlike continuous optimization-based approaches, AlphaTensor directly produces algorithms from the desired set of valid algorithms, and is flexible in that it allows us to optimize a wide range of (even non-differentiable) objectives. This unlocks tackling broader settings (for example, optimization in finite fields, optimization of runtime), as well as larger problems (for example, ${{\mathscr{T}}}_{4}$ and ${{\mathscr{T}}}_{5}$) than those previously considered. Different from continuous optimization, a boolean satisfiability (SAT) based  formulation of the problem of decomposing 3 × 3 matrix multiplication was recently proposed in ref. ^20^, which adds thousands of new decompositions of rank 23 to the list of known 3 × 3 factorizations. The approach relies on a state-of-the-art SAT solving procedure, where several assumptions and simplifications are made on the factorizations to reduce the search space. As is, this approach is, however, unlikely to scale to larger tensors, as the search space grows very quickly with the size.

On the practical implementation front, ref. ^31^ proposed several ideas to speed up implementation of fast matrix multiplication algorithms on central processing units (CPUs). Different fast algorithms are then compared and benchmarked, and the potential speed-up of such algorithms is shown against standard multiplication. Other works focused on getting the maximal performance out of a particular fast matrix multiplication algorithm (Strassen’s algorithm with one or two levels of recursion) on a CPU^32^ or a GPU^49^. These works show that, despite popular belief, such algorithms are of practical value. We see writing a custom low-level implementation of a given algorithm to be distinct from the focus of this paper—developing new efficient algorithms—and we believe that the algorithms we discovered can further benefit from a more efficient implementation by experts.

Beyond matrix multiplication and bilinear operations, a growing amount of research studies the use of optimization and machine learning to improve the efficiency of computational operations. There are three levels of abstractions at which this can be done: (1) in the hardware design, for example, chip floor planning^50^, (2) at the hardware–software interface, for example, program super-optimization of a reference implementation for specific hardware^51^, and (3) on the algorithmic level, for example, program induction^52^, algorithm selection^53^ or meta-learning^54^. Our work focuses on the algorithmic level of abstraction, although AlphaTensor is also flexible to discover efficient algorithms for specific hardware. Different from previous works, we focus on discovering matrix multiplication algorithms that are provably correct, without requiring initial reference implementations. We conclude by relating our work broadly to existing reinforcement learning methods for scientific discovery. Within mathematics, reinforcement learning was applied, for example, to theorem proving^55–58^, and to finding counterexamples refuting conjectures in combinatorics and graph theory^59^. Reinforcement learning was further shown to be useful in many areas in science, such as molecular design^60,61^ and synthesis^62^ and optimizing quantum dynamics^63^.

## Online content

Any methods, additional references, Nature Research reporting summaries, source data, extended data, supplementary information, acknowledgements, peer review information; details of author contributions and competing interests; and statements of data and code availability are available at 10.1038/s41586-022-05172-4.

### Supplementary information

Supplementary Information

## Figures and Tables

**Extended Data Table 1:**
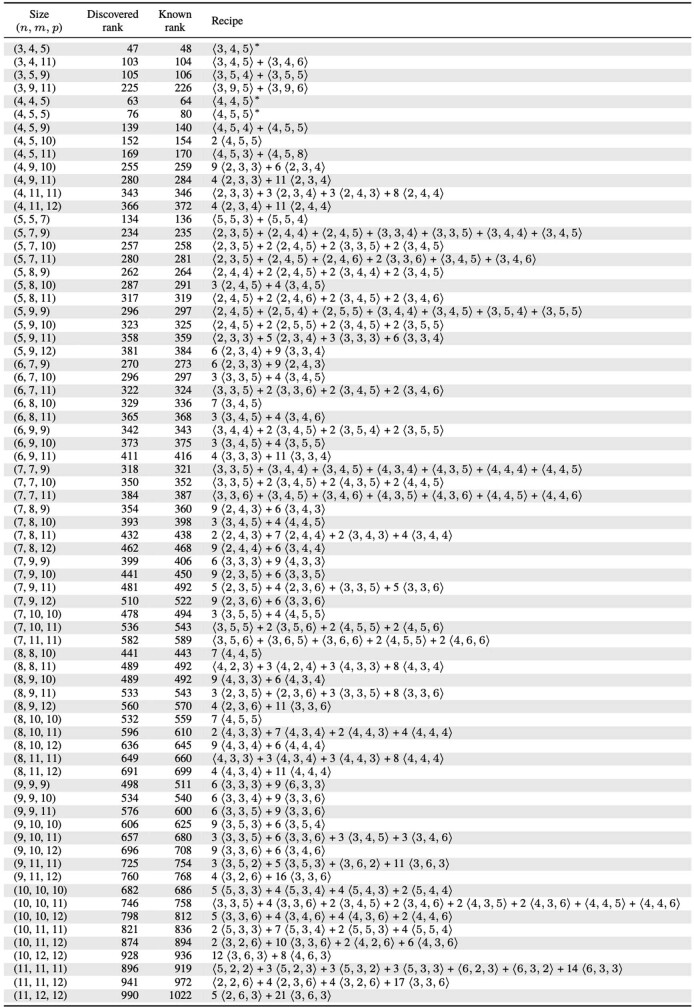
Rank results obtained by combining decompositions (in standard arithmetic)

The table shows the cases where we were able to obtain an improvement over state-of-the-art, for tensors ${{\mathscr{T}}}_{n,m,p}$ (with *n*, *m*, *p*≤12). The recipe column indicates the low-level matrix multiplication algorithms used to build the corresponding factorization. 〈*n*, *m*, *p*〉 denotes the best known bound on the rank of ${{\mathscr{T}}}_{n,m,p}$; see Appendix H in Supplementary Information for more details. For tensors that were directly decomposed by AlphaTensor, the recipe shows a star mark, e.g. 〈3, 4, 5〉^*^. All the factorizations are made available.

**Extended Data Table 2:**
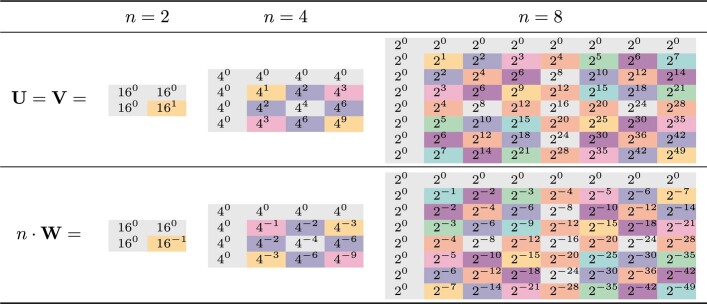
Result of applying AlphaTensor to the tensor representing the cyclic convolution operation

AlphaTensor finds the discrete Fourier matrix (DFT) and the inverse DFT matrix in finite fields. The figure shows the decompositions found by AlphaTensor of the *n* × *n* × *n* tensor representing the cyclic convolution of two vectors, for three different values of *n* in the finite field of order 17. The action space, characterized by the number of possible factor triplets {**u**^(*r*)^, **v**^(*r*)^, **w**^(*r*)^}, is thus 17^3*n*^, which is of the order of 10^29^ for *n* = 8. Despite the huge action space, AlphaTensor finds the optimal rank-*n* decompositions for the three values of *n*. The factors in the figure are stacked vertically, i.e., **U** = [**u**^(1)^, …, **u**^(*n*)^]. For ease of visualization, the factor entries have been expressed in terms of powers of an *n*-th primitive root of unity in the finite field. Within each column, each colour uniquely represents one element of the field (e.g., for the column *n* = 4, we have depicted in grey 4^0^ = 4^4^ = 4^−4^ = 1). By inspecting the patterns in the decompositions, one could extrapolate the results for other values of *n* and other fields. Indeed, the factors **u**^(*r*)^ and **v**^(*r*)^ correspond to the DFT coefficients, since ${u}_{k}^{(r)}={v}_{k}^{(r)}={z}^{kr}$, whereas the factors **w**^(*r*)^ correspond to the inverse DFT, since ${w}_{k}^{(r)}={z}^{-kr}/n$ for 0≤*k*, *r* < *n*, where *z* is an *n*-th primitive root of unity (i.e., *z*^*n*^ = 1 and *z*^*j*^ ≠ 1 for any 1≤*j* < *n*).

## Data Availability

The data used to train the system were generated synthetically according to the procedures explained in the paper. The algorithms discovered by AlphaTensor are available for download at https://github.com/deepmind/alphatensor.

## Code availability

An interactive notebook with code to check the non-equivalence of algorithms is provided. Moreover, the fast algorithms from the ‘Algorithm discovery results’ section on a GPU and a TPU are provided. These are available for download at https://github.com/deepmind/alphatensor. A full description of the AlphaZero algorithm that this work is based on is available in ref. ^1^, and the specific neural network architecture we use is described using pseudocode in the Supplementary Information.
