# Supplementary material for: Discovering faster matrix multiplication algorithms with reinforcement learning
Source: Nature. 2022 Oct 5;610(7930):47–53. doi: 10.1038/s41586-022-05172-4 (PMC9534758; doi:10.1038/s41586-022-05172-4)
Supplement: Supplementary file 1 — Supplementary Information [file 41586_2022_5172_MOESM1_ESM.pdf]

nature portfolio

https://doi.org/10.1038/s41586-022-05172-4

Supplementary information

Discovering faster matrix multiplication algorithms with reinforcement learning

In the format provided by the authors and unedited

Discovering faster matrix multiplication algorithms with reinforcement learning (Supplementary Information)

Alhussein Fawzi* Matej Balog* Aja Huang* Thomas Hubert*
Bernardino Romera-Paredes* Mohammadamin Barekatain† Alexander Novikov†
Francisco J. R. Ruiz† Julian Schrittwieser† Grzegorz Swirszcz† David Silver
Demis Hassabis Pushmeet Kohli

DeepMind, London, UK

Contents

A Additional details on methods **2**
- A.1 Details on network components . . . 2
  - A.1.1 Torso . . . 2
  - A.1.2 Policy head . . . 3
  - A.1.3 Value head . . . 4
  - A.1.4 Training and inference with *AlphaTensor* . . . 5
- A.2 Algorithm for generating synthetic demonstrations . . . 5
- A.3 Algorithm for generating changes of basis . . . 6
- A.4 Converting modular arithmetic algorithms into general ones . . . 6

B Symmetries in matrix multiplication algorithms **6**
- B.1 Equivalence of factorizations . . . 7
- B.2 Algorithm for certifying nonequivalence . . . 8
- B.3 Diversity of factorizations . . . 8

C Structured matrix multiplication **9**
- C.1 Circular matrix-vector product in finite fields . . . 9
- C.2 Skew-symmetric matrix-vector product . . . 11

D Rapid tailored algorithm discovery **13**
- D.1 Benchmarking details . . . 13
- D.2 Experimental setup . . . 15
- D.3 Additional results . . . 16

E Finding border rank with *AlphaTensor* **16**

F Ablations **18**

G Hyperparameters **19**

*Equal contributors. Authors listed alphabetically by last name, after corresponding author.
†Equal contributors. Authors listed alphabetically by last name.

H Combining smaller factorizations into bigger ones **19**
H.1 Illustrative example 20
H.2 General approach 21

I Proofs **22**
I.1 Proof of Theorem B.2 22
I.2 Proof of Theorem B.3 22
I.3 Proof for skew-symmetric matrix-vector product 24
I.3.1 Problem formulation and the results 25

A Additional details on methods

A.1 Details on network components

Algorithms A.1-A.9 show the details of each component. In Algorithm A.10, we show how the different components are connected together for training the neural network in *AlphaTensor*. In Algorithm A.11, we show how *AlphaTensor*'s neural network is used at inference time.

A.1.1 Torso

Algorithm A.1 Basic attention function.

def Attention $\left(\boldsymbol{x} \in \mathbb{R}^{N_x \times c_1}, \boldsymbol{y} \in \mathbb{R}^{N_y \times c_2}, \text{causal\_mask} = \text{False}, N_{\text{heads}} = 16, d = 32, w = 4\right)$

$\boldsymbol{x}_{\text{norm}} = \text{LayerNorm}(\boldsymbol{x})$

$\boldsymbol{y}_{\text{norm}} = \text{LayerNorm}(\boldsymbol{y})$

$\boldsymbol{q}_i^h = \text{Linear}(\boldsymbol{x}_{\text{norm}}, d)$ $\qquad \boldsymbol{q}_i^h \in \mathbb{R}^d, h \in \{1, \ldots, N_{\text{heads}}\}, i \in \{1, \ldots, N_x\}$

$\boldsymbol{k}_j^h = \text{Linear}(\boldsymbol{y}_{\text{norm}}, d)$ $\qquad \boldsymbol{k}_j^h \in \mathbb{R}^d, h \in \{1, \ldots, N_{\text{heads}}\}, j \in \{1, \ldots, N_y\}$

$\boldsymbol{v}_j^h = \text{Linear}(\boldsymbol{y}_{\text{norm}}, d)$ $\qquad \boldsymbol{v}_j^h \in \mathbb{R}^d, h \in \{1, \ldots, N_{\text{heads}}\}, j \in \{1, \ldots, N_y\}$

$a_{ij}^h = \text{softmax}_k \left(\frac{1}{\sqrt{d}} \boldsymbol{q}_i^{h\top} \boldsymbol{k}_j^h\right)$

if causal_mask **then**

  $\boldsymbol{o}_i^h = \sum_{j>i} a_{ij}^h \boldsymbol{v}_j^h$ $\qquad \boldsymbol{o}_i^h \in \mathbb{R}^d, h \in \{1, \ldots, N_{\text{heads}}\}, i \in \{1, \ldots, N_x\}$

else

  $\boldsymbol{o}_i^h = \sum_j a_{ij}^h \boldsymbol{v}_j^h$ $\qquad \boldsymbol{o}_i^h \in \mathbb{R}^d, h \in \{1, \ldots, N_{\text{heads}}\}, i \in \{1, \ldots, N_x\}$

$\boldsymbol{x} = \boldsymbol{x} + \text{Linear}\left(\text{concat}_h(\boldsymbol{o}_i^h), c_1\right)$

# Dense block:

$\boldsymbol{x} = \boldsymbol{x} + \text{Linear}\left(\text{GeLU}\left(\text{Linear}\left(\text{LayerNorm}(\boldsymbol{x}), c_1 w\right)\right), c_1\right)$

return $\boldsymbol{x}$

Algorithm A.2 Attentive modes.

def AttentiveModes $\left(\boldsymbol{x}_1 \in \mathbb{R}^{S \times S \times c}, \boldsymbol{x}_2 \in \mathbb{R}^{S \times S \times c}, \boldsymbol{x}_3 \in \mathbb{R}^{S \times S \times c}\right)$

$\boldsymbol{g} = [\boldsymbol{x}_1, \boldsymbol{x}_2, \boldsymbol{x}_3]$

for $[m_1, m_2] = \{[0,1], [2,0], [1,2]\}$ **do**

  $\boldsymbol{a} = \text{Concatenate}\left(\left[\boldsymbol{g}[m_1], \boldsymbol{g}[m_2]^\top\right], \text{axis} = 1\right)$ $\qquad \boldsymbol{a} \in \mathbb{R}^{S \times 2S \times c}$

  # Parallel loop

  for $i = \{0, \ldots, S-1\}$ **do**

    $\boldsymbol{c} = \text{Attention}(\boldsymbol{a}[i,:,:], \boldsymbol{a}[i,:,:])$ $\qquad \boldsymbol{c} \in \mathbb{R}^{2S \times c}$

    $\boldsymbol{g}[m_1][i,:,:] = \boldsymbol{c}[:S,:]$

    $\boldsymbol{g}[m_2][i,:,:] = \boldsymbol{c}[S:,:]^\top$

return $\boldsymbol{g}$

Algorithm A.3 Torso.

def Torso $\left(\boldsymbol{x} \in \mathbb{R}^{T\times S\times S\times S}, \boldsymbol{s} \in \mathbb{R}^{s}, c \in \mathbb{N}\right)$

$\boldsymbol{x}_1 = \text{Reshape}\left(\text{Transpose}\left(\boldsymbol{x}, (1,2,3,0)\right), (S \times S \times ST)\right)$ $\qquad \boldsymbol{x}_1 \in \mathbb{R}^{S\times S\times ST}$

$\boldsymbol{x}_2 = \text{Reshape}\left(\text{Transpose}\left(\boldsymbol{x}, (3,1,2,0)\right), (S \times S \times ST)\right)$ $\qquad \boldsymbol{x}_2 \in \mathbb{R}^{S\times S\times ST}$

$\boldsymbol{x}_3 = \text{Reshape}\left(\text{Transpose}\left(\boldsymbol{x}, (2,3,1,0)\right), (S \times S \times ST)\right)$ $\qquad \boldsymbol{x}_3 \in \mathbb{R}^{S\times S\times ST}$

$\boldsymbol{g} = [\boldsymbol{x}_1, \boldsymbol{x}_2, \boldsymbol{x}_3]$

for $i = \{0, \ldots, 2\}$ **do**

  $\boldsymbol{p} = \text{Reshape}\left(\text{Linear}\left(\boldsymbol{s}, S^2\right), (S, S, 1)\right)$ $\qquad \boldsymbol{p} \in \mathbb{R}^{S\times S\times 1}$

  $\boldsymbol{g}[i] = \text{Concatenate}\left([\boldsymbol{g}[i], \boldsymbol{p}], -1\right)$ $\qquad \boldsymbol{g}[i] \in \mathbb{R}^{S\times S\times ST+1}$

  $\boldsymbol{g}[i] = \text{Linear}\left(\boldsymbol{g}[i], c\right)$ $\qquad \boldsymbol{g}[i] \in \mathbb{R}^{S\times S\times c}$

$[\boldsymbol{x}_1, \boldsymbol{x}_2, \boldsymbol{x}_3] = \boldsymbol{g}$

for $i = \{1, \ldots, 8\}$ **do**

  $[\boldsymbol{x}_1, \boldsymbol{x}_2, \boldsymbol{x}_3] = \text{AttentiveModes}\left(\boldsymbol{x}_1, \boldsymbol{x}_2, \boldsymbol{x}_3\right)$ $\qquad \boldsymbol{x}_1, \boldsymbol{x}_2, \boldsymbol{x}_3 \in \mathbb{R}^{S\times S\times c}$

$\boldsymbol{e} = \text{Reshape}\left(\text{Stack}\left([\boldsymbol{x}_1, \boldsymbol{x}_2, \boldsymbol{x}_3], \text{axis} = 1\right), \left(3S^2, c\right)\right)$ $\qquad \boldsymbol{e} \in \mathbb{R}^{3S^2\times c}$

return $\boldsymbol{e}$

A.1.2 Policy head

Algorithm A.4 Function used in the policy head to map from the learned embedding obtained from the torso, $\boldsymbol{e}$, and the piece of action produced so far (or ground truth if training), $\boldsymbol{a}$, to the logits of the remainder of the action.

def predict_action_logits $\left(\boldsymbol{a} \in \mathbb{R}^{N_{\text{steps}}\times N_{\text{logits}}}, \boldsymbol{e} \in \mathbb{R}^{m\times c}, N_{\text{features}} = 64, N_{\text{heads}} = 32, N_{\text{layers}} = 2\right)$

$\boldsymbol{x} = \text{Linear}\left(\boldsymbol{a}, N_{\text{features}} \times N_{\text{heads}}\right)$ $\qquad \boldsymbol{x} \in \mathbb{R}^{N_{\text{steps}}\times N_{\text{features}}N_{\text{heads}}}$

$\boldsymbol{x} = \boldsymbol{x} + \text{LearnablePositionEncoding}\left(\boldsymbol{x}\right)$

for $i \in \{1 \ldots N_{\text{layers}}\}$ **do**

  $\boldsymbol{x} = \text{LayerNorm}(\boldsymbol{x})$

  # Causal self attention:

  $\boldsymbol{c} = \text{Attention}(\boldsymbol{x}, \boldsymbol{x}, N_{\text{heads}}, \text{causal\_mask} = \text{True})$ $\qquad \boldsymbol{c} \in \mathbb{R}^{N_{\text{steps}}\times N_{\text{features}}N_{\text{heads}}}$

  if is training **then**

    $\boldsymbol{c} = \text{Dropout}(\boldsymbol{c})$

  $\boldsymbol{x} = \boldsymbol{x} + \boldsymbol{c}$

  $\boldsymbol{x} = \text{LayerNorm}(\boldsymbol{x})$

  # Cross attention:

  $\boldsymbol{c} = \text{Attention}(\boldsymbol{x}, \boldsymbol{e}, N_{\text{heads}}, \text{causal\_mask} = \text{False})$

  if is training **then**

    $\boldsymbol{c} = \text{Dropout}(\boldsymbol{c})$

  $\boldsymbol{x} = \boldsymbol{x} + \boldsymbol{c}$

$\boldsymbol{o} = \text{Linear}(\text{ReLU}(\boldsymbol{x}), N_{\text{logits}})$ $\qquad \boldsymbol{o} \in \mathbb{R}^{N_{\text{steps}}\times N_{\text{logits}}}$

return $\boldsymbol{o}, \boldsymbol{x}$

Algorithm A.5 Policy head behaviour at training time. It returns the logits (and the embeddings of the first step).

def PolicyHead_training $\left(\boldsymbol{e} \in \mathbb{R}^{m\times c}, \boldsymbol{g} \in \{1, \ldots, N_{\text{logits}}\}^{N_{\text{steps}}}\right)$

# Training by teacher-forcing:

$\boldsymbol{o}, \boldsymbol{z} = \text{predict\_action\_logits}(\text{onehot}(\text{shifted}(\boldsymbol{g}), N_{\text{logits}}), \boldsymbol{e})$

# Returns only $\boldsymbol{z}_1$ *because it is the only one not conditioned on ground truth.*

return $\boldsymbol{o}, \boldsymbol{z}_1$

Algorithm A.6 Policy head behaviour at inference time. It returns the sampled action, and its estimated probability (and the embeddings of the first step).

def PolicyHead_inference $(\boldsymbol{e} \in \mathbb{R}^{m \times c}, N_{\text{samples}} = 32, N_{\text{steps}}, N_{\text{logits}})$

$a_i^s = 0$ — $s \in \{1, \ldots, N_{\text{samples}}\}, i \in \{1, \ldots, N_{\text{steps}}\}$

$p^s = 1$ — $s \in \{1, \ldots, N_{\text{samples}}\}$

for $s \in \{1, \ldots, N_{\text{samples}}\}$ **do**

  for $i \in \{1, \ldots, N_{\text{steps}}\}$ **do**

    $\boldsymbol{o}^s, \boldsymbol{z}^s = \text{predict\_action\_logits}(\text{onehot}(\boldsymbol{a}^s, N_{\text{logits}}), \boldsymbol{e})$ — $\boldsymbol{o}^s \in \mathbb{R}^{N_{\text{steps}} \times N_{\text{logits}}}, \boldsymbol{z}^s \in \mathbb{R}^{N_{\text{steps}} \times 2048}$

    $a_i^s, p_i = \text{sample\_from\_logits}(\boldsymbol{o}_i^s)$

    $p^s = p^s \odot p_i$

# Returns only $\boldsymbol{z}_1$ because it is the only one not conditioned on any sampled action.

return $\boldsymbol{a}, \boldsymbol{p}, \boldsymbol{z}_1$ — $\boldsymbol{a} \in \{1, \ldots, N_{\text{logits}}\}^{N_{\text{samples}} \times N_{\text{steps}}}, \boldsymbol{p} \in [0, 1]^{N_{\text{samples}}}, \boldsymbol{z}_1 \in \mathbb{R}^{2048}$

A.1.3 Value head

Algorithm A.7 Value head network.

def ValueHead $(\mathbf{x} \in \mathbb{R}^c, n = 8)$

for $i \in \{1, 2, 3\}$ **do**

  $\boldsymbol{x} = \text{ReLU}(\text{Linear}(\boldsymbol{x}, 512))$

$\boldsymbol{q} = \text{Linear}(\boldsymbol{x}, n)$ — $\boldsymbol{q} \in \mathbb{R}^n$

return $\boldsymbol{q}$

Algorithm A.8 Quantile loss used in the value head. $\boldsymbol{q}$ are the predicted values of the quantiles at equally spaced intervals, $\boldsymbol{g}$ is the ground truth.

def Quantile_loss $(\boldsymbol{q} \in \mathbb{R}^n, \boldsymbol{g} \in \mathbb{R}^n, \delta = 1)$

# Getting the n quantiles, $\boldsymbol{\tau}$.

$\boldsymbol{\tau} = ([0 \ldots n] + 0.5) / n$ — $\boldsymbol{\tau} \in [0, 1]^n$

$\boldsymbol{d} = \boldsymbol{g} - \boldsymbol{q}$ — $\boldsymbol{d} \in \mathbb{R}^n$

$\boldsymbol{h} = \text{Huber\_loss}(\boldsymbol{d}, \delta)$ — $\boldsymbol{h} \in \mathbb{R}^n$

$\boldsymbol{k} = \text{abs}(\boldsymbol{\tau} - \text{float}(\boldsymbol{d} < 0))$ — $\boldsymbol{k} \in \mathbb{R}^n$

return $\text{mean}(\boldsymbol{k} \odot \boldsymbol{h})$

Algorithm A.9 Obtaining the predicted value at inference.

def ValueRiskManagement $(\boldsymbol{q} \in \mathbb{R}^n, u_q = 0.75)$

$j = \lceil u_q n \rceil$ — $j \in [1, \ldots, n]$

return $\text{mean}(\boldsymbol{q}[j], \boldsymbol{q}[j+1], \ldots \boldsymbol{q}[n])$

A.1.4 Training and inference with *AlphaTensor*

Algorithm A.10 Training *AlphaTensor*'s neural network.

def *AlphaTensor*_Net_training $\left(\boldsymbol{x} \in \mathbb{R}^{T\times S\times S\times S}, \boldsymbol{s} \in \mathbb{R}^{s}, \boldsymbol{g}_{\text{action}} \in \{1,\ldots,N_{\text{logits}}\}^{N_{\text{steps}}}, \boldsymbol{g}_{\text{value}} \in \mathbb{R}^{n}, c \in \mathbb{N}\right)$

$\boldsymbol{e} = \text{Torso}\left(\boldsymbol{x}, \boldsymbol{s}, c\right)$ — $\boldsymbol{e} \in \mathbb{R}^{3S^2\times c}$

$\boldsymbol{o}, \boldsymbol{z}_1 = \text{PolicyHead\_training}\left(\boldsymbol{e}, \boldsymbol{g}_{\text{action}}, N_{\text{logits}}\right)$ — $\boldsymbol{o} \in \mathbb{R}^{N_{\text{steps}}\times N_{\text{logits}}}, \boldsymbol{z}_1 \in \mathbb{R}^{2048}$

$l_{\text{policy}} = \text{Sum}\left(\text{CrossEntropy}(\boldsymbol{o}, \boldsymbol{g}_{\text{action}})\right)$

$\boldsymbol{q} = \text{ValueHead}\left(\mathbf{z}_1\right)$ — $\boldsymbol{q}, \boldsymbol{\tau} \in \mathbb{R}^{N_{\text{quantile\_samples}}}$

$l_{\text{value}} = \text{Quantile\_loss}\left(\boldsymbol{q}, \boldsymbol{g}_{\text{value}}\right)$

return $l_{\text{policy}}, l_{\text{value}}$

Algorithm A.11 Using *AlphaTensor*'s neural network for inference.

def *AlphaTensor*_Net_inference $\left(\boldsymbol{x} \in \mathbb{R}^{T\times S\times S\times S}, \boldsymbol{s} \in \mathbb{R}^{s}, c \in \mathbb{N}, N_{\text{samples}} \in \mathbb{N}, N_{\text{steps}} \in \mathbb{N}, N_{\text{logits}} \in \mathbb{N}\right)$

$\boldsymbol{e} = \text{Torso}\left(\boldsymbol{x}, \boldsymbol{s}, c\right)$ — $\boldsymbol{e} \in \mathbb{R}^{3S^2\times c}$

$\boldsymbol{a}, \boldsymbol{p}, \boldsymbol{z}_1 = \text{PolicyHead\_inference}\left(\boldsymbol{e}, N_{\text{samples}}, N_{\text{steps}}, N_{\text{logits}}\right)$ — $\boldsymbol{a} \in \{1,\ldots,N_{\text{logits}}\}^{N_{\text{samples}}\times N_{\text{steps}}}, \boldsymbol{p} \in [0,1]^{N_{\text{samples}}},$ $\boldsymbol{z}_1 \in \mathbb{R}^{2048}$

$\boldsymbol{q} = \text{ValueHead}\left(\mathbf{z}_1\right)$ — $\boldsymbol{q} \in \mathbb{R}^{n}$

$q = \text{ValueRiskManagement}\left(\boldsymbol{q}\right)$ — $q \in \mathbb{R}$

return $\boldsymbol{a}, \boldsymbol{p}, q$

A.2 Algorithm for generating synthetic demonstrations

Algorithm A.12: Generation of synthetic demonstrations

Input: Tensor size S, maximum rank R_{limit}, factor entry probability distribution p_{entry}, desired number of demonstrations N, random seed.

Output: N pairs $(\mathfrak{T}, \{\mathbf{u}^{(r)}, \mathbf{v}^{(r)}, \mathbf{w}^{(r)}\}_{r=1}^{R})$ such that in each $\mathfrak{T} = \sum_{r=1}^{R} \mathbf{u}^{(r)} \otimes \mathbf{v}^{(r)} \otimes \mathbf{w}^{(r)}$.

1: **for** $n = 1$ **to** N **do**
2: $\quad R \sim \text{Uniform}(\{1, 2, \ldots, R_{\text{limit}}\})$ ▷ Sample the rank of the synthetic demonstration.
3: $\quad$ **for** $r = 1$ **to** R **do**
4: $\quad\quad$ **repeat**
5: $\quad\quad\quad$ **for** $s = 1$ **to** S **do**
6: $\quad\quad\quad\quad \mathbf{u}_s^{(r)} \sim p_{\text{entry}}$
7: $\quad\quad\quad\quad \mathbf{v}_s^{(r)} \sim p_{\text{entry}}$
8: $\quad\quad\quad\quad \mathbf{w}_s^{(r)} \sim p_{\text{entry}}$
9: $\quad\quad$ **until** $\mathbf{u}^{(r)} \otimes \mathbf{v}^{(r)} \otimes \mathbf{w}^{(r)} \neq \mathbf{0}$
10: $\quad \mathfrak{T} \leftarrow \sum_{r=1}^{R} \mathbf{u}^{(r)} \otimes \mathbf{v}^{(r)} \otimes \mathbf{w}^{(r)}$ ▷ Reconstruct tensor corresponding to the random factors.
11: $\quad$ **yield** $(\mathfrak{T}, \{\mathbf{u}^{(r)}, \mathbf{v}^{(r)}, \mathbf{w}^{(r)}\}_{r=1}^{R})$

Algorithm A.12 shows the procedure we used for generating the synthetic demonstrations. The N data points are sampled i.i.d., corresponding to the outer loop on Line 1. When generating demonstrations in a ring $\mathcal{E}$, factor entries are sampled from the set $\mathcal{E}$ and the arithmetic (multiplications and additions in Line 10) is performed in this ring, e.g., in $\mathbb{Z}_2$ this corresponds to applying an additional modulo operation.

Hyper-parameters. In all experiments we used $N = 5{,}000{,}000$ synthetic demonstrations. We use the factor entry probability distribution $p_{\text{entry}}(-2) = 0.001$, $p_{\text{entry}}(-1) = 0.099$, $p_{\text{entry}}(0) = 0.8$, $p_{\text{entry}}(1) = 0.099$, $p_{\text{entry}}(2) = 0.001$ for standard arithmetic, and $p_{\text{entry}}(0) = p_{\text{entry}}(1) = 0.5$ for modular arithmetic in $\mathbb{Z}_2$.

A.3 Algorithm for generating changes of basis

Algorithm A.13: Generation of basis change matrices

Input: Tensor size S, matrix entry distribution $p_{\text{cob-entry}}$, desired number of basis changes N_{cob}, random seed.
Output: N_{cob} i.i.d. invertible unimodular $S \times S$ matrices.

for $n = 1$ **to** N_{cob} **do**
  $\mathbf{P} \leftarrow$ zero $S \times S$ matrix ▷ Will be upper-triangular.
  $\mathbf{L} \leftarrow$ zero $S \times S$ matrix ▷ Will be lower-triangular.
  for $i = 1$ **to** S **do**
    $\mathbf{P}_{ii} \sim \text{Uniform}(\{-1, +1\})$
    $\mathbf{L}_{ii} \sim \text{Uniform}(\{-1, +1\})$
  for $i = 1$ **to** S **do**
    for $j = 1$ **to** $i - 1$ **do**
      $\mathbf{L}_{ij} \sim p_{\text{cob-entry}}$
    for $j = i + 1$ **to** S **do**
      $\mathbf{P}_{ij} \sim p_{\text{cob-entry}}$
  yield $\mathbf{PL}$

Algorithm A.13 shows the procedure for generating basis change matrices. We chose to generate unimodular change of bases for numerical stability of the obtained factorizations after converting them back into the canonical basis. The matrices generated by Algorithm A.13 are unimodular because the determinant of a triangular matrix equals the product of its diagonal elements (-1 or $+1$ in this case), and so $\det(\mathbf{PL}) = \det \mathbf{P} \det \mathbf{L} \in \{-1, +1\}$ by construction. In our experiments we used $p_{\text{cob-entry}}(0) = 0.985$ and $p_{\text{cob-entry}}(-1) = p_{\text{cob-entry}}(1) = 0.0075$.

In acting, an actor is given a basis in which it should try to decompose the tensor (see Figure 2 in the main paper). With probability $p_{\text{canonical}}$ the basis given is the original (canonical) basis (i.e., no basis change is applied), and with probability $1 - p_{\text{canonical}}$ one of the N_{cob} generated basis changes is chosen uniformly at random. In our experiments $N_{\text{cob}} = 100{,}000$ and $p_{\text{canonical}} = \frac{250}{100{,}000} \approx 0.0017$.

A.4 Converting modular arithmetic algorithms into general ones

As for the targets $\mathcal{T}_4$ and $\mathcal{T}_5$ *AlphaTensor* discovered algorithms of lower rank in modular arithmetic $\mathbb{Z}_2$ than in standard arithmetic, we attempted to convert these modular arithmetic algorithms into general ones using a SAT solver. Specifically, given a factorization with entries $F = \{0, 1\}$ valid in the ring $\mathcal{E} = \mathbb{Z}_2$, we set up the problem of finding a factorization with entries $F = \{-1, 0, 1\}$ valid in the standard arithmetic $\mathcal{E} = \mathbb{Z}$ such that it has the same sparsity pattern but each 1 entry in the $\mathbb{Z}_2$ factorization is turned into either a -1 or $+1$ entry in the $\mathbb{Z}$ factorization. This follows the approach of [1]. Unfortunately, the SAT solver was able to prove that it is not possible to convert our rank-47 algorithms for $\mathcal{T}_4$ nor the rank-96 algorithms for $\mathcal{T}_5$ from $\mathbb{Z}_2$ into standard arithmetic in this way. This of course does not preclude that other algorithms of this rank may exist for these targets.

B Symmetries in matrix multiplication algorithms

In Section B.1, we define the equivalence relation between matrix multiplication algorithms. Section B.2 details the algorithm used for determining whether two algorithms are equivalent. Section B.3 provides details on the different non-equivalent factorizations found using *AlphaTensor*.

Notation. Recall that $\otimes$ defines the tensor product operation. That is, if $\mathbf{T}_1$ and $\mathbf{T}_2$ are two tensors with n and m dimensions, respectively, then $\mathbf{T}_1 \otimes \mathbf{T}_2$ is the tensor with $n + m$ dimensions where

$$(\mathbf{T}_1 \otimes \mathbf{T}_2)[i_1, i_2, \ldots, i_n, j_1, \ldots, j_m] = \mathbf{T}_1[i_1, \ldots, i_n]\mathbf{T}_2[j_1, \ldots, j_m].$$

A slight variation of the tensor product is the Kronecker product $\otimes_K$, which we define between two tensors with the same number of dimensions: if $\mathbf{T}_1$ and $\mathbf{T}_2$ have sizes $[s_1, \ldots, s_n]$ and $[t_1, \ldots, t_n]$, respectively,

$$(\mathbf{T}_1 \otimes_K \mathbf{T}_2)[t_1 i_1 + j_1, \ldots, t_n i_n + j_n] = \mathbf{T}_1[i_1, \ldots, i_n]\mathbf{T}_2[j_1, \ldots, j_n].$$

For convenience, in Sections B.1-B.3 we will see factor vectors of $\mathfrak{T}_n$ as $n \times n$ matrices, rather than vectors of length n^2, and hence denote them with capital letters $\mathbf{U}, \mathbf{V}, \mathbf{W}$. If $\{(\mathbf{U}_p, \mathbf{V}_p, \mathbf{W}_p)\}_{p=1}^R$ and $\{(\mathbf{U}'_q, \mathbf{V}'_q, \mathbf{W}'_q)\}_{q=1}^R$ are factorizations of $\mathfrak{T}$ then the following provides a rank R^2 factorization of $\mathfrak{T}^{\otimes 2} = \mathfrak{T} \otimes_K \mathfrak{T}$:

$$\{(\mathbf{U}_p \otimes_K \mathbf{U}'_q, \mathbf{V}_p \otimes_K \mathbf{V}'_q, \mathbf{W}_p \otimes_K \mathbf{W}'_q)\}_{p,q=1}^R. \tag{1}$$

Finally, we denote as S_n the group of permutations of n elements.

B.1 Equivalence of factorizations

Since the matrix multiplication tensors have a product structure (that is, $\mathfrak{T}_4 = \mathfrak{T}_2^{\otimes 2} = \mathfrak{T}_2 \otimes_K \mathfrak{T}_2$), one can obtain using Eq. 1 a rank 49 factorization of $\mathfrak{T}_4$ from a rank 7 factorization (Strassen) of $\mathfrak{T}_2$. Such product-structure factorizations are the only known schemes to decompose $\mathfrak{T}_4$ into 49 factors, and are referred to in the main paper as Strassen^2.[1]

Our goal here is to show that the factorizations obtained using *AlphaTensor* are not *equivalent* to the known factorization of product structure, and that these factorizations are pairwise nonequivalent. To do so, we first define the notion of *equivalence*.

When seen as a trilinear map, the (transposed) matrix multiplication operation is $(\mathbf{X}_1, \mathbf{X}_2, \mathbf{X}_3) \to \text{Trace}(\mathbf{X}_1\mathbf{X}_2\mathbf{X}_3)$. Using the invariance of trace to cyclic permutations, $\text{Trace}(\mathbf{X}_1\mathbf{X}_2\mathbf{X}_3) = \text{Trace}(\mathbf{X}_2\mathbf{X}_3\mathbf{X}_1) = \text{Trace}(\mathbf{X}_3\mathbf{X}_1\mathbf{X}_2)$. Since transposition does not change the trace either, we also have

$$\text{Trace}(\mathbf{X}_1\mathbf{X}_2\mathbf{X}_3) = \text{Trace}(\mathbf{X}_3^T\mathbf{X}_2^T\mathbf{X}_1^T) = \text{Trace}(\mathbf{X}_2^T\mathbf{X}_1^T\mathbf{X}_3^T) = \text{Trace}(\mathbf{X}_1^T\mathbf{X}_3^T\mathbf{X}_2^T).$$

Similarly, for any invertible matrices $\mathbf{A}, \mathbf{B}, \mathbf{C}$,

$$\text{Trace}(\mathbf{X}_1\mathbf{X}_2\mathbf{X}_3) = \text{Trace}((\mathbf{A}\mathbf{X}_1\mathbf{B}^{-1})(\mathbf{B}\mathbf{X}_2\mathbf{C}^{-1})(\mathbf{C}\mathbf{X}_3\mathbf{A}^{-1})).$$

These invariances imply that, given a factorization $\{(\mathbf{U}_r, \mathbf{V}_r, \mathbf{W}_r)\}_{r=1}^R$ of $\mathfrak{T}_n$, we can generate many equivalent factorizations of $\mathfrak{T}_n$. Such symmetries have been heavily studied for $n = 2$ and $n = 3$ [3, 4, 5, 6, 7, 1]. We use the group of symmetries introduced in [3], which proved that Strassen's algorithm for multiplying 2×2 matrices is unique up to the actions of this symmetry group.

A permutation $\pi \in S_3$ acts on a rank one tensor $\mathbf{U} \otimes \mathbf{V} \otimes \mathbf{W}$ by permuting the factors $\mathbf{U}, \mathbf{V}, \mathbf{W}$ with an additional transposition when the signature of π is -1. For example, for the cyclic permutation $(1, 2, 3)$, we have $(1, 2, 3) \cdot (\mathbf{U} \otimes \mathbf{V} \otimes \mathbf{W}) = \mathbf{V} \otimes \mathbf{W} \otimes \mathbf{U}$, and for the transposition $(1, 2)$, we have $(1, 2) \cdot (\mathbf{U} \otimes \mathbf{V} \otimes \mathbf{W}) = (\mathbf{V}^T \otimes \mathbf{U}^T \otimes \mathbf{W}^T)$. Given three invertible matrices $(\mathbf{A}, \mathbf{B}, \mathbf{C})$, their action on a rank one tensor is given by $(\mathbf{A}, \mathbf{B}, \mathbf{C}) \cdot (\mathbf{U} \otimes \mathbf{V} \otimes \mathbf{W}) = (\mathbf{A}\mathbf{U}\mathbf{B}^{-1} \otimes \mathbf{B}\mathbf{V}\mathbf{C}^{-1} \otimes \mathbf{C}\mathbf{W}\mathbf{A}^{-1})$. The two actions are combined as follows: given $(\mathbf{A}, \mathbf{B}, \mathbf{C}, \pi)$, the permutation π is applied followed by the $(\mathbf{A}, \mathbf{B}, \mathbf{C})$ action. For example, if $\pi = (1, 2, 3)$, then $(\mathbf{A}, \mathbf{B}, \mathbf{C}, \pi) \cdot (\mathbf{U} \otimes \mathbf{V} \otimes \mathbf{W}) = (\mathbf{A}\mathbf{V}\mathbf{B}^{-1} \otimes \mathbf{B}\mathbf{W}\mathbf{C}^{-1} \otimes \mathbf{C}\mathbf{U}\mathbf{A}^{-1})$.

Two factorizations $\{(\mathbf{U}_r, \mathbf{V}_r, \mathbf{W}_r)\}_{r=1}^R$ and $\{(\mathbf{U}'_r, \mathbf{V}'_r, \mathbf{W}'_r)\}_{r=1}^R$ are said to be *equivalent* if there exists $(\mathbf{A}, \mathbf{B}, \mathbf{C}, \pi)$ that maps the two sets of factors through the action described above: that is, there exists a permutation $\sigma \in S_R$ such that for all $r \in [R]$, we have

$$\mathbf{U}_r \otimes \mathbf{V}_r \otimes \mathbf{W}_r = (\mathbf{A}, \mathbf{B}, \mathbf{C}, \pi) \cdot (\mathbf{U}'_{\sigma(r)} \otimes \mathbf{V}'_{\sigma(r)} \otimes \mathbf{W}'_{\sigma(r)}).$$

We denote the equivalence as follows: $\{(\mathbf{U}_r \otimes \mathbf{V}_r \otimes \mathbf{W}_r)\}_{r=1}^R \overset{\mathbf{A},\mathbf{B},\mathbf{C},\pi}{\sim} \{(\mathbf{U}'_r \otimes \mathbf{V}'_r \otimes \mathbf{W}'_r)\}_{r=1}^R$. For more details on symmetries of factorizations, see e.g., [5].

Using this notion of equivalence, [3] showed that all rank 7 factorizations of $\mathfrak{T}_2$ are equivalent. We show that this is *not* the case for $\mathfrak{T}_4$, and we exhibit thousands of nonequivalent rank 49 factorizations of $\mathfrak{T}_4$.

Theorem B.1. *There exist at least* 14,235 *non-equivalent rank-*49 *decompositions of the matrix multiplication tensor* $\mathfrak{T}_4$. *These decompositions are not of product structure, i.e., they are not equivalent to Strassen*^{2}.

The procedure for certifying non-equivalence of factorizations is provided in Section B.2.

[1] Note that Winograd's algorithm [2] involves 48 multiplications, but it only applies to commutative rings and does not give bounds on the tensor rank. Winograd's algorithm cannot be applied recursively on larger matrices.

B.2 Algorithm for certifying nonequivalence

We now explain the procedure we used to certify the nonequivalence of factorizations, and prove the result in Theorem B.1. To determine whether two factorizations are equivalent, we define invariants under the transformation introduced in Section B.1. The first invariant we use is the matrix rank invariant, given by

$$\mathcal{R}(\{\mathbf{U}_r, \mathbf{V}_r, \mathbf{W}_r\}_{r=1}^R) = \{\{\text{rank}(\mathbf{U}_r), \text{rank}(\mathbf{V}_r), \text{rank}(\mathbf{W}_r)\}\}_{r=1}^R\,, \tag{2}$$

where $\{\cdot\}$ denotes an unordered tuple. $\mathcal{R}$ clearly satisfies the invariance under transformations in Section B.1. Using this invariant, we obtained 38 distinct factorizations with different invariants $\mathcal{R}$ (amongst more than 50,000 factorizations found by *AlphaTensor*). This invariant has been used extensively in prior work to check for equivalent factorizations (see e.g., [5]).

To further distinguish between non-equivalent factorizations, we developed a new invariant. This invariant is only applicable to a specific subset of factorizations, but on this subset it is much more granular than $\mathcal{R}$. More specifically, the new invariant is applicable to those factorizations that have exactly one factor such that the product of matrices is identity; i.e. without loss of generality $\mathbf{U}_1\mathbf{V}_1\mathbf{W}_1 = I$. (Note that this property is itself invariant under the transformations in Section B.1.) This property was satisfied by many but not all factorizations found by *AlphaTensor*. For factorizations satisfying this property we then define the invariant as follows:

$$\mathcal{K}(\{\mathbf{U}_r, \mathbf{V}_r, \mathbf{W}_r\}_{r=1}^R) = \{\text{CharPoly}\left(\Phi(\mathbf{U}_r \otimes_K \mathbf{V}_r \otimes_K \mathbf{W}_r)\right)\}_{r=1}^R\,,$$

where $\Phi(\mathbf{U}_r \otimes \mathbf{V}_r \otimes \mathbf{W}_r) = (I \otimes_K \mathbf{W}_1^{-1}\mathbf{V}_1^{-1} \otimes_K \mathbf{W}_1^{-1})(\mathbf{U}_r \otimes_K \mathbf{V}_r \otimes_K \mathbf{W}_r)(\mathbf{V}_1\mathbf{W}_1 \otimes_K \mathbf{W}_1 \otimes I)$, and CharPoly denotes the characteristic polynomial $\text{CharPoly}(\mathbf{A})(x) = \det(xI - \mathbf{A})$. Intuitively, Φ is a canonicalization operator that applies the $(\mathbf{A}, \mathbf{B}, \mathbf{C}) = (I, \mathbf{W}_1^{-1}\mathbf{V}_1^{-1}, \mathbf{W}_1^{-1})$ action to each rank one tensor in the factorization, so that the first factor becomes $\tilde{\mathbf{U}}_1 \otimes \tilde{\mathbf{V}}_1 \otimes \tilde{\mathbf{W}}_1 := \Phi(\mathbf{U}_1 \otimes \mathbf{V}_1 \otimes \mathbf{W}_1) = I \otimes I \otimes I$. The following theorem states that $\mathcal{K}$ is an invariant:

Theorem B.2. *If $\{\mathbf{U}_r, \mathbf{V}_r, \mathbf{W}_r\}_{r=1}^R$ and $\{\mathbf{U}'_r, \mathbf{V}'_r, \mathbf{W}'_r\}_{r=1}^R$ are equivalent and $\mathbf{U}_1\mathbf{V}_1\mathbf{W}_1 = \mathbf{U}'_1\mathbf{V}'_1\mathbf{W}'_1 = I$, then*

$$\mathcal{K}\left(\{\mathbf{U}_r, \mathbf{V}_r, \mathbf{W}_r\}_{r=1}^R\right) = \mathcal{K}\left(\{\mathbf{U}'_r, \mathbf{V}'_r, \mathbf{W}'_r\}_{r=1}^R\right).$$

See Section I.1 for the proof. Amongst all the factorizations obtained from *AlphaTensor* where this invariant is applicable, there are 14,207 factorizations that have a different value of $\mathcal{K}$. Together with 28 nonequivalent factorizations where this invariant is not applicable, these form a set of 14,235 non-equivalent rank-49 factorizations of $\mathcal{T}_4$. See the Python notebook in the supplementary material for the actual computation.

We further check that the factorizations obtained using *AlphaTensor* are not of product structure. To do so, we rely on the following result, which shows that it is sufficient to check that $\{(\mathbf{U}_r, \mathbf{V}_r, \mathbf{W}_r)\}_{r=1}^{49}$ is nonequivalent to one factorization of product form to infer that it is nonequivalent to *all* factorizations of product form.

Theorem B.3. *Let $\{(\mathbf{U}_r, \mathbf{V}_r, \mathbf{W}_r)\}_{r=1}^{49}$ be a rank 49 factorization of $\mathcal{T}_4$. Let $\{(\mathbf{S}_p^1, \mathbf{S}_p^2, \mathbf{S}_p^3)\}_{p=1}^7$ be a rank 7 factorization of $\mathcal{T}_2$ (i.e., a Strassen scheme for multiplying 2×2 matrices). The following two statements are equivalent:*

1. *$\{(\mathbf{U}_r, \mathbf{V}_r, \mathbf{W}_r)\}_{r=1}^{49}$ is equivalent to a factorization of product form $\{(\mathbf{X}_p \otimes_K \mathbf{X}'_q, \mathbf{Y}_p \otimes_K \mathbf{Y}'_q, \mathbf{Z}_p \otimes_K \mathbf{Z}'_q)\}_{p,q=1}^7$.*
2. *There exist permutations $\pi, \pi' \in S_3$ such that $\{(\mathbf{U}_r, \mathbf{V}_r, \mathbf{W}_r)\}_{r=1}^{49}$ is equivalent to*

$$\left\{\left(T_\pi(\mathbf{S}_p^{\pi(1)}) \otimes_K T_{\pi'}(\mathbf{S}_q^{\pi'(1)}), T_\pi(\mathbf{S}_p^{\pi(2)}) \otimes_K T_{\pi'}(\mathbf{S}_q^{\pi'(2)}), T_\pi(\mathbf{S}_p^{\pi(3)}) \otimes_K T_{\pi'}(\mathbf{S}_q^{\pi'(3)})\right)\right\}_{p,q=1}^7.$$

where T_π is the transpose operator when the signature of π is -1, and identity otherwise.

See Section I.2 for the proof.

B.3 Diversity of factorizations

The multitude of nonequivalent factorizations discovered by *AlphaTensor* demonstrates that this space is richer than previously thought. Without further manipulations or optimizing for different objectives, the rank-49 factorizations of $\mathcal{T}_4$ discovered by *AlphaTensor* have the following properties:

- **Entries** in $\{-4, -3, -2, -1, 0, 1, 2\}$. The reason we were able to discover factorizations with entries outside of the set $F = \{-2, -1, 0, 1, 2\}$ that the network can predict is that after converting an F-valued factorization obtained in a different basis back into the canonical basis, its entries can end up outside of F. The entries are still integral because we use unimodular basis change matrices.
- **Matrix ranks** of the factor vectors (when seen as 4×4 matrices) in the factorizations form 38 different *types* [8, 5, 1, 9]. Represented as a three-variable polynomial, the type of Strassen^2 is $p(x, y, z) = x^4y^4z^4 + 12x^2y^2z^2 + 36xyz$ (there is 1 factor with all three vectors of matrix rank 4, 12 factors with all three vectors of matrix rank 2, and 36 factors with all three vectors of matrix rank 1), and *AlphaTensor* also discovers many factorizations of this type. However, *AlphaTensor* also discovers factorizations with 37 new types.
- **Sparsity.** The number of nonzeros in our discovered factorizations ranges from 455 to 636.
- **Cyclicity.** Around 0.2% of the discovered factorizations are *cyclic* (closed under permuting $(\mathbf{U}, \mathbf{V}, \mathbf{W})$ with a cyclic element of S_3); the vast majority are noncyclic.
- **Numerical stability.** [10] identified two principal quantities that govern the numerical error bounds of matrix multiplication algorithms: a prefactor Q, and a stability factor E. Lower values correspond to tighter bounds. For the Strassen^2 algorithm $Q = 24$ and $E = 144$, while for the discovered factorizations $Q \in [23, 39]$ and $E \in [136, 1046]$. In particular, *AlphaTensor* discovered an algorithm with $Q = 23$ and $E = 136$ without using a reward function that would optimize for numerical stability. Finally, the growth factor [11] of the factorizations is in the range from 220.14 to 547.26.

C Structured matrix multiplication

We apply *AlphaTensor* to more general operations beyond matrix multiplication. Just like standard matrix multiplication, any bilinear operation can be represented by a tensor, and its low-rank decompositions correspond to efficient algorithms. Bilinear operations encompass a large set of computational problems: structured matrix multiplication (e.g., symmetric matrix multiplication), polynomial multiplication, or more customized bilinear operations commonly used in machine learning models, such as graph networks [12, 13]. Here, we explore two use-cases: circulant matrix-vector product (or equivalently, circular convolution) in finite fields, and skew-symmetric matrix-vector product.

For the former use-case, our goal is to demonstrate the generality and power of *AlphaTensor* by discovering the *Fourier basis*, a complex and foundational transformation in mathematics, in a finite field, where arithmetic rules are non-intuitive. For the latter, the optimal algorithm is unknown [14], and we use *AlphaTensor* to discover an algorithm that improves over the state-of-the-art algorithm. We employ a common methodology for both use-cases: using *AlphaTensor*, we compute low-rank decompositions for tensors corresponding to small instances; then, following a human inspection, we generalize these algorithms to arbitrary-sized matrices/vectors.[2]

C.1 Circular matrix-vector product in finite fields

The first problem we consider is the product between a $n \times n$ circulant matrix and a vector of length n. This operation is equivalent to computing the circular convolution between two vectors of length n: the first column of the matrix and the input vector. A naive implementation of this operation would have bilinear complexity $\mathcal{O}(n^2)$; in contrast, under certain conditions, there exists an optimal algorithm that achieves bilinear complexity $\mathcal{O}(n)$. This optimal algorithm consists of three steps: (i) obtaining the discrete Fourier transform (DFT) of each vector input (in a finite field, it is the cyclotomic DFT instead), (ii) multiplying (element-wise) the results, and (iii) obtaining the inverse DFT of the result. Here we aim at rediscovering this optimal algorithm using *AlphaTensor*.

The tensor $\mathfrak{T}_n^{\text{circ}}$ that represents the circular matrix-vector product has size $n \times n \times n$, and its entries are given by $\mathfrak{T}_n^{\text{circ}}[i, j, k] = \delta\{k = (i + j) \bmod n\}$ for $0 \leq i, j, k < n$, where $\delta\{\cdot\}$ is the indicator function (we use zero-based indexing here). The tensor $\mathfrak{T}_n^{\text{circ}}$ has therefore n^2 nonzero elements, but its rank is n (provided the n-length DFT exists in the corresponding ring $\mathcal{E}$ of interest, which is always the case if $\mathcal{E} = \mathbb{R}$).

We focus on computations in finite fields. In particular, we set $\mathcal{E}$ to be the finite field of order ρ, where ρ is a prime power. This is challenging for two reasons. First, the action space becomes huge even for moderate values of n and ρ,

[2] Unlike standard matrix multiplication, where decompositions for fixed size tensors yield algorithms for any size, this extra human step can be needed for other bilinear operations where recursion is not possible or is not efficient.

e.g., for $n = 8$ and $\rho = 17$ (which are values that we consider), the action space is of the order of $\rho^{3n} \approx 10^{29}$. Second, multiplications and additions are performed according to the arithmetic in the finite field, and therefore interpreting the factor and tensor entries as integers may be a poor choice (e.g., for $\rho = 17$, the values 1 and 16 are both 1 unit away from 0, since $0 + 1 = 1$ and $16 + 1 = 0$). To account for these challenges, we adapt *AlphaTensor* as described next.

Methodology. We now describe the modifications of *AlphaTensor* that we use for this case:

- *Vector-per-move.* In the standard *TensorGame*, each move (action) corresponds to a triplet $(\mathbf{u}^{(t)}, \mathbf{v}^{(t)}, \mathbf{w}^{(t)})$. Here, we modify *TensorGame* so that the factor triplet is split into three separate moves. The first move of the game corresponds to $\mathbf{u}^{(1)}$, the second one to $\mathbf{v}^{(1)}$, the third one to $\mathbf{w}^{(1)}$, the fourth one to $\mathbf{u}^{(2)}$, and so on. Consequently, the state of the game s is augmented with the vectors already written down for the next factor: for each $t \geq 0$,

$$
\begin{aligned}
s_{3t} &= (\mathcal{S}_t, ()),\\
s_{3t+1} &= (\mathcal{S}_t, (\mathbf{u}^{(t+1)})),\\
s_{3t+2} &= (\mathcal{S}_t, (\mathbf{u}^{(t+1)}, \mathbf{v}^{(t+1)})).
\end{aligned}
$$

  The tensor $\mathcal{S}_t$ in the state is only updated after every third move, when a $\mathbf{w}^{(t)}$ vector is written down. The update is then analogous to the standard *TensorGame*. Thus, the main difference of this variant is that there are three times as many moves to reach the same residual tensor. While this has no impact on the score achieved in the game (as long as the maximum number of moves is increased threefold), there is a computational advantage: the move size is $3\times$ smaller (which implies an exponential reduction in the size of the set of possible moves), even at the expense of $3\times$ longer games.
- *Network input.* To help the network learn the arithmetic in finite fields, we pre-process the input as follows. For every input entry x, we compute all the products $\{x, 2x, \ldots, (\rho - 1)x\}$. We concatenate the $\rho - 1$ resulting entries, pre-process them using four additional dense blocks, and expose the result as an additional input to both the torso and the policy head.
- *Action canonicalization reward.* The action canonicalization is particularly important in finite fields, where a given rank-1 tensor can be expressed using $(\rho - 1)^2$ equivalent triplets $(\lambda_1\mathbf{u}, \lambda_2\mathbf{v}, \lambda_3\mathbf{w})$ with $\lambda_1\lambda_2\lambda_1 = 1 \pmod{\rho}$; e.g., there are 256 equivalent triplets for $\rho = 17$. In a finite field we define the canonical form as having the first nonzero element of $\mathbf{u}$ and the first nonzero element of $\mathbf{v}$ equal to 1. Instead of imposing the canonicalization, we add a penalty to the reward whenever the agent outputs a factor triplet that is not in canonical form. Specifically, the penalty is -0.5 for each individual factor ($\mathbf{u}$ or $\mathbf{v}$) that is not in canonical form.
- *Permutation symmetry reward.* Two actions $(\mathbf{u}, \mathbf{v}, \mathbf{w})$ and $(\mathbf{u}', \mathbf{v}', \mathbf{w}')$ can be applied in either order due to commutativity. Therefore, to reduce the number of equivalent factorizations, we use another type of canonicalization — defined over the whole factorization instead of over each individual factor. Specifically, we define the canonical representation of a factorization as the factorization in which the factors are sorted lexicographically. When the agent fails to output a factorization in its canonical form, we add a penalty reward equal to $-1/R_{\text{limit}}$ for each pair of consecutive actions that are not played in canonical order. This reward is added in addition to the factor canonicalization reward. All synthetic demonstrations are also converted to their canonical form (considering both factor canonicalization and permutation symmetry).
- *Synthetic demonstrations.* When generating demonstrations, we ensure that the factor entries take all the possible values in the finite field. We set $p_{\text{entry}}(0) = 0.5$ and $p_{\text{entry}}(v) = \frac{1}{\rho-1}$ for $v = 1, \ldots, \rho - 1$.
- *Hyper-parameters.* We set $R_{\text{limit}} = 64$ and allow all elements of $\mathcal{E}$ as possible factor entries: $F = \{0, 1, \ldots, \rho - 1\}$. The policy head uses $N_{\text{steps}} = 2$, $N_{\text{logits}} = \rho^2$ (i.e., 289 for $\rho = 17$), $N_{\text{layers}} = 4$, and at inference time (in MCTS) we sample $N_{\text{samples}} = 64$ actions. The value head is an implicit quantile network (IQN) head [15] with 8 samples, and it ignores the policy embedding input $\boldsymbol{z}_1$. Signed permutations are not used; instead we inject more diversity by setting $N_{\text{cob}} = 300{,}000$.

Results. We apply *AlphaTensor* to the tensor $\mathcal{T}_n^{\text{circ}}$, setting $\rho = 17$ and[3] $n = 2, 4, 8$, in a single-target setting (i.e., a separate experiment for each n). Extended Data Table 2 shows the obtained solutions. Despite the difficulty of the task

[3] In the finite field of order $\rho = 17$, the n-point discrete Fourier transform exists for vectors of the considered length ($n = 2, 4, 8$), as well as for the extrapolation for $n = 16$. We also ran experiments on a finite field with non-prime cardinality (with $\rho = 9$), also recovering the Fourier basis for $n = 2, 4, 8$.

and the enormous action space, *AlphaTensor* finds the optimal decomposition for the considered values of n. These decompositions correspond precisely to the Fourier basis, from which patterns can be detected through visual inspection — we color-coded such patterns for clarity in Extended Data Table 2. From these examples, one can generalize the solution to arbitrary n and field by setting $u_k^{(r)} = v_k^{(r)} = z^{kr}$ and $w_k^{(r)} = z^{-kr}/n$, where $0 \leq k, r < n$ and z is an n-th primitive root of unity[4] (see below for details on this step). That is, $\mathbf{U}$ and $\mathbf{V}$ are the (cyclotomic) DFT matrices, while $\mathbf{W}$ corresponds to the inverse DFT. As the Fourier transform is known to be the optimal algorithm, this shows the capability of *AlphaTensor* to discover optimal algorithms in huge algorithm spaces.

Generalizing the factorizations found by *AlphaTensor*. Here we exemplify the process to generalize the factorizations found by *AlphaTensor* on small tensors on the finite field of order 17 to any arbitrarily sized tensor and finite field, which is the only step in the process that requires human intervention.

We start by showing an example for $n = 4$, but the procedure is analogous for the sizes $n = 2$ and $n = 8$. For $n = 4$, the raw factorization found by *AlphaTensor* is:

$$\mathbf{U} = \mathbf{V} = \begin{pmatrix} 1 & 1 & 1 & 1 \\ 16 & 13 & 4 & 1 \\ 1 & 16 & 16 & 1 \\ 16 & 4 & 13 & 1 \end{pmatrix}, \quad \mathbf{W} = \begin{pmatrix} 13 & 13 & 13 & 13 \\ 4 & 1 & 16 & 13 \\ 13 & 4 & 4 & 13 \\ 4 & 16 & 1 & 13 \end{pmatrix},$$

where each column represents a factor, i.e., $\mathbf{U} = \mathbf{V} = [\mathbf{u}^{(1)}, \ldots, \mathbf{u}^{(n)}]$ and $\mathbf{W} = [\mathbf{w}^{(1)}, \ldots, \mathbf{w}^{(n)}]$.

First, we note that the matrices above can be made symmetric by permuting the factors (i.e., columns). This leads to the factorization[5]

$$\mathbf{U}' = \mathbf{V}' = \begin{pmatrix} 1 & 1 & 1 & 1 \\ 1 & 4 & 16 & 13 \\ 1 & 16 & 1 & 16 \\ 1 & 13 & 16 & 4 \end{pmatrix}, \quad \mathbf{W}' = 4^{-1} \cdot \begin{pmatrix} 1 & 1 & 1 & 1 \\ 1 & 13 & 16 & 4 \\ 1 & 16 & 1 & 16 \\ 1 & 4 & 16 & 13 \end{pmatrix},$$

where we have additionally re-written $\mathbf{W}'$ by taking out the factor $4^{-1} = 13$ in order to make $\mathbf{w}'^{(1)}$ similar to $\mathbf{u}'^{(1)}$.

Second, we note that the entries in the matrices above involve only a subset of the elements in $\mathcal{E}$. In particular, they involve only the elements that are powers of 4 (since $1 = 4^0$, $4 = 4^1$, $16 = 4^2$, and $13 = 4^3$). Taking this into account, and applying the identity $4^x = 4^{x+4}$ (which holds in the finite field of order $\rho = 17$), we rewrite the factors in a form that is more convenient for generalization:

$$\mathbf{U}' = \mathbf{V}' = \begin{pmatrix} 4^0 & 4^0 & 4^0 & 4^0 \\ 4^0 & 4^1 & 4^2 & 4^3 \\ 4^0 & 4^2 & 4^4 & 4^6 \\ 4^0 & 4^3 & 4^6 & 4^9 \end{pmatrix}, \quad \mathbf{W}' = 4^{-1} \cdot \begin{pmatrix} 4^0 & 4^0 & 4^0 & 4^0 \\ 4^0 & 4^{-1} & 4^{-2} & 4^{-3} \\ 4^0 & 4^{-2} & 4^{-4} & 4^{-6} \\ 4^0 & 4^{-3} & 4^{-6} & 4^{-9} \end{pmatrix},$$

which we can express in a more compact way as $u_k^{(r)} = v_k^{(r)} = 4^{kr}$ and $w_k^{(r)} = 4^{-kr}/4$ for $0 \leq k, r < n$.

Following an analogous procedure for $n = 2$, we obtain $u_k^{(r)} = v_k^{(r)} = 16^{kr}$ and $w_k^{(r)} = 16^{-kr}/2$, while for $n = 8$ we obtain $u_k^{(r)} = v_k^{(r)} = 2^{kr}$ and $w_k^{(r)} = 2^{-kr}/8$.

Thus, we can extrapolate these results to find the general expression $u_k^{(r)} = v_k^{(r)} = z^{kr}$ and $w_k^{(r)} = z^{-kr}/n$, where z is an n-th primitive root of unity in the finite field — for example, an extrapolation for $n = 16$ in the same finite field can be built using $z = 3$. Note that this generalization is valid not only for other values of n; it is also not limited to $\rho = 17$ — it is applicable to any finite field and any n such that an n-th primitive root of unity exists in the field. Furthermore, the generalization is also valid beyond finite fields, as it also applies for complex-valued inputs by setting z to an n-th primitive root of unity, i.e., $z = \exp\left\{\frac{2\pi\sqrt{-1}}{n}\right\}$.

C.2 Skew-symmetric matrix-vector product

Here, we describe the second use-case of structured matrix multiplication. We use *AlphaTensor* to discover a new algorithm for computing the product between a $n \times n$ skew-symmetric matrix $\mathbf{A}$ and a vector $\mathbf{b}$ that outperforms the state-of-the-art approaches.

[4]An n-th primitive root of unity z satisfies $z^n = 1$ and $z^k \neq 1$ for any $k \in \{1, \ldots, n-1\}$.

[5]The choice of the permutation is not unique, but any such choice leads to an analogous final expression for the generalization.

The skew-symmetric matrix-vector product is a bilinear operation that can be represented by a tensor $\mathfrak{T}_n^{\text{skew}}$ of size $\frac{n(n-1)}{2} \times n \times n$. To build this tensor, we start from the general matrix-vector multiplication tensor $\mathfrak{T}_{n,n,1}$ of size $n^2 \times n \times n$. We build the tensor $\mathfrak{T}_n^{\text{skew}}$ as follows: first we compute an auxiliary tensor $\mathfrak{T}_n^{\text{aux}}[i,j,k] = \mathfrak{T}_{n,n,1}[i,j,k] - \mathfrak{T}_{n,n,1}[i,k,j]$, and then we remove all the slices of the auxiliary tensor that are linearly dependent, obtaining the $\frac{n(n-1)}{2} \times n \times n$ tensor $\mathfrak{T}_n^{\text{skew}}$. An explicit algorithm to build this tensor is presented in Algorithm C.14.

Algorithm C.14: Builds tensor representing the skew-symmetric matrix-vector multiplication of size n.

Input: n (the size of both the matrix and the vector).

```
m = n(n − 1)/2
T^skew = 0
r = 1
c = 1
for i ∈ {1, . . . , m} do
    c = c + 1
    if (c − 1) mod n = 0 then
        r = r + 1
        c = r + 1
    T^skew[i, r, c] = 1
    T^skew[i, c, r] = −1
return T^skew
```

Results. Figure 4a in the main paper shows the decompositions obtained by *AlphaTensor*. Similarly to the previous use-case of structured matrix multiplication (Appendix C.1), we detect a pattern for small sizes n (color-coded in the illustration), which we generalize and prove for arbitrary n, yielding a general algorithm for the skew-symmetric matrix-vector product (Figure 4b in main paper).

Theorem C.4. *The number of multiplications required to multiply a skew-symmetric matrix and a vector of dimension n is at most $(n-1)(n+2)/2$.*

Proof. See Section I.3. □

This algorithm is strictly better in terms of bilinear complexity compared to previously known algorithms [14] — it uses $\sim \frac{1}{2}n^2$ multiplications instead of $\sim n^2$. We also note that this discovered algorithm is asymptotically optimal, as $\frac{1}{2}n^2$ matches the asymptotic lower bound obtained by counting the number of different elements in a skew-symmetric matrix. The discovered algorithm, which improves upon state-of-the-art methods, demonstrates the viability of *AlphaTensor* in discovering new and more efficient algorithms. We believe this methodology can be applied to other bilinear operations, and yield efficient algorithms taking into account the structure of the problem.

Generalizing the factorizations found by *AlphaTensor*. Unlike the circulant matrix-vector case, in skew-symmetric matrix-vector multiplication there exist many decompositions of the same rank, even when factoring out permutation and factor symmetries. *AlphaTensor* indeed found many such valid decompositions that are not just the ones presented in Figure 4a. Nevertheless, we noted that many of these decompositions presented a peculiar structure: for each factor, either $\mathbf{u}$ or $\mathbf{v}$ is a one-hot vector. Even more interestingly, when $\mathbf{u}$ is a one-hot vector whose nonzero entry corresponds to element (i,j) from the matrix $\mathbf{A}$, then the corresponding vector $\mathbf{v}$ is non-zero only in the elements i and j. Conversely, when $\mathbf{v}$ is a one-hot vector whose nonzero entry corresponds to the i-th element of the vector $\mathbf{b}$, then the corresponding $\mathbf{u}$ is non-zero only on the elements corresponding to (j,i) of matrix $\mathbf{A}$ for any j in $\{1,\ldots,n\}$ (i.e., the elements of the matrix that get multiplied by the i-th element of the vector $\mathbf{b}$). This insight led us to the hypothesis that we can always decompose the n-skew-symmetric matrix-vector multiplication tensor by using $(n+2)(n-1)/2$ terms of the special forms described above. This is the strategy followed in the proof in Section I.3, which shows that this is indeed the case.

D Rapid tailored algorithm discovery

D.1 Benchmarking details

We use *AlphaTensor* to find provably correct matrix multiplication algorithms which are tailored to optimize the running time in a particular setting, where the setting includes hardware (e.g., a V100 GPU or TPU), matrix size (e.g., multiplying a pair of $8{,}192 \times 8{,}192$ matrices), and the arithmetic precision (e.g., single precision arithmetic). In these experiments we benchmark algorithms discovered by *AlphaTensor* on the fly, and use this benchmarking result for the reward function. That is, we set $r'_t = r_t + \lambda b_t$, where r_t is the rank reward described in the paper, b_t is the benchmarking reward (nonzero only at the terminal state), and λ is a user-specified coefficient.[6]

After every game that ends with a correct factorization of the matrix multiplication tensor, an *AlphaTensor* actor sends a remote request to a benchmarking server equipped with the target hardware (e.g., a V100 GPU). The benchmarking server builds the matrix multiplication algorithm defined by the incoming factorization and then applies just-in-time (JIT) compilation using JAX [16], a Python library for high-performance machine learning research. JAX JIT analyzes the given Python code and optimizes the code for the target hardware. For example, it can decide to fuse a matrix multiplication followed by a matrix addition into a single CUDA GEMM call. JAX also controls the order of execution of operations and can schedule multiple ops to run in parallel. In many cases it is possible to outperform JAX JIT by writing custom CUDA code, but JAX JIT proved to be good enough in our experiments, with the additional benefit of being fully automatic. This allowed us to automatically optimize and benchmark every matrix multiplication algorithm proposed by *AlphaTensor*.

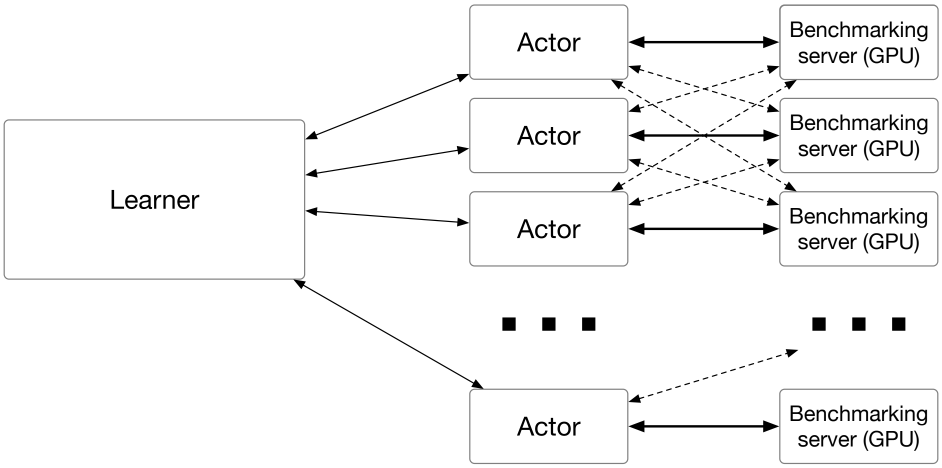

Figure D.1: Schematic illustration of the training pipeline when using benchmarking to define the reward function. Every actor has a dedicated benchmarking server, but also occasionally uses other benchmarking servers (indicated by dashed lines) to increase the precision for the most promising algorithms.

To implement the benchmarking, we were guided by the information in [17], as well as the Python `timeit` module documentation [18]. To reduce benchmarking noise, `timeit` employs three tricks: it runs the function being benchmarked many times (such that the overall running time of the loop is at least 0.2 seconds); it disables Python garbage collection for the duration of benchmarking; and it repeats the loop three times, reporting the minimal timing (to be more robust to spikes in the timing due to interruptions from the OS and other users of the machine).

We followed all these three practices with a single modification. When calling a JAX JIT function, it returns a future instead of the immediate result, scheduling the computation on the GPU. As a result, running the matrix multiplication function multiple times in a loop can potentially schedule the function multiple times and potentially run them in parallel, which can skew the benchmarking results. We therefore opted to compute a sequence of matrix multiplications that all depend on each other in the inner loop, namely six sequential matrix multiplications $(\mathbf{A}(\mathbf{A}(\mathbf{A}(\mathbf{A}(\mathbf{A}(\mathbf{AB}))))))$ and used the `block_until_ready()` method on the final output. In this way, the matrix multiplications in the inner loop run sequentially, but the GPU is never blocked by waiting for the CPU to schedule the next multiplication, since

[6] While the benchmarking reward might be enough in theory, it is useful for the learning dynamics to combine benchmarking with rank reward, to shape the reward towards more efficient algorithms.

scheduling the next multiplication by the CPU can be done in parallel with computing the current multiplication by the GPU.

When implementing this approach, we found that the variance of benchmarking was still large. For example, when using this approach to benchmark an algorithm discovered by *AlphaTensor*, the estimated speed up against the baseline Strassen^2 algorithm varied from 4% to 11.6% (see Figure D.2). We identified the main reason of this noise to be the GPU frequency scaling (GPU driver can change the clock frequency on the fly). We implemented two additional techniques to improve the stability of the measurements.

First, additionally to measuring the running time of the algorithm of interest, we also measure the running time of the baseline Strassen^2 algorithm and report the ratio between the two. The idea is that since the two measurements are done one after another, the clock frequency is likely to be similar when benchmarking the two algorithms. This makes our rewards comparable even for two factorizations benchmarked hours apart using very different clock frequencies, since each ratio represents the speedup with respect to the baseline computed with (almost) fixed frequency. We repeat this process of benchmarking the target algorithm and the baseline several times (4 in the experiments) and report the median ratio. As seen from numerical experiments, using this additional trick indeed significantly reduces the variance of the measurements (see Figure D.2).

Second, to be even more agnostic to the state of the benchmarking server, we benchmark the most promising algorithms on multiple benchmarking servers and report the median across those. Namely, for algorithms that are better than 75% of the other algorithms seen so far we use the median ratio across 2 benchmarking servers, for algorithms that are better than 90% of the algorithms seen so far we use the median across 5 servers, and for algorithms that are better than 95% of the other algorithms we use the median across 17 servers.

When measuring the running time of an algorithm, we preload the input data (the two block matrices) into the GPU memory and do a series of 3 warm-up runs to make sure the GPU is ready to be used for benchmarking. We do not count the time to transfer the data to and from the GPU memory, as in most cases the GPU is used for more than one matrix multiplication operation in a row and the data transfer time gets amortized. We also do not count the time to split the input matrices into blocks and to construct the resulting matrix from the blocks as it is possible to implement any of the fast matrix multiplication algorithm in C CUDA using memory addressing which would make the block splitting and concatenating essentially free. See benchmarking pseudocode in Figure D.3.

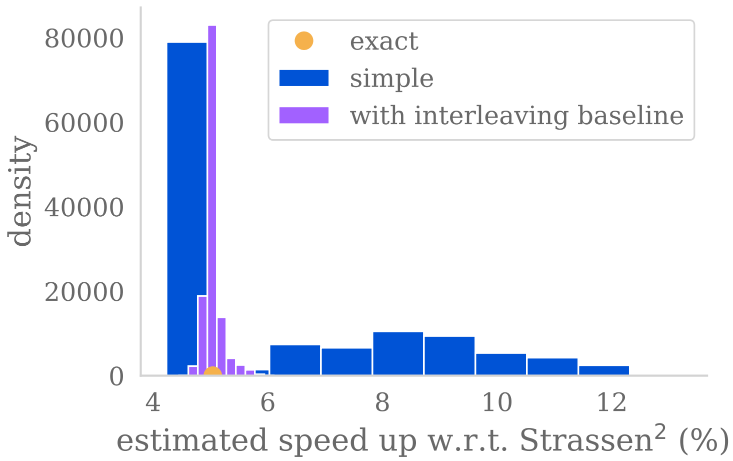

Figure D.2: Estimated speed up of a single algorithm with respect to the Strassen^2 baseline. The histogram is computed by repeating the same benchmark $1{,}000$ times on 100 different GPUs ($100{,}000$ benchmarkings in total). The two plots correspond to the two different benchmarking schemes discussed in the text: a "simple" scheme based on the Python best practices and "with interleaving baseline" that includes the additional trick discussed above to circumvent the frequency scaling issue. The "exact" speed up is computed as the median of all the speed ups estimated by both methods.

In the *AlphaTensor* runs, we use Strassen^2 as the baseline algorithm for the benchmarking. However, to report the results, our discovered algorithms are re-benchmarked against standard matrix multiplication, where we use a higher precision. Namely, we used the median ratio across 200 repeats. To further increase the accuracy, we fixed the GPU

```python
def benchmark_single_server(factorization , a, b):
    algorithm = algorithm_from_factors(factorization)
    algorithm = jax.jit(algorithm)
    baseline = jax.jit(algorithm_from_factors(strassen_factorization))

    # Warm-up.
    for i in range(3):
        algorithm(a, b).block_until_ready()
        baseline(a, b).block_until_ready()

    gc.disable()  # Disable the garbage collector.
    ratios = []
    for i in range(4):
        start = time.time()
        c = b
        for j in range(6):
            c = algorithm(a, c)
        c.block_until_ready()
        end = time.time()
        alg_time = end - start

        start = time.time()
        c = b
        for j in range(6):
            c = baseline(a, c)
        c.block_until_ready()
        end = time.time()
        baseline_time = end - start

        ratios.append(baseline_time / alg_time)
    gc.enable()
    return np.median(ratios)
```

Figure D.3: Python pseudocode for benchmarking a matrix multiplication algorithm on a single benchmarking server (as discussed in Appendix D.1, for the most promising factorizations we additionally compute the median ratio across multiple servers).

clock frequency[7] to the maximum possible value (1530 for a V100 GPU). For example, when repeatedly estimating the speed up of Strassen[2] against the standard baseline using this approach, we got estimates from 4.15% to 4.3% with a standard deviation of 0.06 percent points.

For TPUs we follow the exact same benchmarking procedures, with only two exceptions: a) we don't fix the clock frequency (because on TPUs clock is always constant); b) we use matrices of data type `bfloat16` (in contrast to `float32` used for GPUs), as this is a more common setting among TPU users.

D.2 Experimental setup

We adapt *AlphaTensor* to work with benchmarking reward as follows. The immediate reward is set to $r'_t = r_t + \lambda b_t$, where r_t is the reward described in the paper, and b_t is the benchmarking reward, equal to zero in all intermediate states, and equal to

$$b_t = \frac{\text{time taken to execute baseline algorithm}}{\text{time taken to execute found algorithm}} - \gamma,$$

for a terminal state with $\mathcal{S}_t = \mathbf{0}$, where γ is a hyper-parameter. As the algorithms found initially are of high rank (and hence not efficient), we initially set $\lambda = 0$, and activate the benchmarking reward only in late stages of the training (i.e., when *AlphaTensor* discovers sufficiently small ranks). In our experiments we used $\lambda = 5.0$, $\gamma = 0.8$ and Strassen[2] as the baseline algorithm. For synthetic demonstrations, the benchmarking reward is set to 0, as such tensors do not correspond to a matrix multiplication algorithm. Moreover, we only benchmark games played in the canonical basis, as we did not find the use of other basis helpful.

In addition to the value head that predicts the rank of the tensor, we train an auxiliary value head and an auxiliary reward head. The auxiliary reward head is trained to predict b_t, while the auxiliary value head is trained to predict the auxiliary return (future auxiliary reward) at any state. The value that is used in MCTS is a linear combination of the

[7] We only fix the GPU clock frequency for the final reporting because our internal infrastructure makes it hard to do it for live experiments at scale.

main value and the auxiliary value, $v = v_{\text{main}} + \lambda v_{\text{auxiliary}}$. We use the rewards predicted by the reward head in MCTS simulations, as benchmarking an algorithm to compute the true environment reward b_t is time consuming, and would significantly slow down MCTS. These auxiliary heads are only trained on selfplay games, i.e. we do not train them on demonstrations.

In addition to the usual inputs (described in Methods section) we provide an extra input to the neural network consisting of all previous actions (factors) played to reach the current state (tensor). Unlike the residual tensor that does not contain information about the matrix multiplication algorithm, the factors encode the algorithm and hence provide important information to the auxiliary heads.

D.3 Additional results

We now show that by optimizing algorithms for larger sizes, one can obtain further speed-ups. Our experiments are performed on TPU v2. We note a substantial speed-up when optimizing the algorithm for the intended size. For example, when multiplying matrices of size $16{,}384$, the algorithm optimized for $8{,}192$ yields 11.2% speed-up compared to the standard algorithm, while the *AlphaTensor*-discovered algorithm optimized for matrices of size $16{,}384$ achieves a 13.5% speed-up.

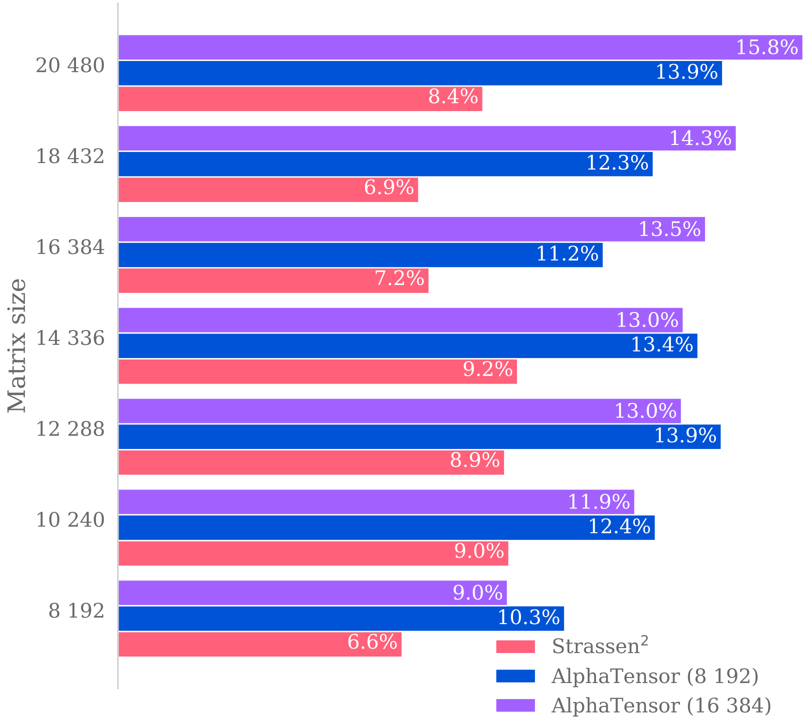

Figure D.4: Speed-up of two algorithms found by *AlphaTensor*, relative to the standard (optimized) matrix multiplication algorithm on TPU v2. The first algorithm (shown in blue) is optimized for multiplying matrices of size $8{,}192$, while the second (shown in purple) is optimized for multiplying matrices of size $16{,}384$. The baseline Strassen^2 algorithm is also shown. We report the median over $1{,}000$ runs. The standard deviation over runs is < 0.4 percent points.

E Finding border rank with *AlphaTensor*

AlphaTensor is a general method that supports any tensor decomposition environment and any reward function. One example is *AlphaTensor*'s ability to operate in finite fields, where the factor and tensor entries live in a finite set, and the multiplication and addition arithmetic obeys the rules in finite fields. Another example is *AlphaTensor*'s ability to find efficient algorithms tailored to specific hardware.

To demonstrate the ability of *AlphaTensor* to decompose tensors in different settings, we show a use-case that departs from the standard low-rank tensor decomposition. Specifically, we aim at finding the *border rank* of a tensor. The border rank of a tensor $\mathcal{T}$ is the minimum integer R such that the decomposition $\sum_{r=1}^{R} \mathbf{u}^{(r)}(\varepsilon) \otimes \mathbf{v}^{(r)}(\varepsilon) \otimes \mathbf{w}^{(r)}(\varepsilon)$ converges to $\mathcal{T}$ as the scalar $\varepsilon \to 0$ for some factors $\{\mathbf{u}^{(r)}(\varepsilon), \mathbf{v}^{(r)}(\varepsilon), \mathbf{w}^{(r)}(\varepsilon)\}$ that depend on ε. We emphasize that the goal of this section is to showcase the flexibility of *AlphaTensor*, as opposed to discovering new results.

Methodology. To find the border rank of a tensor, we modify *AlphaTensor* and *TensorGame* as follows.

- *Ring.* We set the ring $\mathcal{E}$ to be the set of Laurent polynomials in ε. Thus, the factor entries are Laurent polynomials in the variable ε, which we constrain to be in the set $F = \{0, \pm 1, \pm\varepsilon^i, \pm\varepsilon^{2i}, \pm 1 \pm \varepsilon^i, \pm 1 \pm \varepsilon^{2i}, \pm\varepsilon^i \pm \varepsilon^{2i}, \pm\varepsilon^i \pm \varepsilon^{-i}, \pm\varepsilon^i \pm \varepsilon^{-2i}, \pm\varepsilon^{2i} \pm \varepsilon^{-2i}\}_{i=\pm 1}$, i.e., there are $|F| = 51$ possible entries. The addition and multiplication operations are performed according to the standard polynomial arithmetic; that is, the entries of the state tensor $\mathcal{S}_t$ are Laurent polynomials that are not generally in the set F.
- *Disallowed actions.* We disallow actions (factor triplets) $\mathbf{u}^{(r)}(\varepsilon) \otimes \mathbf{v}^{(r)}(\varepsilon) \otimes \mathbf{w}^{(r)}(\varepsilon)$ that lead to either the all-zero tensor or a rank-one tensor whose *all* entries are $\mathcal{O}(\varepsilon)$, since these actions do not make any progress towards finding a border rank decomposition. Moreover, for simplicity we also disallow actions that lead to a rank-one tensor containing any Laurent polynomial with any term of degree strictly lesser than -3.
- *Game end and reward.* We terminate the game at step R if either all the terms of the residual tensor $\mathcal{T} - \sum_{r=1}^{R} \mathbf{u}^{(r)}(\varepsilon) \otimes \mathbf{v}^{(r)}(\varepsilon) \otimes \mathbf{w}^{(r)}(\varepsilon)$ are $\mathcal{O}(\varepsilon)$, or $R = R_{\text{limit}}$. The reward is -1 for each step taken during the game, plus an additional negative reward equal to the number of terms of the residual tensor that are not $\mathcal{O}(\varepsilon)$.
- *Network input.* To form the input of the network at step t, we concatenate 4 tensors obtained from $\mathcal{S}_t$. These 4 tensors contain integer-valued entries instead of Laurent polynomials. To form them, we retain the coefficients of $\mathcal{S}_t$ corresponding to ε^d for some fixed value of d. By letting the exponent d take values in $\{-3, -2, -1, 0\}$, we obtain the 4 tensors that we concatenate together (the coefficients corresponding to positive degrees d do not have any relevance when $\varepsilon \to 0$, so we ignore them).
- *Action canonicalization.* We canonicalize the factor triplets similarly as the standard *AlphaTensor*. We define the canonical form of $\mathbf{u}(\varepsilon)$ as the factor $\mathbf{u}'(\varepsilon) = \lambda \mathbf{u}(\varepsilon)$ (with $\lambda \in \{-1, +1\}$) such that the Laurent polynomial corresponding to the first nonzero entry of $\mathbf{u}'(\varepsilon)$ has positive coefficient for the term with smallest degree (e.g., the first nonzero entry is $\varepsilon^{-1} - 1$ instead of $-\varepsilon^{-1} + 1$). Moreover, whenever all the entries of a factor triplet $(\mathbf{u}(\varepsilon), \mathbf{v}(\varepsilon), \mathbf{w}(\varepsilon))$ are polynomials with non-negative exponents of ε, we canonicalize the triplet by setting $\varepsilon = 0$.
- *Synthetic demonstrations.* To generate synthetic demonstrations, we follow the same approach as when optimizing the standard rank: we sample random factors $(\mathbf{u}^{(r)}(\varepsilon), \mathbf{v}^{(r)}(\varepsilon), \mathbf{w}^{(r)}(\varepsilon))$, and then create the tensor $\mathcal{T}(\epsilon) = \sum_{r=1}^{R} \mathbf{u}^{(r)}(\varepsilon) \otimes \mathbf{v}^{(r)}(\varepsilon) \otimes \mathbf{w}^{(r)}(\varepsilon)$, so that the demonstration is $(\{(\mathbf{u}^{(r)}(\varepsilon), \mathbf{v}^{(r)}(\varepsilon), \mathbf{w}^{(r)}(\varepsilon))\}_{r=1}^{R}, \mathcal{T}(\epsilon))$. To generate each factor, we independently sample its entries. For each entry, we generate a Laurent polynomial with either one or two non-zero coefficients, with probabilities 0.7 and 0.3, respectively (each coefficient can be either 1 or -1 with equal probability). We assign each coefficient to a term ε^d of the polynomial, where d itself is a random variable with $p(d = 0) = 0.7$, $p(d = 1) = p(d = -1) = 0.1$, and $p(d = 2) = p(d = -2) = 0.05$. We reject those factor triplets that do not conform valid actions.
- *Change of basis generation.* We generate change of basis similarly as the standard *AlphaTensor*. The change of basis matrices contain entries in $\{-1, 0, 1\}$, with probabilities $p_{\text{cob-entry}}(0) = 0.96$ and $p_{\text{cob-entry}}(-1) = p_{\text{cob-entry}}(1) = 0.02$.
- *Hyper-parameters.* We set $R_{\text{limit}} = 12$. The policy head uses $N_{\text{steps}} = 2$ and $N_{\text{logits}} = |F|^2 = 2{,}601$. We set $N_{\text{cob}} = 10{,}000$.

Results. We apply *AlphaTensor* to find the border rank decomposition of three tensors: the $2 \times 2 \times 2$ tensor $\mathcal{T}^{\text{small}}$ that is zero everywhere except for $\mathcal{T}^{\text{small}}[1,1,2] = \mathcal{T}^{\text{small}}[1,2,1] = \mathcal{T}^{\text{small}}[2,1,1] = 1$ (see [19]); the $4 \times 4 \times 4$ tensor $\mathcal{T}^{\text{Bini}}$ obtained from $\mathcal{T}_2$ after replacing one unit entry with zero, as in [20]; and the $4 \times 6 \times 6$ matrix multiplication tensor $\mathcal{T}_{2,2,3}$. The rank of $\mathcal{T}^{\text{small}}$, $\mathcal{T}^{\text{Bini}}$, and $\mathcal{T}_{2,2,3}$ is 3, 6, and 11, respectively, while the border rank is 2, 5, and 10 [19, 21, 22]. *AlphaTensor* successfully finds $R = 2$, $R = 5$, and $R = 10$ for each target respectively; in fact for each one it discovers multiple decompositions with R factors. This demonstrates *AlphaTensor*'s ability to operate in a complex environment with more general game state and actions.

For completeness, we show next one of the border-rank-5 decompositions of $\mathcal{T}^{\text{Bini}}$ found by *AlphaTensor*:

$$\mathbf{U}(\varepsilon) = \begin{pmatrix} 1 & 1 & 1 & 1 & 1-\varepsilon \\ 0 & 1 & 0 & 0 & -\varepsilon \\ 0 & 0 & -\varepsilon^2 & 0 & 0 \\ 0 & 0 & 0 & \varepsilon^2 & \varepsilon^2 \end{pmatrix}, \quad \mathbf{V}(\varepsilon) = \begin{pmatrix} 1 & 0 & 1 & 0 & 0 \\ 0 & 0 & 0 & 0 & \varepsilon \\ -1 & \varepsilon^{-1} & 0 & \varepsilon^{-2} & \varepsilon^{-1} \\ 0 & 1 & 0 & 0 & -\varepsilon \end{pmatrix},$$

$$\mathbf{W}(\varepsilon) = \begin{pmatrix} 1 & \varepsilon & 0 & 0 & 0 \\ \varepsilon^{-2} & 0 & -\varepsilon^{-2} & 1 & 0 \\ 0 & 1 & 0 & -1 & \varepsilon^{-1} \\ 0 & 0 & \varepsilon & 0 & 0 \end{pmatrix},$$

where the factors are stacked vertically, e.g., $\mathbf{U}(\varepsilon) = [\mathbf{u}^{(1)}(\varepsilon), \ldots, \mathbf{u}^{(R)}(\varepsilon)]$.

Similarly, we show next a border-rank-10 decomposition of $\mathcal{T}_{2,2,3}$ found by *AlphaTensor*:

$$\mathbf{U}(\varepsilon) = \begin{pmatrix} 1 & \varepsilon^{-2}+1 & 0 & 1 & \varepsilon^{-2}+1 & 0 & 0 & \varepsilon & 0 & \varepsilon^{-1}+1 \\ 0 & 0 & 0 & -1 & 0 & 0 & \varepsilon & 0 & 1 & -1 \\ 0 & 1 & 1 & 0 & 0 & 1 & \varepsilon^{-1} & -\varepsilon^{-1} & \varepsilon^{-2}+\varepsilon^{-1} & 0 \\ 0 & 0 & 0 & 0 & 1 & 1 & 0 & 0 & \varepsilon^{-1} & \varepsilon \end{pmatrix},$$

$$\mathbf{V}(\varepsilon) = \begin{pmatrix} 1 & 1 & 0 & 0 & 0 & 0 & 0 & 0 & 0 & 0 \\ 0 & -\varepsilon^2 & 1 & 0 & \varepsilon^2 & 0 & 0 & 1 & 0 & \varepsilon \\ 0 & 0 & 0 & 0 & 0 & 0 & 0 & 0 & \varepsilon^2 & \varepsilon \\ 1 & 0 & 0 & \varepsilon^{-1} & 1 & 0 & 0 & 0 & 0 & \varepsilon^{-1} \\ 0 & 0 & -1 & 0 & 0 & \varepsilon^{-1} & \varepsilon^{-1} & 0 & 1 & 0 \\ 0 & 0 & 0 & 1 & 0 & -1 & 0 & 0 & -\varepsilon^2 & \varepsilon \end{pmatrix},$$

$$\mathbf{W}(\varepsilon) = \begin{pmatrix} 1 & 0 & 0 & -\varepsilon & 0 & 0 & 0 & 0 & 0 & 0 \\ -\varepsilon^{-2}-1 & 1 & 0 & 0 & 1+\varepsilon^2 & 0 & 0 & 0 & 0 & -\varepsilon^2 \\ 0 & 0 & \varepsilon^{-2} & 0 & 0 & 0 & 1 & \varepsilon^{-1} & 0 & 0 \\ 0 & 0 & 1 & 0 & 0 & \varepsilon & 0 & 0 & 0 & 0 \\ 1 & -\varepsilon^2 & 0 & -1+\varepsilon^2 & -1 & 0 & 0 & 0 & 0 & 1 \\ 0 & 0 & 0 & 0 & \varepsilon^2 & -1 & -1 & 0 & 1 & -\varepsilon^2 \end{pmatrix}.$$

F Ablations

Ablation	Rank found
Without *synthetic demonstrations*	64
Without *selfplay* (supervised only)	60
Without *change of basis*	58
Without *signed permutations*	53
Without retraining on best games 10% of the time	52
Without *QR head* (using a categorical head)	52
AlphaTensor (no ablation)	49

Table 1: Results of repeating the experiment that discovered rank-49 factorizations of the general matrix multiplication tensor $\mathcal{T}_4$ (over standard arithmetic), each time disabling one of the components of *AlphaTensor*. In the experiment without selfplay we trained *AlphaTensor* on synthetic demonstrations only, and used the resulting neural network in MCTS seeking to decompose the target tensor $\mathcal{T}_4$.

Apart from the components ablated in Table 1, another important feature of *AlphaTensor* is the ability to train a single agent for decomposing multiple target tensors. When working over modular arithmetic $\mathbb{Z}_2$, an agent trained on all matrix multiplication tensors $\mathcal{T}_{n,m,p}$ with $n, m, p \leq 5$ discovers a rank-47 factorization of $\mathcal{T}_4$, whereas an agent trained solely on $\mathcal{T}_4$ only discovers rank-49 factorizations. We performed an analysis on one of the experiments that discovered a rank-47 factorization of $\mathcal{T}_4$ and found that the agent is able to transfer knowledge between the three target tensors $\mathcal{T}_{3,3,4}$, $\mathcal{T}_{3,4,4}$, and $\mathcal{T}_4$. Specifically, a new agent trained from scratch but initialized with the discovered solutions to

both $\mathcal{T}_{3,3,4}$ and $\mathcal{T}_{3,4,4}$ in its demonstrations buffer is very quickly able to rediscover rank-47 factorizations of $\mathcal{T}_4$. This is not the case when the agent is initialized with solutions to $\mathcal{T}_{3,3,4}$ only, or with solutions to $\mathcal{T}_{3,4,4}$ only.

G Hyperparameters

	Hyper-parameter	Value
Game	Move limit R_{limit}	125
	Factor entries F when seeking algorithms over arbitrary rings	$\{-2, -1, 0, 1, 2\}$
	Factor entries F when seeking algorithms over $\mathbb{Z}_2$	$\{0, 1\}$
MCTS	Number of simulations (before 50k steps)	200
	Number of simulations (after 50k steps)	800
	Initial value of $c(s)$ (c_1 in [23])	1.25
	Scaling of $c(s)$ with parent visit count (c_2 in [23])	19,652
RL pipeline	Maximum number of times a state is trained on	1
	Replay buffer size (selfplay games)	100,000
	Synthetic demonstrations	5,000,000
	Best games replay buffer size	1,000
	Training split (selfplay / demonstrations / best games) before 10k steps	10% / 90% / 0%
	Training split (selfplay / demonstrations / best games) after 10k steps	70% / 25% / 5%
Optimization	Batch size	2048
	Gradient clipping by global norm	4.0
	Optimizer	AdamW [24]
	Weight decay	10^{-5}
	Initial learning rate	10^{-4}
	Learning rate decay factor	0.1
	Learning rate decay steps	500,000

Table 2: Common training hyper-parameters.

The hyper-parameters for specific sub-components are listed where those components are described: the configuration of the network architecture in Appendix A.1, the synthetic demonstration generation procedure in Appendix A.2, and the distribution and sampling of basis changes in Appendix A.3. Table 2 lists the remaining general hyper-parameters.

H Combining smaller factorizations into bigger ones

In this section, we describe and extend the divide-and-conquer technique [25, 26] that allows one to combine factorizations of smaller matrix multiplication tensors into factorizations of larger matrix multiplication tensors. For example, this technique allows us to obtain a rank-287 factorization of $\mathcal{T}_{5,8,10}$ (which improves over the previously known rank-291 [25]) by combining a known rank-33 factorization of $\mathcal{T}_{2,4,5}$ and a rank-47 factorization of $\mathcal{T}_{3,4,5}$ found by *AlphaTensor*. We refer to the supplementary data for all such decompositions.

H.1 Illustrative example

Let us describe the divide-and-conquer approach using $\mathcal{T}_9$ as an illustrative example. Specifically, let us find an algorithm for multiplying two 9×9 matrices $\mathbf{A}$ and $\mathbf{B}$,

$$\underbrace{\begin{pmatrix} a_{11} & a_{12} & a_{13} & a_{14} & a_{15} & a_{16} & a_{17} & a_{18} & a_{19} \\ a_{21} & a_{22} & a_{23} & a_{24} & a_{25} & a_{26} & a_{27} & a_{28} & a_{29} \\ a_{31} & a_{32} & a_{33} & a_{34} & a_{35} & a_{36} & a_{37} & a_{38} & a_{39} \\ a_{41} & a_{42} & a_{43} & a_{44} & a_{45} & a_{46} & a_{47} & a_{48} & a_{49} \\ a_{51} & a_{52} & a_{53} & a_{54} & a_{55} & a_{56} & a_{57} & a_{58} & a_{59} \\ a_{61} & a_{62} & a_{63} & a_{64} & a_{65} & a_{66} & a_{67} & a_{68} & a_{69} \\ a_{71} & a_{72} & a_{73} & a_{74} & a_{75} & a_{76} & a_{77} & a_{78} & a_{79} \\ a_{81} & a_{82} & a_{83} & a_{84} & a_{85} & a_{86} & a_{87} & a_{88} & a_{89} \\ a_{91} & a_{92} & a_{93} & a_{94} & a_{95} & a_{96} & a_{97} & a_{98} & a_{99} \end{pmatrix}}_{\mathbf{A}} \cdot \underbrace{\begin{pmatrix} b_{11} & b_{12} & b_{13} & b_{14} & b_{15} & b_{16} & b_{17} & b_{18} & b_{19} \\ b_{21} & b_{22} & b_{23} & b_{24} & b_{25} & b_{26} & b_{27} & b_{28} & b_{29} \\ b_{31} & b_{32} & b_{33} & b_{34} & b_{35} & b_{36} & b_{37} & b_{38} & b_{39} \\ b_{41} & b_{42} & b_{43} & b_{44} & b_{45} & b_{46} & b_{47} & b_{48} & b_{49} \\ b_{51} & b_{52} & b_{53} & b_{54} & b_{55} & b_{56} & b_{57} & b_{58} & b_{59} \\ b_{61} & b_{62} & b_{63} & b_{64} & b_{65} & b_{66} & b_{67} & b_{68} & b_{69} \\ b_{71} & b_{72} & b_{73} & b_{74} & b_{75} & b_{76} & b_{77} & b_{78} & b_{79} \\ b_{81} & b_{82} & b_{83} & b_{84} & b_{85} & b_{86} & b_{87} & b_{88} & b_{89} \\ b_{91} & b_{92} & b_{93} & b_{94} & b_{95} & b_{96} & b_{97} & b_{98} & b_{99} \end{pmatrix}}_{\mathbf{B}}.$$

To apply the divide-and-conquer approach, we need a *high-level matrix multiplication algorithm*, *i.e.,* a factorization of some matrix multiplication tensor. The choice of the tensor and its factorization affects the resulting number of factors of the decomposition of $\mathcal{T}_9$. Hence, in practice, it is beneficial to consider all available choices (i.e., all available sizes and algorithms), and pick the one leading to the best result. To make the exposition concrete, let us consider $\mathcal{T}_{2,3,3}$. (This is a generalization of the scheme described in [25], which only considered factorizations of $\mathcal{T}_2$ as the high-level matrix multiplication algorithm.) Consider the following high-level algorithm representing a rank-15 decomposition of $\mathcal{T}_{2,3,3}$ (this particular decomposition is discovered by *AlphaTensor*):

$$\begin{aligned}
\mathbf{h}_1 &= \mathbf{A}_{23}(\mathbf{B}_{21} - \mathbf{B}_{23} - \mathbf{B}_{31}) \\
\mathbf{h}_2 &= (\mathbf{A}_{22} + \mathbf{A}_{23})(\mathbf{B}_{21} - \mathbf{B}_{23}) \\
\mathbf{h}_3 &= (\mathbf{A}_{12} - \mathbf{A}_{23})(\mathbf{B}_{21} - \mathbf{B}_{23} - \mathbf{B}_{32}) \\
\mathbf{h}_4 &= (\mathbf{A}_{12} + \mathbf{A}_{22})(\mathbf{B}_{12} + \mathbf{B}_{21} - \mathbf{B}_{22} - \mathbf{B}_{23}) \\
\mathbf{h}_5 &= \mathbf{A}_{12}(\mathbf{B}_{22} + \mathbf{B}_{23} - \mathbf{B}_{32}) \\
\mathbf{h}_6 &= (\mathbf{A}_{12} + \mathbf{A}_{13})\mathbf{B}_{32} \\
\mathbf{h}_7 &= (\mathbf{A}_{12} + \mathbf{A}_{21} + \mathbf{A}_{22})(\mathbf{B}_{12} + \mathbf{B}_{23}) \\
\mathbf{h}_8 &= (\mathbf{A}_{21} + \mathbf{A}_{22})\mathbf{B}_{23} \\
\mathbf{h}_9 &= (\mathbf{A}_{11} + \mathbf{A}_{12} + \mathbf{A}_{21} + \mathbf{A}_{22})\mathbf{B}_{12} \\
\mathbf{h}_{10} &= (\mathbf{A}_{13} + \mathbf{A}_{23})(\mathbf{B}_{11} - \mathbf{B}_{13} + \mathbf{B}_{31} - \mathbf{B}_{32} - \mathbf{B}_{33}) \\
\mathbf{h}_{11} &= (\mathbf{A}_{11} - \mathbf{A}_{13} - \mathbf{A}_{23})(\mathbf{B}_{11} - \mathbf{B}_{13} - \mathbf{B}_{33}) \\
\mathbf{h}_{12} &= (\mathbf{A}_{11} - \mathbf{A}_{13} + \mathbf{A}_{21} - \mathbf{A}_{23})(\mathbf{B}_{11} - \mathbf{B}_{13}) \\
\mathbf{h}_{13} &= (\mathbf{A}_{11} - \mathbf{A}_{13})\mathbf{B}_{33} \\
\mathbf{h}_{14} &= \mathbf{A}_{21}(\mathbf{B}_{11} - \mathbf{B}_{23}) \\
\mathbf{h}_{15} &= \mathbf{A}_{11}(\mathbf{B}_{12} + \mathbf{B}_{13} + \mathbf{B}_{33}) \\
\mathbf{C}_{11} &= \mathbf{h}_{10} + \mathbf{h}_{11} + \mathbf{h}_{15} + \mathbf{h}_1 + \mathbf{h}_3 + \mathbf{h}_6 + \mathbf{h}_7 - \mathbf{h}_8 - \mathbf{h}_9 \\
\mathbf{C}_{21} &= \mathbf{h}_{14} - \mathbf{h}_1 + \mathbf{h}_2 + \mathbf{h}_8 \\
\mathbf{C}_{12} &= \mathbf{h}_5 + \mathbf{h}_6 - \mathbf{h}_7 + \mathbf{h}_8 + \mathbf{h}_9 \\
\mathbf{C}_{22} &= \mathbf{h}_2 + \mathbf{h}_3 - \mathbf{h}_4 - \mathbf{h}_5 + \mathbf{h}_7 - \mathbf{h}_8 \\
\mathbf{C}_{13} &= -\mathbf{h}_{13} + \mathbf{h}_{15} + \mathbf{h}_7 - \mathbf{h}_8 - \mathbf{h}_9 \\
\mathbf{C}_{23} &= \mathbf{h}_{11} - \mathbf{h}_{12} + \mathbf{h}_{13} + \mathbf{h}_{14} + \mathbf{h}_8
\end{aligned}$$

We can apply the high-level algorithm for multiplying block matrices,

$$\left(\begin{array}{c|c|c} \mathbf{C}_{11} & \mathbf{C}_{12} & \mathbf{C}_{13} \\ \hline \mathbf{C}_{21} & \mathbf{C}_{22} & \mathbf{C}_{23} \end{array}\right) = \left(\begin{array}{c|c|c} \mathbf{A}_{11} & \mathbf{A}_{12} & \mathbf{A}_{13} \\ \hline \mathbf{A}_{21} & \mathbf{A}_{22} & \mathbf{A}_{23} \end{array}\right) \left(\begin{array}{c|c|c} \mathbf{B}_{11} & \mathbf{B}_{12} & \mathbf{B}_{13} \\ \hline \mathbf{B}_{21} & \mathbf{B}_{22} & \mathbf{B}_{23} \\ \hline \mathbf{B}_{31} & \mathbf{B}_{32} & \mathbf{B}_{33} \end{array}\right).$$

Let us fill the block matrices with the elements of the original matrices $\mathbf{A}$ and $\mathbf{B}$ (by padding with zeroes to match the sizes[8]). For example, one can use the following scheme:

$$
\left(\begin{array}{ccc|ccc|ccc}
c_{11} & c_{12} & c_{13} & c_{14} & c_{15} & c_{16} & c_{17} & c_{18} & c_{19} \\
c_{21} & c_{22} & c_{23} & c_{24} & c_{25} & c_{26} & c_{27} & c_{28} & c_{29} \\
c_{31} & c_{32} & c_{33} & c_{34} & c_{35} & c_{36} & c_{37} & c_{38} & c_{39} \\
0 & 0 & 0 & 0 & 0 & 0 & 0 & 0 & 0 \\
0 & 0 & 0 & 0 & 0 & 0 & 0 & 0 & 0 \\
0 & 0 & 0 & 0 & 0 & 0 & 0 & 0 & 0 \\
\hline
c_{41} & c_{42} & c_{43} & c_{44} & c_{45} & c_{46} & c_{47} & c_{48} & c_{49} \\
c_{51} & c_{52} & c_{53} & c_{54} & c_{55} & c_{56} & c_{57} & c_{58} & c_{59} \\
c_{61} & c_{62} & c_{63} & c_{64} & c_{65} & c_{66} & c_{67} & c_{68} & c_{69} \\
c_{71} & c_{72} & c_{73} & c_{74} & c_{75} & c_{76} & c_{77} & c_{78} & c_{79} \\
c_{81} & c_{82} & c_{83} & c_{84} & c_{85} & c_{86} & c_{87} & c_{88} & c_{89} \\
c_{91} & c_{92} & c_{93} & c_{94} & c_{95} & c_{96} & c_{97} & c_{98} & c_{99}
\end{array}\right)
$$
$$
= \left(\begin{array}{ccc|ccc|ccc}
a_{11} & a_{12} & a_{13} & a_{14} & a_{15} & a_{16} & a_{17} & a_{18} & a_{19} \\
a_{21} & a_{22} & a_{23} & a_{24} & a_{25} & a_{26} & a_{27} & a_{28} & a_{29} \\
a_{31} & a_{32} & a_{33} & a_{34} & a_{35} & a_{36} & a_{37} & a_{38} & a_{39} \\
0 & 0 & 0 & 0 & 0 & 0 & 0 & 0 & 0 \\
0 & 0 & 0 & 0 & 0 & 0 & 0 & 0 & 0 \\
0 & 0 & 0 & 0 & 0 & 0 & 0 & 0 & 0 \\
\hline
a_{41} & a_{42} & a_{43} & a_{44} & a_{45} & a_{46} & a_{47} & a_{48} & a_{49} \\
a_{51} & a_{52} & a_{53} & a_{54} & a_{55} & a_{56} & a_{57} & a_{58} & a_{59} \\
a_{61} & a_{62} & a_{63} & a_{64} & a_{65} & a_{66} & a_{67} & a_{68} & a_{69} \\
a_{71} & a_{72} & a_{73} & a_{74} & a_{75} & a_{76} & a_{77} & a_{78} & a_{79} \\
a_{81} & a_{82} & a_{83} & a_{84} & a_{85} & a_{86} & a_{87} & a_{88} & a_{89} \\
a_{91} & a_{92} & a_{93} & a_{94} & a_{95} & a_{96} & a_{97} & a_{98} & a_{99}
\end{array}\right)
\left(\begin{array}{ccc|ccc|ccc}
b_{11} & b_{12} & b_{13} & b_{14} & b_{15} & b_{16} & b_{17} & b_{18} & b_{19} \\
b_{21} & b_{22} & b_{23} & b_{24} & b_{25} & b_{26} & b_{27} & b_{28} & b_{29} \\
b_{31} & b_{32} & b_{33} & b_{34} & b_{35} & b_{36} & b_{37} & b_{38} & b_{39} \\
\hline
b_{41} & b_{42} & b_{43} & b_{44} & b_{45} & b_{46} & b_{47} & b_{48} & b_{49} \\
b_{51} & b_{52} & b_{53} & b_{54} & b_{55} & b_{56} & b_{57} & b_{58} & b_{59} \\
b_{61} & b_{62} & b_{63} & b_{64} & b_{65} & b_{66} & b_{67} & b_{68} & b_{69} \\
\hline
b_{71} & b_{72} & b_{73} & b_{74} & b_{75} & b_{76} & b_{77} & b_{78} & b_{79} \\
b_{81} & b_{82} & b_{83} & b_{84} & b_{85} & b_{86} & b_{87} & b_{88} & b_{89} \\
b_{91} & b_{92} & b_{93} & b_{94} & b_{95} & b_{96} & b_{97} & b_{98} & b_{99}
\end{array}\right). \tag{3}
$$

There are multiple ways in which the elements of $\mathbf{A}$, $\mathbf{B}$, and $\mathbf{C}$ can be placed into the block matrices above, depending on the specific structure of the zero padding. Again, this choice affects the resulting number of factors in the decomposition of $\mathfrak{T}_9$, as described below. In practice, we try all combinations and pick the best one.

We now analyze the steps of the high-level algorithm when applied to the blocks of the matrices in Eq. 3. Step 1, $\mathbf{h}_1 = \mathbf{A}_{23}\left(\mathbf{B}_{21} - \mathbf{B}_{23} - \mathbf{B}_{31}\right)$, involves multiplying a 6×3 matrix by a 3×3 matrix, and thus can be done by using the algorithm $\mathfrak{T}_{6,3,3}$ (which involves 40 multiplications). Steps 2, 3, and 4 are similar. However, step 5 is different: it involves $\mathbf{A}_{12}$, which is of size 6×3; however half of its rows are zero, so we can use $\mathfrak{T}_3$ (for which we have a rank-23 decomposition) for the computations in this step. We can also see that the sparsity pattern of the block matrix $\mathbf{C}$ also affects the resulting number of terms in the decomposition. For example, step 10 of the high-level algorithm only involves dense matrix multiplications and thus one might think that it can only be done via $\mathfrak{T}_{6,3,3}$. However, $\mathbf{h}_{10}$ is only used later for computing $\mathbf{C}_{11}$, whose last three rows are zero, so there is no need to compute the last three rows of h_{10}, and thus it can be done via $\mathfrak{T}_3$.

Exploiting the sparsity patterns in all 15 steps, we obtain that the final rank of the decomposition is 498 (this decomposition involves 6 factorizations of $\mathfrak{T}_3$, and 9 factorizations of $\mathfrak{T}_{6,3,3}$). We refer to the accompanying supplementary data for all the decompositions.

H.2 General approach

For each target $\mathfrak{T}_{n,m,p}$ with $n, m, p \in \{3, \ldots, 12\}$, we try all the factorizations discovered by *AlphaTensor* as high-level factorization, and all non-equivalent ways of placing the original matrix elements into the blocks of the high-level matrices (i.e., the sparsity patterns). Since the results depend on the particular high-level factorization, we are able to leverage the diversity of the decompositions found by *AlphaTensor*.

This generalized divide-and-conquer approach subsumes adding and multiplying factorizations. Indeed, by using the trivial decomposition of $\mathfrak{T}_{1,1,2}$ as the high-level algorithm, one can sum factorizations of $\mathfrak{T}_{n,m,p}$ and $\mathfrak{T}_{n,m,p'}$ into

[8] To apply the high-level matrix multiplication algorithm to the block matrix, we need to pad every block in a matrix to have the same shape so that we can add blocks as specified by the high-level algorithm. Note that this padding is only done as an intermediate step for constructing the factorization; the resulting factorization of $\mathfrak{T}_{9,9,9}$ (and thus the algorithm for multiplying 9×9 matrices) does not involve padding.

a factorization of $\mathfrak{T}_{n,m,p+p'}$. Similarly, by dividing the original matrices $\mathbf{A}$, $\mathbf{B}$ and $\mathbf{C}$ into blocks without introducing any zero padding, one can replicate multiplication of factorizations. For all the results, we refer to Extended Data Table 1.

I Proofs

In this section, we prove the results in Theorem B.2 and Theorem B.3, which are needed to prove Theorem B.1. We also provide a proof for Theorem C.4.

I.1 Proof of Theorem B.2

Proof. By transitivity, applying Φ to two equivalent factorizations preserves their equivalence. Therefore, the two factorizations $\{(\tilde{\mathbf{U}}_r, \tilde{\mathbf{V}}_r, \tilde{\mathbf{W}}_r)\}_{r=1}^R$ and $\{(\tilde{\mathbf{U}}'_r, \tilde{\mathbf{V}}'_r, \tilde{\mathbf{W}}'_r)\}_{r=1}^R$ with $\tilde{\mathbf{U}}_r = \mathbf{U}_r\mathbf{V}_1\mathbf{W}_1$, $\tilde{\mathbf{V}}_r = \mathbf{W}_1^{-1}\mathbf{V}_1^{-1}\mathbf{V}_r\mathbf{W}_1$, $\tilde{\mathbf{W}}_r = \mathbf{W}_1^{-1}\mathbf{W}_r$, and $\tilde{\mathbf{U}}'_r = \mathbf{U}'_r\mathbf{V}'_1\mathbf{W}'_1$, $\tilde{\mathbf{V}}'_r = \mathbf{W}_1'^{-1}\mathbf{V}_1'^{-1}\mathbf{V}'_r\mathbf{W}'_1$, $\tilde{\mathbf{W}}'_r = \mathbf{W}_1'^{-1}\mathbf{W}'_r$ are also equivalent. Hence, there exist $(\mathbf{A}, \mathbf{B}, \mathbf{C}, \pi)$ such that

$$\begin{aligned}
&\mathbf{A}T_\pi(\tilde{\mathbf{U}}_r)\mathbf{B}^{-1} \otimes_K \mathbf{B}T_\pi(\tilde{\mathbf{V}}_r)\mathbf{C}^{-1} \otimes_K \mathbf{C}T_\pi(\tilde{\mathbf{W}}_r)\mathbf{A}^{-1} \\
=&(\mathbf{A} \otimes_K \mathbf{B} \otimes_K \mathbf{C})(T_\pi(\tilde{\mathbf{U}}_r) \otimes_K T_\pi(\tilde{\mathbf{V}}_r) \otimes_K T_\pi(\tilde{\mathbf{W}}_r))(\mathbf{B}^{-1} \otimes_K \mathbf{C}^{-1} \otimes_K \mathbf{A}^{-1}) \\
=&\tilde{\mathbf{U}}'_r \otimes_K \tilde{\mathbf{V}}'_r \otimes_K \tilde{\mathbf{W}}'_r, \qquad (4)
\end{aligned}$$

where T_π is the identity function when π has signature 1, and the transposition function otherwise. By construction $(\tilde{\mathbf{U}}_1, \tilde{\mathbf{V}}_1, \tilde{\mathbf{W}}_1) = (I, I, I)$, so $T_\pi(\tilde{\mathbf{U}}_1) = T_\pi(\tilde{\mathbf{V}}_1) = T_\pi(\tilde{\mathbf{W}}_1) = I$, and for $r = 1$ Eq. 4 reduces to

$$\mathbf{A}\mathbf{B}^{-1} \otimes_K \mathbf{B}\mathbf{C}^{-1} \otimes_K \mathbf{C}\mathbf{A}^{-1} = I \otimes_K I \otimes_K I.$$

Inverting this equality, we obtain

$$\mathbf{B}\mathbf{A}^{-1} \otimes_K \mathbf{C}\mathbf{B}^{-1} \otimes_K \mathbf{A}\mathbf{C}^{-1} = I \otimes_K I \otimes_K I. \qquad (5)$$

Multiplying Eqs. 4 and 5, we obtain:

$$\begin{aligned}
&\mathbf{A}T_\pi(\tilde{\mathbf{U}}_r)\mathbf{A}^{-1} \otimes_K \mathbf{B}T_\pi(\tilde{\mathbf{V}}_r)\mathbf{B}^{-1} \otimes_K \mathbf{C}T_\pi(\tilde{\mathbf{W}}_r)\mathbf{C}^{-1} \\
=&(\mathbf{A} \otimes_K \mathbf{B} \otimes_K \mathbf{C})(T_\pi(\tilde{\mathbf{U}}_r) \otimes_K T_\pi(\tilde{\mathbf{V}}_r) \otimes_K T_\pi(\tilde{\mathbf{W}}_r))(\mathbf{A} \otimes_K \mathbf{B} \otimes_K \mathbf{C})^{-1} \\
=&\tilde{\mathbf{U}}'_r \otimes_K \tilde{\mathbf{V}}'_r \otimes_K \tilde{\mathbf{W}}'_r.
\end{aligned}$$

Using the invariance of the CharPoly function under similarities, we obtain

$$\text{CharPoly}(T_\pi(\tilde{\mathbf{U}}_r) \otimes_K T_\pi(\tilde{\mathbf{V}}_r) \otimes_K T_\pi(\tilde{\mathbf{W}}_r)) = \text{CharPoly}(\tilde{\mathbf{U}}'_r \otimes_K \tilde{\mathbf{V}}'_r \otimes_K \tilde{\mathbf{W}}'_r).$$

The eigenvalues and therefore also the characteristic polynomial of a matrix are also invariant under transposition, so $\text{CharPoly}(T_\pi(\tilde{\mathbf{U}}_r) \otimes_K T_\pi(\tilde{\mathbf{V}}_r) \otimes_K T_\pi(\tilde{\mathbf{W}}_r)) = \text{CharPoly}(\tilde{\mathbf{U}}_r \otimes_K \tilde{\mathbf{V}}_r \otimes_K \tilde{\mathbf{W}}_r)$ and we conclude that

$$\text{CharPoly}(\tilde{\mathbf{U}}_r \otimes_K \tilde{\mathbf{V}}_r \otimes_K \tilde{\mathbf{W}}_r) = \text{CharPoly}(\tilde{\mathbf{U}}'_r \otimes_K \tilde{\mathbf{V}}'_r \otimes_K \tilde{\mathbf{W}}'_r). \qquad \square$$

I.2 Proof of Theorem B.3

Before we prove Theorem B.3, we need the following two lemmas:

Lemma I.1. *If* $\{(\mathbf{X}_p, \mathbf{Y}_p, \mathbf{Z}_p)\}_{p=1}^R \overset{\mathbf{A},\mathbf{B},\mathbf{C},\pi}{\sim} \{(\mathbf{F}_r^1, \mathbf{F}_r^2, \mathbf{F}_r^3)\}_{r=1}^R$ *and* $\{(\mathbf{X}'_q, \mathbf{Y}'_q, \mathbf{Z}'_q)\}_{q=1}^R \overset{\mathbf{A}',\mathbf{B}',\mathbf{C}',\pi'}{\sim} \{(\mathbf{F}_r^1, \mathbf{F}_r^2, \mathbf{F}_r^3)\}_{r=1}^R$ *then*

$$\begin{aligned}
&\left\{(\mathbf{X}_p \otimes_K \mathbf{X}'_q, \mathbf{Y}_p \otimes_K \mathbf{Y}'_q, \mathbf{Z}_p \otimes_K \mathbf{Z}'_q)\right\}_{p,q=1}^R \\
\overset{\mathbf{A}\otimes\mathbf{A}',\mathbf{B}\otimes\mathbf{B}',\mathbf{C}\otimes\mathbf{C}',id}{\sim}&\left\{\left(T_\pi(\mathbf{F}_p^{\pi(1)}) \otimes_K T_{\pi'}(\mathbf{F}_q^{\pi'(1)}), T_\pi(\mathbf{F}_p^{\pi(2)}) \otimes_K T_{\pi'}(\mathbf{F}_q^{\pi'(2)}), T_\pi(\mathbf{F}_p^{\pi(3)}) \otimes_K T_{\pi'}(\mathbf{F}_q^{\pi'(3)})\right)\right\}_{p,q=1}^R.
\end{aligned}$$

Proof. By assumption we have

$$\mathbf{X}_p \otimes \mathbf{Y}_p \otimes \mathbf{Z}_p = (\mathbf{A}T_\pi(\mathbf{F}_p^{\pi(1)})\mathbf{B}^{-1}) \otimes (\mathbf{B}T_\pi(\mathbf{F}_p^{\pi(2)})\mathbf{C}^{-1}) \otimes (\mathbf{C}T_\pi(\mathbf{F}_p^{\pi(3)})\mathbf{A}^{-1}),$$
$$\mathbf{X}'_q \otimes \mathbf{Y}'_q \otimes \mathbf{Z}'_q = (\mathbf{A}'T_{\pi'}(\mathbf{F}_q^{\pi'(1)})\mathbf{B}'^{-1}) \otimes (\mathbf{B}'T_{\pi'}(\mathbf{F}_q^{\pi'(2)})\mathbf{C}'^{-1}) \otimes (\mathbf{C}'T_{\pi'}(\mathbf{F}_q^{\pi'(3)})\mathbf{A}'^{-1}).$$

Multiplying these two identities, we obtain

$$\begin{aligned}(\mathbf{X}_p \otimes_K \mathbf{X}'_q) \otimes (\mathbf{Y}_p \otimes_K \mathbf{Y}'_q) \otimes (\mathbf{Z}_p \otimes_K \mathbf{Z}'_q) &= (\mathbf{X}_p \otimes \mathbf{Y}_p \otimes \mathbf{Z}_p) \otimes_K (\mathbf{X}'_q \otimes \mathbf{Y}'_q \otimes \mathbf{Z}'_q) \\
&= \Big((\mathbf{A}T_\pi(\mathbf{F}_p^{\pi(1)})\mathbf{B}^{-1}) \otimes_K (\mathbf{A}'T_{\pi'}(\mathbf{F}_q^{\pi'(1)})\mathbf{B}'^{-1})\Big) \\
&\quad \otimes \Big((\mathbf{B}T_\pi(\mathbf{F}_p^{\pi(2)})\mathbf{C}^{-1}) \otimes_K (\mathbf{B}'T_{\pi'}(\mathbf{F}_q^{\pi'(2)})\mathbf{C}'^{-1})\Big) \\
&\quad \otimes \Big((\mathbf{C}T_\pi(\mathbf{F}_p^{\pi(3)})\mathbf{A}^{-1}) \otimes_K (\mathbf{C}'T_{\pi'}(\mathbf{F}_q^{\pi'(3)})\mathbf{A}'^{-1})\Big) \\
&= \Big([\mathbf{A} \otimes_K \mathbf{A}'] \Big[T_\pi(\mathbf{F}_p^{\pi(1)}) \otimes_K T_{\pi'}(\mathbf{F}_q^{\pi'(1)})\Big] [\mathbf{B} \otimes_K \mathbf{B}']^{-1}\Big) \\
&\quad \otimes \Big([\mathbf{B} \otimes_K \mathbf{B}'] \Big[T_\pi(\mathbf{F}_p^{\pi(2)}) \otimes_K T_{\pi'}(\mathbf{F}_q^{\pi'(2)})\Big] [\mathbf{C} \otimes_K \mathbf{C}']^{-1}\Big) \\
&\quad \otimes \Big([\mathbf{C} \otimes_K \mathbf{C}'] \Big[T_\pi(\mathbf{F}_p^{\pi(3)}) \otimes_K T_{\pi'}(\mathbf{F}_q^{\pi'(3)})\Big] [\mathbf{A} \otimes_K \mathbf{A}']^{-1}\Big),\end{aligned}$$

where we used the identities $(\mathbf{AC}) \otimes_K (\mathbf{BD}) = (\mathbf{A} \otimes_K \mathbf{B})(\mathbf{C} \otimes_K \mathbf{D})$ and $(\mathbf{B} \otimes_K \mathbf{B}')^{-1} = (\mathbf{B}^{-1} \otimes_K \mathbf{B}'^{-1})$. □

Lemma I.2. *Let $\mathfrak{T}$ be a tensor with nonnegative entries and of rank $R > 0$, and let $\{(\mathbf{X}_p \otimes_K \mathbf{X}'_q, \mathbf{Y}_p \otimes_K \mathbf{Y}'_q, \mathbf{Z}_p \otimes_K \mathbf{Z}'_q)\}_{p,q=1}^R$ be a factorization of $\mathfrak{T}^{\otimes 2}$. Then, there exist α, β with $\alpha\beta = 1$ such that both $\{(\alpha\mathbf{X}_p, \alpha\mathbf{Y}_p, \alpha\mathbf{Z}_p)\}_{p=1}^R$ and $\{(\beta\mathbf{X}'_q, \beta\mathbf{Y}'_q, \beta\mathbf{Z}'_q)\}_{q=1}^R$ are valid factorizations of $\mathfrak{T}$.*

Proof. Since $\left\{(\mathbf{X}_p \otimes_K \mathbf{X}'_q, \mathbf{Y}_p \otimes_K \mathbf{Y}'_q, \mathbf{Z}_p \otimes_K \mathbf{Z}'_q)\right\}_{p,q=1}^R$ is a factorization of $\mathfrak{T}^{\otimes 2}$, we have:

$$\mathfrak{T}^{\otimes 2}[i_1 + ni'_1, i_2 + ni'_2, j_1 + nj'_1, j_2 + nj'_2, k_1 + nk'_1, k_2 + nk'_2] \tag{6}$$
$$= \sum_{p=1}^R \sum_{q=1}^R (\mathbf{X}_p \otimes_K \mathbf{X}'_q)[i_1 + ni'_1, i_2 + ni'_2](\mathbf{Y}_p \otimes_K \mathbf{Y}'_q)[j_1 + nj'_1, j_2 + nj'_2](\mathbf{Z}_p \otimes_K \mathbf{Z}'_q)[k_1 + nk'_1, k_2 + nk'_2] \tag{7}$$
$$= \left(\sum_{p=1}^R \mathbf{X}_p[i_1, i_2]\mathbf{Y}_p[j_1, j_2]\mathbf{Z}_p[k_1, k_2]\right)\left(\sum_{q=1}^R \mathbf{X}'_q[i'_1, i'_2]\mathbf{Y}'_q[j'_1, j'_2]\mathbf{Z}'_q[k'_1, k'_2]\right) \tag{8}$$

Hence, we obtain

$$\sum_{i'_1, i'_2, j'_1, j'_2, k'_1, k'_2} \mathfrak{T}^{\otimes 2}[i_1 + ni'_1, i_2 + ni'_2, j_1 + nj'_1, j_2 + nj'_2, k_1 + nk'_1, k_2 + nk'_2] \tag{9}$$
$$= \mathfrak{T}[i_1, i_2, j_1, j_2, k_1, k_2] \sum_{i'_1, i'_2, j'_1, j'_2, k'_1, k'_2} \mathfrak{T}[i'_1, i'_2, j'_1, j'_2, k'_1, k'_2] \tag{10}$$
$$= \left(\sum_{p=1}^R \mathbf{X}_p[i_1, i_2]\mathbf{Y}_p[j_1, j_2]\mathbf{Z}_p[k_1, k_2]\right)\left(\sum_{i'_1, i'_2, j'_1, j'_2, k'_1, k'_2} \sum_{q=1}^R \mathbf{X}'_q[i'_1, i'_2]\mathbf{Y}'_q[j'_1, j'_2]\mathbf{Z}'_q[k'_1, k'_2]\right), \tag{11}$$

from which we deduce that

$$\alpha^{-1}\mathfrak{T}[i_1, i_2, j_1, j_2, k_1, k_2] = \sum_{p=1}^R \mathbf{X}_p[i_1, i_2]\mathbf{Y}_p[j_1, j_2]\mathbf{Z}_p[k_1, k_2],$$

where

$$\alpha^{-1} = \frac{\sum_{i'_1, i'_2, j'_1, j'_2, k'_1, k'_2} \mathfrak{T}[i'_1, i'_2, j'_1, j'_2, k'_1, k'_2]}{\sum_{i'_1, i'_2, j'_1, j'_2, k'_1, k'_2} \sum_{q=1}^R \mathbf{X}'_q[i'_1, i'_2]\mathbf{Y}'_q[j'_1, j'_2]\mathbf{Z}'_q[k'_1, k'_2]}.$$

Note that the denominator is nonzero, as otherwise the tensor $\mathfrak{T}$ is equal to zero. Similarly, we have

$$\beta^{-1}\mathfrak{T}[i_1', i_2', j_1', j_2', k_1', k_2'] = \sum_{q=1}^{R} \mathbf{X}_q'[i_1', i_2']\mathbf{Y}_q'[j_1', j_2']\mathbf{Z}_p'[k_1', k_2'],$$

$$\beta^{-1} = \frac{\sum_{i_1,i_2,j_1,j_2,k_1,k_2} \mathfrak{T}[i_1, i_2, j_1, j_2, k_1, k_2]}{\sum_{i_1,i_2,j_1,j_2,k_1,k_2} \sum_{p=1}^{R} \mathbf{X}_p[i_1, i_2]\mathbf{Y}_p[j_1, j_2]\mathbf{Z}_p[k_1, k_2]}.$$

Computing $\alpha^{-1}\beta^{-1}$, we obtain

$$\alpha^{-1}\beta^{-1} = \frac{\sum_{i_1,i_2,j_1,j_2,k_1,k_2} \sum_{i_1',i_2',j_1',j_2',k_1',k_2'} \mathfrak{T}[i_1, i_2, j_1, j_2, k_1, k_2]\mathfrak{T}[i_1', i_2', j_1', j_2', k_1', k_2']}{\left(\sum_{i_1,i_2,j_1,j_2,k_1,k_2} \sum_{p=1}^{R} \mathbf{X}_p[i_1, i_2]\mathbf{Y}_p[j_1, j_2]\mathbf{Z}_p[k_1, k_2]\right)\left(\sum_{i_1',i_2',j_1',j_2',k_1',k_2'} \sum_{q=1}^{R} \mathbf{X}_q'[i_1', i_2']\mathbf{Y}_q'[j_1', j_2']\mathbf{Z}_q'[k_1', k_2']\right)},$$

which is equal to 1 by summing Eq. 8 over all (i, j, k, i', j', k'). □

We now prove Theorem B.3.

Proof. We have $2 \implies 1$. We now prove that $1 \implies 2$. $\{(\mathbf{X}_p \otimes_K \mathbf{X}_q', \mathbf{Y}_p \otimes_K \mathbf{Y}_q', \mathbf{Z}_p \otimes_K \mathbf{Z}_q')\}_{p,q=1}^7$ is a factorization of $\mathfrak{T}_4$ of rank 49. Using Lemma I.2, there exists α, β such that $\{(\alpha\mathbf{X}_p, \alpha\mathbf{Y}_p, \alpha\mathbf{Z}_p)\}_{p=1}^7$ and $\{(\beta\mathbf{X}_q', \beta\mathbf{Y}_q', \beta\mathbf{Z}_q')\}_{q=1}^7$ are both valid factorizations of $\mathfrak{T}_2$ of rank 7, where $\alpha\beta = 1$. We know that all rank 7 factorizations of $\mathfrak{T}_2$ are equivalent [3]; hence, we have

$$\{(\alpha\mathbf{X}_p, \alpha\mathbf{Y}_p, \alpha\mathbf{Z}_p)\}_{p=1}^7 \sim \{(\mathbf{S}_p^1, \mathbf{S}_p^2, \mathbf{S}_p^3)\}_{p=1}^7,$$
$$\{(\beta\mathbf{X}_q', \beta\mathbf{Y}_q', \beta\mathbf{Z}_q')\}_{q=1}^7 \sim \{(\mathbf{S}_p^1, \mathbf{S}_p^2, \mathbf{S}_p^3)\}_{p=1}^7,$$

where we recall that $\{(\mathbf{S}_p^1, \mathbf{S}_p^2, \mathbf{S}_p^3)\}_{p=1}^7$ is a Strassen factorization of $\mathfrak{T}_2$. Using Lemma I.1, there exist permutations π and π' for which the following holds:

$$\begin{aligned}&\{(\mathbf{X}_p \otimes_K \mathbf{X}_q', \mathbf{Y}_p \otimes_K \mathbf{Y}_q', \mathbf{Z}_p \otimes_K \mathbf{Z}_q')\}_{p,q=1}^R \\ \sim &\left\{\left(T_\pi(\mathbf{S}_p^{\pi(1)}) \otimes_K T_{\pi'}(\mathbf{S}_q^{\pi'(1)}), T_\pi(\mathbf{S}_p^{\pi(2)}) \otimes_K T_{\pi'}(\mathbf{S}_q^{\pi'(2)}), T_\pi(\mathbf{S}_p^{\pi(3)}) \otimes_K T_{\pi'}(\mathbf{S}_q^{\pi'(3)})\right)\right\}_{p,q=1}^7,\end{aligned}$$

where we have used the property that $\alpha\beta = 1$. By transitivity of the equivalence relation, we obtain that

$$\{(\mathbf{U}_r, \mathbf{V}_r, \mathbf{W}_r)\}_{r=1}^{49} \sim \left\{\left(T_\pi(\mathbf{S}_p^{\pi(1)}) \otimes_K T_{\pi'}(\mathbf{S}_q^{\pi'(1)}), T_\pi(\mathbf{S}_p^{\pi(2)}) \otimes_K T_{\pi'}(\mathbf{S}_q^{\pi'(2)}), T_\pi(\mathbf{S}_p^{\pi(3)}) \otimes_K T_{\pi'}(\mathbf{S}_q^{\pi'(3)})\right)\right\}_{p,q=1}^7,$$

which concludes the proof. □

I.3 Proof for skew-symmetric matrix-vector product

We now prove Theorem C.4. We start with a simple, but useful result:

Lemma I.3. *Let Ξ be any $n \times n$ matrix, $\Xi = [\xi_r^c]_r^c$. Then the following equality always holds*

$$\sum_{r=1}^{n}\sum_{c=1}^{r} \xi_r^c = \sum_{c=1}^{n}\sum_{r=c}^{n} \xi_r^c \tag{12}$$

Proof. Both sides of the equation sum up the lower triangular part of the matrix $\{\Xi_r^c \mid 1 \leq c \leq r \leq n\}$. □

I.3.1 Problem formulation and the results

Consider an $n \times n$ antisymmetric matrix $\mathbf{A} = [a_r^c]_{r=1,\dots n}^{c=1,\dots n}$, $a_r^c = -a_c^r$ for $r = 1,\dots,n$, $c = 1,\dots,n$. Let $\mathbf{b}$ be an n-dimensional vector $[b_1,\dots,b_n]^\intercal$. We want to compute the following:

$$y_{\bar{r}} \quad := \quad \sum_{c=1}^{n} b_c a_{\bar{r}}^c \quad \text{for } \bar{r} = 1,\dots,n.$$

What we aim to do in this proof is to re-express the previous expressions as a function of the following two kinds of terms:

$$\begin{aligned} \omega_{\bar{r}}^{\bar{c}} \quad &:= \quad a_{\bar{r}}^{\bar{c}} b_{\bar{c}} + a_{\bar{c}}^{\bar{r}} b_{\bar{r}} \quad \text{for } \bar{r} = 1,\dots,n,\ \bar{c} = 1,\dots,n, \\ \beta_{\bar{r}} \quad &:= \quad b_{\bar{r}} \sum_{r=1}^{n} a_r^{\bar{r}} \quad \text{for } \bar{r} = 1,\dots,n, \end{aligned}$$

Remark 1. *Each of the terms $\omega_{\bar{r}}^{\bar{c}}$ and $\beta_{\bar{r}}$ requires precisely one multiplication to compute.*

Proof. The term $\beta_{\bar{r}}$ requires only one multiplication directly by the definition.
There holds $\omega_{\bar{r}}^{\bar{c}} = a_{\bar{r}}^{\bar{c}} b_{\bar{c}} + a_{\bar{c}}^{\bar{r}} b_{\bar{r}} = a_{\bar{r}}^{\bar{c}} (b_{\bar{c}} - b_{\bar{r}})$ as $a_{\bar{r}}^{\bar{c}} = -a_{\bar{c}}^{\bar{r}}$ for $\bar{r} = 1,\dots,n,\ \bar{c} = 1,\dots,n$, which completes the proof. □

We start with a simple observation:

Lemma I.4. *For all $\bar{r}$, $\bar{c}$ there holds*

$$\omega_{\bar{r}}^{\bar{c}} = \omega_{\bar{c}}^{\bar{r}}, \tag{13}$$

$$\omega_{\bar{r}}^{\bar{r}} = 0. \tag{14}$$

This implies that there are only $n(n-1)/2$ different terms $\omega_{\bar{r}}^{\bar{c}}$, which together with the n terms in $\beta_{\bar{r}}$ make $n(n+1)/2$ terms, each of those requiring only one multiplication.

Lemma I.5. *There holds*

$$y_{\bar{r}} = \sum_{c=1}^{n} \omega_{\bar{r}}^{c} - \beta_{\bar{r}}$$

Proof. By definition

$$\sum_{c=1}^{n} \omega_{\bar{r}}^{c} = \sum_{c=1}^{n} a_{\bar{r}}^{c} b_c + a_c^{\bar{r}} b_{\bar{r}} = \sum_{c=1}^{n} a_{\bar{r}}^{c} b_c + \sum_{c=1}^{n} a_c^{\bar{r}} b_{\bar{r}} = y_{\bar{r}} + \beta_{\bar{r}}.$$

□

The previous lemma allows us to prove that we need $n(n+1)/2$ scalar multiplications to multiply a skew-symmetric matrix and a vector. In the following we will go beyond this by leaving one of these terms out, in particular ω_{n-1}^{n}, leading to $n(n+1)/2 - 1 = (n+2)(n-1)/2$ required scalar multiplications.

Corollary I.1. *There holds*

$$y_{\bar{r}} = \sum_{r=1}^{\bar{r}-1} \omega_r^{\bar{r}} + \sum_{c=\bar{r}+1}^{n} \omega_{\bar{r}}^{c} - \beta_{\bar{r}}$$

Note that this corollary allows us to express $y_1, \dots y_{n-2}$ in terms of ω and β elements without using ω_{n-1}^{n}.

Lemma I.6. *There holds*

$$\sum_{r=1}^{n} \sum_{c=1}^{r} \omega_r^c = \sum_{r=1}^{n} \sum_{c=r}^{n} \omega_r^c = \sum_{r=1}^{n} \beta_r.$$

$$
y_4 = \begin{bmatrix}
0 & \omega_1^2 & \omega_1^3 & \omega_1^4 & \omega_1^5 & \omega_1^6 & \omega_1^7 & \omega_1^8 \\
\omega_2^1 & 0 & \omega_2^3 & \omega_2^4 & \omega_2^5 & \omega_2^6 & \omega_2^7 & \omega_2^8 \\
\omega_3^1 & \omega_3^2 & 0 & \omega_3^4 & \omega_3^5 & \omega_3^6 & \omega_3^7 & \omega_3^8 \\
\omega_4^1 & \omega_4^2 & \omega_4^3 & 0 & \omega_4^5 & \omega_4^6 & \omega_4^7 & \omega_4^8 \\
\omega_5^1 & \omega_5^2 & \omega_5^3 & \omega_5^4 & 0 & \omega_5^6 & \omega_5^7 & \omega_5^8 \\
\omega_6^1 & \omega_6^2 & \omega_6^3 & \omega_6^4 & \omega_6^5 & 0 & \omega_6^7 & \omega_6^8 \\
\omega_7^1 & \omega_7^2 & \omega_7^3 & \omega_7^4 & \omega_7^5 & \omega_7^6 & 0 & \omega_7^8 \\
\omega_8^1 & \omega_8^2 & \omega_8^3 & \omega_8^4 & \omega_8^5 & \omega_8^6 & \omega_8^7 & 0
\end{bmatrix}
-
\begin{bmatrix}
\beta_1 \\ \beta_2 \\ \beta_3 \\ \beta_4 \\ \beta_5 \\ \beta_6 \\ \beta_7 \\ \beta_8
\end{bmatrix}
$$

Figure I.5: Two alternative expressions for y_4. We can either use blue and gray terms (Lemma I.5), or green and gray terms (Corollary I.1).

Proof. We use the identity $\omega_r^c = \omega_c^r$ to show the first equality

$$\sum_{r=1}^{n}\sum_{c=1}^{r}\omega_r^c = \underbrace{\sum_{r=1}^{n}\sum_{c=1}^{r}\omega_c^r = \sum_{c=1}^{n}\sum_{r=c}^{n}\omega_c^r}_{\text{by Lemma I.3}} = \sum_{r=1}^{n}\sum_{c=r}^{n}\omega_r^c.$$

Now it remains to prove that $\sum_{r=1}^{n}\sum_{c=1}^{r}\omega_r^c = \sum_{r=1}^{n}\beta_r$.

$$\sum_{r=1}^{n}\sum_{c=1}^{r}\omega_r^c = \sum_{r=1}^{n}\sum_{c=1}^{r}a_r^c b_c + a_c^r b_r = \sum_{r=1}^{n}\sum_{c=1}^{r}a_r^c b_c + \sum_{r=1}^{n}\sum_{c=1}^{r}a_c^r b_r =$$
$$\sum_{r=1}^{n}\sum_{c=1}^{r}a_r^c b_c + \sum_{c=1}^{n}\sum_{r=c}^{n}a_c^r b_r = \sum_{r=1}^{n}\sum_{c=1}^{r}a_r^c b_c + \sum_{r=1}^{n}\sum_{c=r}^{n}a_r^c b_c = \sum_{r=1}^{n}\sum_{c=1}^{n}a_r^c b_c + \sum_{r=1}^{n}a_r^r b_r.$$

But $a_r^r = 0$ for all $r = 1, \ldots, n$, due to antisymmetry, thus

$$\sum_{r=1}^{n}\sum_{c=r}^{n}\omega_r^c = \sum_{r=1}^{n}\sum_{c=1}^{n}a_r^c b_c = \sum_{c=1}^{n}b_c\sum_{r=1}^{n}a_r^c = \sum_{c=1}^{n}\beta_c.$$

□

Proposition 1. *The following equalities hold*

$$y_{\bar{r}} = -\sum_{r=1, r\neq\bar{r}}^{n}\;\sum_{c=r, c\neq\bar{r}}^{n}\omega_r^c + \sum_{r=1, r\neq\bar{r}}^{n}\beta_r, \tag{15}$$

$$y_{\bar{r}} = -\sum_{r=1, r\neq\bar{r}}^{n}\;\sum_{c=1, c\neq\bar{r}}^{r}\omega_r^c + \sum_{r=1, r\neq\bar{r}}^{n}\beta_r \tag{16}$$

Proof. By Lemmas I.5 and I.6, there holds

$$y_{\bar r} = \sum_{c=1}^{n} \omega_{\bar r}^c - \beta_{\bar r} = \sum_{c=1}^{n} \omega_{\bar r}^c - \beta_{\bar r} \underbrace{- \sum_{r=1}^{n}\sum_{c=r}^{n} \omega_r^c + \sum_{r=1}^{n} \beta_r}_{=0 \text{ by Lemma I.6}} =$$

$$\sum_{c=1}^{n} \omega_{\bar r}^c - \sum_{r=1}^{n}\sum_{c=r}^{n} \omega_r^c + \sum_{r=1, r\neq \bar r}^{n} \beta_r =$$

$$\sum_{c=1}^{\bar r - 1} \omega_{\bar r}^c - \sum_{r=1, r\neq \bar r}^{n}\sum_{c=r}^{n} \omega_r^c + \sum_{r=1, r\neq \bar r}^{n} \beta_r =$$

$$\sum_{r=1}^{\bar r - 1} \omega_r^{\bar r} - \sum_{r=1, r\neq \bar r}^{n}\sum_{c=r}^{n} \omega_n^c + \sum_{r=1, r\neq \bar r}^{n} \beta_r = - \sum_{r=1, r\neq \bar r}^{n}\sum_{c=r, c\neq \bar r}^{n} \omega_r^c + \sum_{r=1, r\neq \bar r}^{n} \beta_r.$$

Analogously

$$y_{\bar r} = \sum_{c=1}^{n} \omega_{\bar r}^c - \beta_{\bar r} = \sum_{c=1}^{n} \omega_{\bar r}^c - \beta_{\bar r} \underbrace{- \sum_{r=1}^{n}\sum_{c=1}^{i} \omega_r^c + \sum_{r=1}^{n} \beta_r}_{=0 \text{ by Lemma I.6}} =$$

$$\sum_{c=1}^{n} \omega_{\bar r}^c - \sum_{r=1}^{n}\sum_{c=1}^{r} \omega_r^c + \sum_{r=1, r\neq \bar r}^{n} \beta_r =$$

$$\sum_{c=\bar r + 1}^{n} \omega_{\bar r}^c - \sum_{r=1, r\neq \bar r}^{n}\sum_{c=1}^{r} \omega_r^c + \sum_{r=1, r\neq \bar r}^{n} \beta_r = - \sum_{r=1, r\neq \bar r}^{n}\sum_{c=1, c\neq \bar r}^{r} \omega_r^c + \sum_{r=1, r\neq \bar r}^{n} \beta_r.$$

□

Corollary I.2. *We have*

$$y_{n-1} = - \sum_{r=1}^{n-2}\sum_{c=r}^{n-2} \omega_r^c - \sum_{c=1}^{n-2} \omega_n^c + \sum_{r=1, r\neq n-1}^{n} \beta_r$$

$$y_n = - \sum_{r=1}^{n-1}\sum_{c=1}^{r} \omega_r^c + \sum_{r=1}^{n-1} \beta_r$$

Proof. Using Proposition 1 (Eq. 15), we get

$$y_{n-1} = - \sum_{r=1, r\neq n-1}^{n}\sum_{c=r, c\neq n-1}^{n} \omega_r^c + \sum_{r=1, r\neq n-1}^{n} \beta_r =$$

$$- \sum_{r=1}^{n-2}\sum_{c=r}^{n-2} \omega_r^c - \sum_{c=1}^{n-2} \omega_n^c + \sum_{r=1, r\neq n-1}^{n} \beta_r$$

The second equality is a straigthforward application of Proposition 1 (Eq. 16) with $\bar r = n$. □

Theorem I.5. *The operation of a multiplication of a $n \times n$ antisymmetric matrix and a vector can be expressed using $\frac{n(n+1)}{2} - 1$ multiplications.*

Proof. Recall (Remark 1), that each of the terms $\omega_{\bar r}^{\bar c}$ and $\beta_{\bar r}$ can be computed using one multiplication. Thanks to Lemma I.4 we know that there are only $\frac{n(n-1)}{2}$ distinct terms $\omega_{\bar r}^{\bar c}$. In addition, the formulas require n values of $\beta_{\bar r}$, each of them requiring 1 multiplication.

Note that thanks to Corollary I.1 we can compute $y_1 \dots y_{n-2}$ using $\frac{n(n-1)}{2} - 1$ (instead of $\frac{n(n-1)}{2}$) terms $\omega_{\bar r}^{\bar c}$, in particular not incorporating ω_{n-1}^n.

Finally, observe that the formulas in Corollary I.2 do not incorporate the term ω_{n-1}^n either, which ends the proof. □

References

[1] Marijn JH Heule, Manuel Kauers, and Martina Seidl. New ways to multiply 3× 3-matrices. *Journal of Symbolic Computation*, 104:899–916, 2021.

[2] Shmuel Winograd. A new algorithm for inner product. *IEEE Transactions on Computers*, 100(7):693–694, 1968.

[3] Hans F de Groote. On varieties of optimal algorithms for the computation of bilinear mappings ii. optimal algorithms for 2× 2-matrix multiplication. *Theoretical Computer Science*, 7(2):127–148, 1978.

[4] Vladimir P Burichenko. Symmetries of matrix multiplication algorithms. i. *arXiv preprint arXiv:1508.01110*, 2015.

[5] Joseph M Landsberg. *Geometry and complexity theory*, volume 169. Cambridge University Press, 2017.

[6] Luca Chiantini, Christian Ikenmeyer, Joseph M Landsberg, and Giorgio Ottaviani. The geometry of rank decompositions of matrix multiplication I: 2× 2 matrices. *Experimental Mathematics*, 28(3):322–327, 2019.

[7] Grey Ballard, Christian Ikenmeyer, Joseph M Landsberg, and Nick Ryder. The geometry of rank decompositions of matrix multiplication II: 3× 3 matrices. *Journal of Pure and Applied Algebra*, 223(8):3205–3224, 2019.

[8] Rodney W. Johnson and A. McLoughlin. Noncommutative bilinear algorithms for 3 x 3 matrix multiplication. *SIAM J. Comput.*, 15:595–603, 1986.

[9] Alexandre Sedoglavic and Alexey V Smirnov. The tensor rank of 5x5 matrices multiplication is bounded by 98 and its border rank by 89. In *Proceedings of the 2021 on International Symposium on Symbolic and Algebraic Computation*, pages 345–351, 2021.

[10] Grey Ballard, Austin R. Benson, Alex Druinsky, Benjamin Lipshitz, and Oded Schwartz. Improving the numerical stability of fast matrix multiplication. *SIAM Journal on Matrix Analysis and Applications*, 37(4):1382–1418, 2016.

[11] Zhen Dai and Lek-Heng Lim. Numerical stability and tensor nuclear norm. *arXiv preprint arXiv:2207.08769*, 2022.

[12] Peter W Battaglia, Jessica B Hamrick, Victor Bapst, Alvaro Sanchez-Gonzalez, Vinicius Zambaldi, Mateusz Malinowski, Andrea Tacchetti, David Raposo, Adam Santoro, Ryan Faulkner, et al. Relational inductive biases, deep learning, and graph networks. *arXiv preprint arXiv:1806.01261*, 2018.

[13] Matej Balog, Bart van Merriënboer, Subhodeep Moitra, Yujia Li, and Daniel Tarlow. Fast training of sparse graph neural networks on dense hardware. *arXiv preprint arXiv:1906.11786*, 2019.

[14] Ke Ye and Lek-Heng Lim. Fast structured matrix computations: tensor rank and Cohn–Umans method. *Foundations of Computational Mathematics*, 18(1):45–95, 2018.

[15] Will Dabney, Georg Ostrovski, David Silver, and Rémi Munos. Implicit quantile networks for distributional reinforcement learning. In *International conference on machine learning*, pages 1096–1105. PMLR, 2018.

[16] James Bradbury, Roy Frostig, Peter Hawkins, Matthew James Johnson, Chris Leary, Dougal Maclaurin, George Necula, Adam Paszke, Jake VanderPlas, Skye Wanderman-Milne, and Qiao Zhang. JAX: composable transformations of Python+NumPy programs, 2018.

[17] Alex Martelli, Anna Ravenscroft, and David Ascher. *Python cookbook*. " O'Reilly Media, Inc.", 2005.

[18] timeit – Measure execution time of small code snippets. https://docs.python.org/3/library/timeit.html.

[19] Vin De Silva and Lek-Heng Lim. Tensor rank and the ill-posedness of the best low-rank approximation problem. *SIAM Journal on Matrix Analysis and Applications*, 30(3):1084–1127, 2008.

[20] Dario Andrea Bini, Milvio Capovani, Francesco Romani, and Grazia Lotti. O(n2.7799) complexity for n by n approximate matrix multiplication. *Information Processing Letters*, 1979.

[21] Alexey V Smirnov. The bilinear complexity and practical algorithms for matrix multiplication. *Computational Mathematics and Mathematical Physics*, 53(12):1781–1795, 2013.

[22] Austin Conner, Alicia Harper, and Joseph M Landsberg. New lower bounds for matrix multiplication and the 3x3 determinant. *arXiv preprint arXiv:1911.07981*, 2019.

[23] Julian Schrittwieser, Ioannis Antonoglou, Thomas Hubert, Karen Simonyan, Laurent Sifre, Simon Schmitt, Arthur Guez, Edward Lockhart, Demis Hassabis, Thore Graepel, et al. Mastering atari, go, chess and shogi by planning with a learned model. *Nature*, 588(7839):604–609, 2020.

[24] Ilya Loshchilov and Frank Hutter. Decoupled weight decay regularization. In *International Conference on Learning Representations (ICLR)*, 2019.

[25] Alexandre Sedoglavic. A non-commutative algorithm for multiplying (7×7) matrices using 250 multiplications. *arXiv preprint arXiv:1712.07935*, 2017.

[26] Charles-Éric Drevet, Md Nazrul Islam, and Éric Schost. Optimization techniques for small matrix multiplication. *Theoretical Computer Science*, 412(22):2219–2236, 2011.
